# A novel metabolism-related gene signature in patients with hepatocellular carcinoma

**DOI:** 10.7717/peerj.16335

**Published:** 2023-11-09

**Authors:** Bin Ru, Jiaqi Hu, Nannan Zhang, Quan Wan

**Affiliations:** 1 Department of Pain Management, Zhejiang Provincial People’s Hospital (Affiliated People’s Hospital, Hangzhou Medical College), Hangzhou, Zhejiang, China; 2 State Key Laboratory of Medical Neurobiology and MOE Frontiers Center for Brain Science, Fudan University, Shanghai, China; 3 Department of Physiology and Neurobiology, School of Life Sciences, Fudan University, Shanghai, China

**Keywords:** Hepatocellular carcinoma (HCC), Lipid metabolism, Biomarkers, Risk model, ICGC, GOT2

## Abstract

Hepatocellular carcinoma (HCC) remains a global challenge as it is the sixth most common neoplasm worldwide and the third leading cause of cancer-related death. A key feature of HCC is abnormal metabolism, which promotes cancer cell proliferation, survival, invasion, and metastasis. However, the significance of metabolism-related genes (MRGs) in HCC remains to be elucidated. Here, we aim to establish a novel metabolism-related prognostic signature for the prediction of patient outcomes and to investigate the value of MRG expression in the prognostic prediction of HCC. In our research, a Metabolism-Related Risk Score (MRRS) model was constructed using 14 MRGs (DLAT, SEPHS1, ACADS, UCK2, GOT2, ADH4, LDHA, ME1, TXNRD1, B4GALT2, AK2, PTDSS2, CSAD, and AMD1). The Kaplan-Meier curve confirmed that the MRRS has a high accuracy in predicting the prognosis of HCC patients (*p* < 0.001). According to the MRRS model, the area under the curve (AUC) values for predicting the prognosis of patients with hepatocellular carcinoma at 1, 3, and 5 years reached 0.829, 0.760, and 0.739, respectively. Functional analyses revealed that signaling pathways associated with the cell cycle were largely enriched by differential genes between high and low-risk groups. In addition, dendritic cells (DCs) (*p* < 0.001), CD4+ T cells (*p* < 0.01), CD8+ T cells (*p* < 0.001), B cells (*p* < 0.001), neutrophils (*p* < 0.001), macrophages (*p* < 0.001) had a higher proportion of infiltrates in high-risk populations. Low GOT2 expression is associated with poor prognosis in patients with hepatocellular carcinoma. Knockdown of GOT2 significantly increased the migration capacity of the Huh7 and MHCC97H hepatocellular carcinoma lines. Our research reveals that GOT2 is negatively related to the survival of patients with hepatocellular carcinoma and GOT2 may contribute to tumor progression by inhibiting the ability of tumor cells to migrate.

## Introduction

The incidence of liver cancer is increasing worldwide (Llovet et al., 2021; Villanueva, 2019). Liver cancer is the sixth most common malignancy and the third leading cause of cancer-related death globally. According to the latest statistics, there are 41,210 new cases of liver cancer and 29,380 deaths due to liver cancer in the United States in 2022 (Siegel et al., 2023). Hepatocellular carcinoma (HCC) patients account for more than 90% of liver cancer cases, and with the implementation of targeted and immunotherapy, the life expectancy of patients with hepatocellular carcinoma has increased (Llovet et al., 2018; Zucman-Rossi et al., 2015; Schulze et al., 2015). These treatments have a good therapeutic effect in patients with early-stage hepatocellular carcinoma. Due to chemotherapy tolerance and insensitivity to radiotherapy, the choice of effective treatments for patients with advanced hepatocellular carcinoma is seriously affected, resulting in poor prognosis (Llovet, Burroughs & Bruix, 2003). In the United States, the 5-year survival of patients with HCC is only 18% (Jemal et al., 2017). Traditional prognosis prediction systems for cancer patients, such as TNM staging, are increasingly difficult to cover the diversity of clinical features of HCC patients (Shao et al., 2016). The development of new prognostic models at multiple levels will help to better distinguish between different types of HCC patients (Shibata, 2021). Recent studies have demonstrated favorable outcomes in discriminating between HCC and predicting HCC prognosis, through the creation of multiple test-based indicators (Luo et al., 2022; Xie et al., 2022). This facilitates better extraction of features from different types of patients to facilitate more effective treatment of different types of patients.

Abnormal metabolism is one of the characteristics of HCC (Hanahan & Weinberg, 2011). During the malignant transformation of cells, metabolism usually undergoes drastic changes. Tumor cells regulate metabolism, promoting energy production and accumulation (Cheng et al., 2018). Hepatocellular carcinoma cells have elevated levels of metabolism to maintain their high metabolic levels for plasma membrane synthesis and energy production (Sangineto et al., 2020). Oncogenic signaling pathways, including B-Raf kinase (BRAF) and epidermal growth factor receptor (EGFR), drive dysregulation of fatty acid metabolism, affecting membrane composition and saturation to regulate tolerance to reactive oxygen species (ROS) and cancer cell survival (Talebi et al., 2018; Gimple et al., 2019; Bi et al., 2019). In addition, recent studies have shown that alterations in metabolism in tumor cells are able to promote tumor invasion and metastasis, regulate oxidative stress, and provide energy under various cellular stress situations (Pascual et al., 2017; Rohrig & Schulze, 2016; Ladanyi et al., 2018). Given that metabolism abnormalities play an important role in tumor progression, it has not been explored whether metabolism genes can be analyzed to build a prognostic model for HCC patients.

The role of metabolism in the regulation of immune cells has recently attracted widespread attention. Research evidence in several types of solid tumors shows the importance of metabolic reprogramming of immune cells in tumors, suggesting a new strategy for the treatment of HCC patients (Zhang et al., 2018). The function of immune cells in the HCC tumor microenvironment is closely related to abnormal metabolism (Hu et al., 2020). However, whether the expression level of MRGs in HCC patients affects immune cell infiltration in the tumor microenvironment remains to be explored.

In our research, we developed a novel prognostic prediction model based on MRGs, which is named “Metabolism-Related Risk Score” (MRRS), to predict the prognosis and survival of HCC patients. The immune-related components of different risk groups were analyzed, and the results showed that there were significant differences in the tumor immune microenvironment between the two groups. The evaluation of our predictive typing model shows great potential to guide the classification and treatment of HCC patients, demonstrating its clinical value and significance. Currently, a variety of genes and proteins that regulate metabolism have been identified, including GOT2, which has been reported to be involved in metabolism (Liu et al., 2022). However, the effect of this metabolism-related gene on the development and progression of hepatocellular carcinoma is unclear. Our study preliminarily explored the relationship between GOT2 and the prognosis of patients with hepatocellular carcinoma and preliminarily explored its effect on the migration ability of hepatocellular carcinoma.

## Methods

### Collection and processing of gene expression data and clinical information in HCC patients

**Figure 1:**
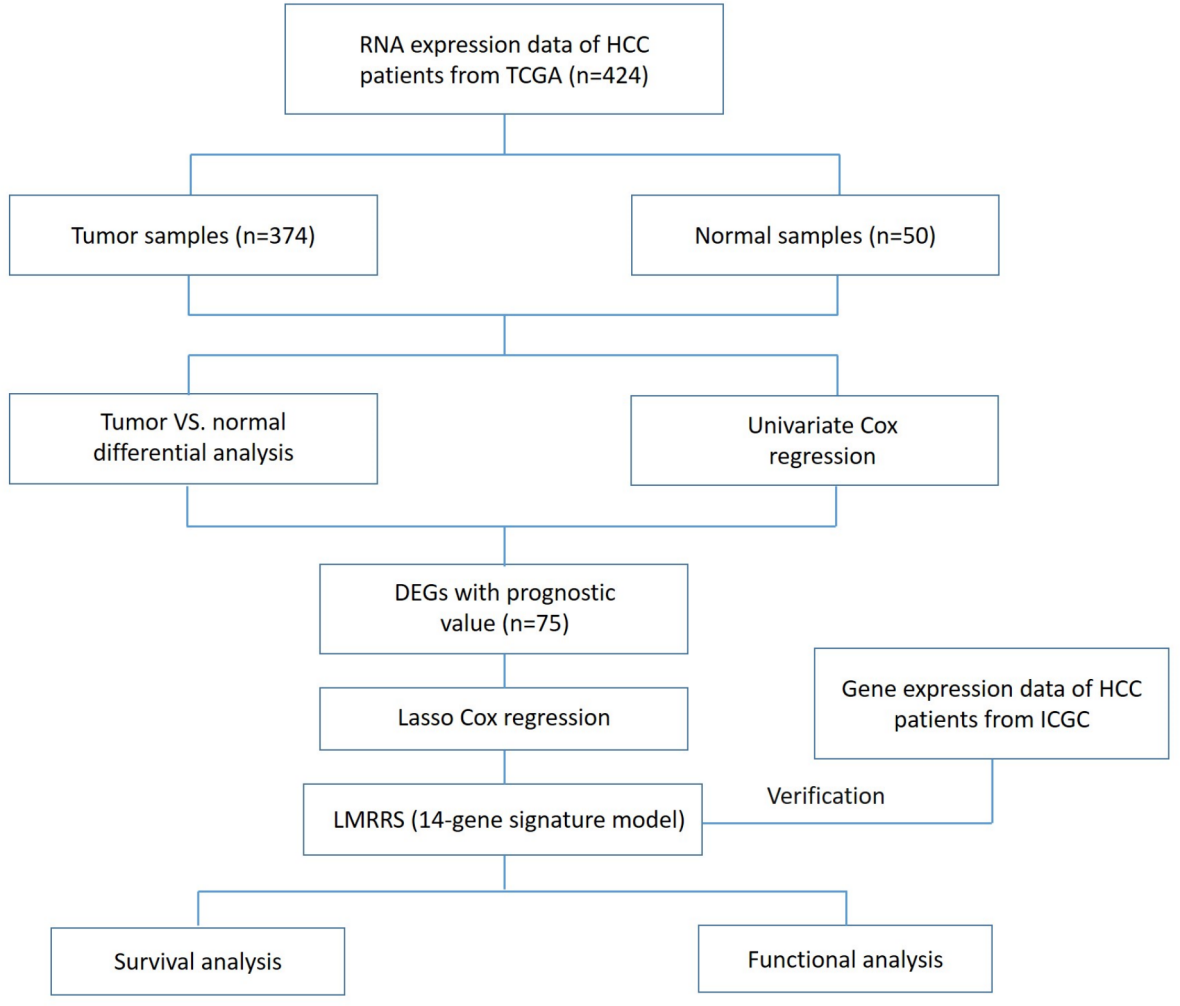
Flow chart of data collection and analysis in the present study.

Gene expression data and corresponding clinical information of 374 tumor tissues and 50 normal tissues of 374 patients with hepatocellular carcinoma as of March 03, 2022, were obtained from TCGA (https://portal.gdc.cancer.gov/repository). Of these, 44 patients were removed due to incomplete clinical data. Therefore, tissue gene expression data and complete follow-up information from the remaining patients (*n* = 330) were incorporated into our training dataset for further analysis. The test dataset used for validation was gene expression data and clinical information from an additional 231 tumor samples obtained from the ICGC portal (https://dcc.icgc.org/projects/LIRI-JP). These samples were mainly from Japan (Fujimoto et al., 2016). Since this data is all available online and comes with permission to use it, no additional ethical approval is required. The current study follows the TCGA and ICGC data access policies and publication guidelines. The flowchart of this research is shown in Fig. 1.

### Construction and validation of metabolism-related risk scores

Utilize the “limma” R package to determine the DEGs associated with metabolism between tumor and normal tissues, the false discovery rate (FDR) in the TCGA cohort < 0.05. Univariate Cox analysis of overall survival (OS) to screen for prognostically relevant MRGs, and visualize it with a forest plot. The intersection of metabolism-related DEGs with prognostic genes is demonstrated by a Venn diagram and visualized by a heatmap. An interactive network for generating prognostic DEGs from the Search Tool for the Retrieval of Interacting Genes (STRING) database (https://string-db.org). We use the LASSO Cox regression analysis to build a prognostic model that minimizes the risk of overfitting by performing the “glmnet” function of the R package. The penalty parameter (*λ*) of the model is determined by tenfold cross-validation following the minimum criterion (*i.e.,* the *λ* value corresponding to the lowest partial likelihood bias). Subsequently, the patient’s risk score is calculated based on gene expression and the corresponding Cox regression coefficient as follows: score = sum (expression of each gene × corresponding coefficient) (Table S1). Patients were then divided into high-risk and low-risk groups based on median risk score values. Based on the expression of gene signatures, PCA is performed using the “prcomp” function of the “stats” R package. T-SNE and PCA analysis using the prcomp function in the “Rtsne” package and the “stats” package to explore the distribution of high- and low-risk groups. Kaplan–Meier survival curve and time-dependent ROC curve analysis were applied to compare survival between the above two groups to assess the predictive accuracy of gene signatures.

### Functional enrichment analysis of HCC patients classified based on MRRS

Classification of HCC patients based on MRRS. Patients between high- and low-risk groups were analyzed for gene ontology (GO) and Kyoto Encyclopedia of Genes and Genomes (KEGG) pathways using the “clusterProfiler” R package in the high- and low-risk groups (29,31). The GO term and KEGG pathway with a *p*-value of <0.05 were statistically significant.

### Cell culture

The human hepatocellular carcinoma cell lines Huh7 and MHCC97H were purchased from the American Type Culture Collection (Rockville, MD, USA). Cell lines were all maintained at 5% CO_2_ at 37 °C and cultured in DMEM medium (Gibco, Waltham, MA, USA) supplemented with 10% and 13% FBS (Gibco, Waltham, MA, USA).

### Small interfering (siRNA) transfection

Tsingke biological technology offers GOT2 small interference RNA (siGOT2-1#, siGOT2-2#, siGOT2-3#) and non-target small interference RNA (siNC). The siGOT2 sequence was designed by Tsingke Biologics. Follow the manufacturer’s instructions for transfection in Opti-MEM medium (Gibco, Waltham, MA, USA) using RNAiMax Transfection Reagent (Invitrogen, Carlsbad, CA, USA). After stable transcription, collect cells for the next step of the experiment.

### RNA extraction and qRT-PCR

Total RNA was isolated and extracted from HEK293 cells using TRIzol reagent (Invitrogen, Carlsbad, CA, USA) and detected using a NanoDrop 2000 spectrophotometer. qRT-PCR was performed using SYBR-Green PCR kits (Takara, Shiga, Japan) and 7500 Fast Real-Time PCR System (Life Technologies, Carlsbad, CA, USA). The primers were synthesized by Tsingke biological technology. The expression level of the gene is compared with that of the housekeeping gene GAPDH. The following primers were used for RT-qPCR analysis: GAPDH, 5-ACAACTTTGGTATCGTGGAAAG-3; 5-GCCATCACGCCACAGTTTC-3 and GOT2,5′-AAGAGGGACACCATAGCAAAAAAA-3′; 5′-GCAGAACGTAAGGCTTTCCAT-3′. All experiments were performed using three complex wells.

### Scratch wound assay

5 × 10^5^ cells (three replicates per set) were seeded into a 6-well plate and incubated to reach confluence. Scratch the monolayer of cells using a tip and wash with serum-free medium to remove isolated cells. The cells are then cultured in complete medium containing 3% FBS. Huh7 and MHCC97H were shot after 0 h and 12 h. The wound closure area was calculated as follows: relative migration ratio (%) = (0 h wound area in experimental group - 12 h wound area in experimental group)/(0 h wound area in control group - 12 h wound area in control group) ×100.

### Statistical analysis

The Perl language is used for the data matrix and all data processing. Data analysis and visualization are performed in R (version 3.6.3) and the following packages are used for data analysis: “limma”, “survival”, “venn”, “pheatmap”, “igraph”, “reshape2”, “glmnet”, “survminer”, “Rtsne”, “ggplot2”, “clusterProfiler”, “org. Hs.eg.db” and “enrichplot”. The student *t*-test was used to identify MRGs that were differentially expressed between tumor tissues and normal tissues. When performing prognostic analysis of HCC patients, HCC patients in the training dataset were divided into two subgroups based on the optimal cut-off value for the marker determined by the “survminer” package in R. The ratio of high-risk patients to low-risk patients in the training dataset is then applied to the validation dataset. A two-tailed *p*-value < 0.05 was considered statistically significant (^*^p < 0.05, ^**^p < 0.01, ^***^p < 0.001).

## Result

### Identify differentially expressed genes in HCC tumor tissues

**Figure 2:**
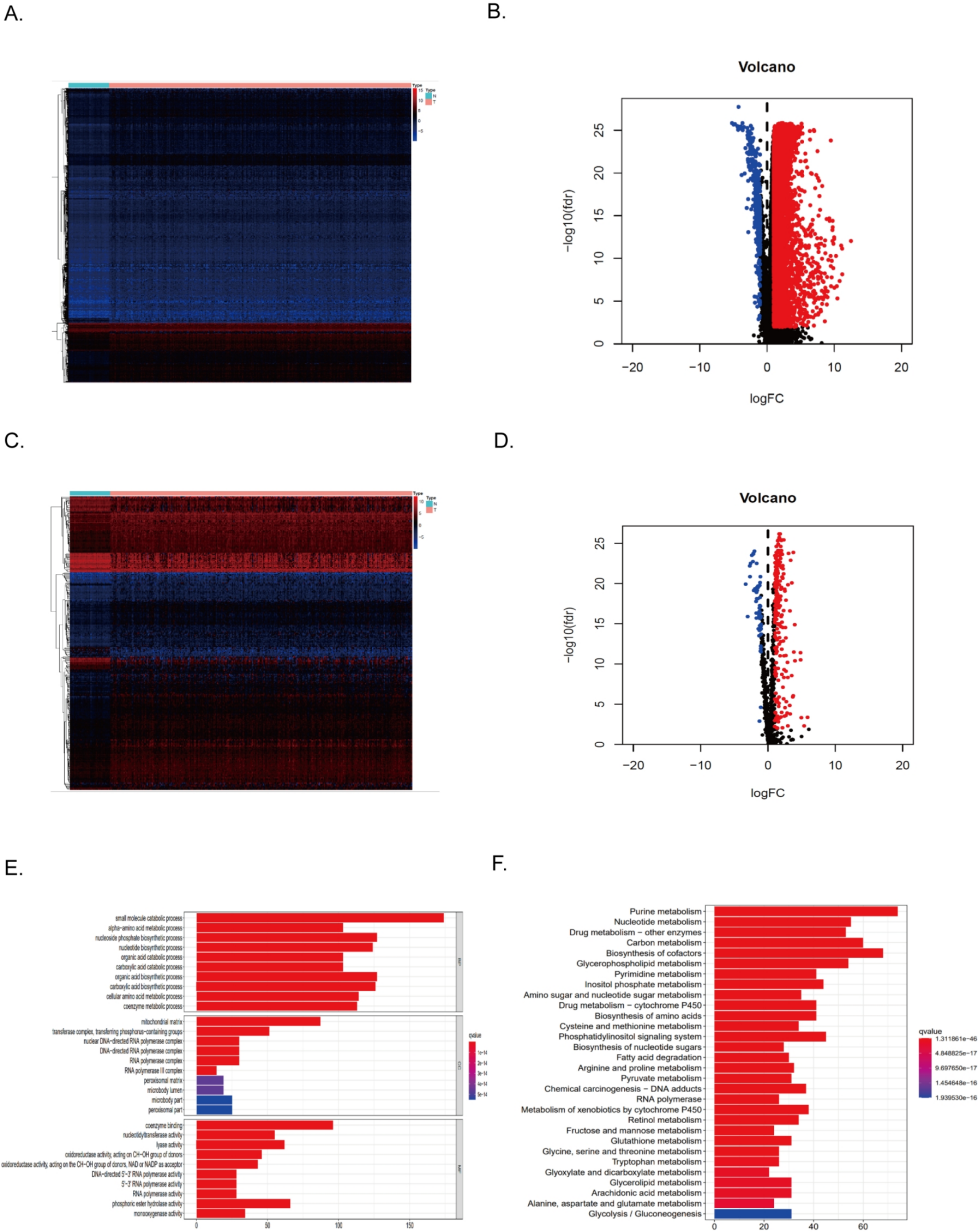
Differentially expressed MRGs in HCC patients. (A) Heatmap of DEGs in HCC. The color from blue to red represents the progression from low expression to high expression. (B) Volcano plot of differentially expressed genes in HCC. The red dots in the plot represent upregulated genes, and the blue dots represent downregulated genes with statistical significance. Black dots represent no differentially expressed genes in HCC. (C) Heatmap of differentially expressed MRGs in HCC. The color from blue to red represents the progression from low expression to high expression. (D) Volcano plot of differentially expressed MRGs in HCC.

374 HCC tissues and 50 normal tissues were obtained from TCGA. By comparing the expression levels of the genes, 7,498 DEGs were found (*P* < 0.01) (Table 1). Compared with normal tissues, most genes were unrestricted in tumor tissues, with 7,104 gene expressions upregulated and 394 gene expressions downregulated in HCC tissues (Figs. 2A–2B). Among them, there were 286 metabolism-related DEGs, 228 MRGs were up-regulated, and 58 MRGs were downregulated (Figs. 2C–2D). GO analysis showed that the small molecule catabolic process, *α*-amino acid metabolism process, and nucleoside phosphate biosynthesis process of HCC tumor tissue received significant changes (Fig. 2E). The results of KEGG enrichment analysis showed that the signaling pathways of purine metabolism, nucleotide metabolism, carbon metabolism, and biosynthesis of cofactors were significantly altered in tumor tissues of HCC patients (Fig. 2F). Collectively, these results suggested that the level of metabolism in tumor tissues in HCC patients is significantly different than in normal tissues.

**Table 1:** DEGs between HCC tissue and normal tissue. 374 HCC tissues and 50 normal tissues obtained from TCGA. By comparing the expression levels of the genes, 7,498 DEGs were found (*P* < 0.01).

Gene	conMean	treatMean	logFC	*p*Value	fdr
IL4I1	0.408676109	1.900420927	2.217289189	4.63E−08	8.22E−08
AKR1C1	27.5295606	64.19352837	1.221446277	0.000384398	0.000512757
PMM2	0.961974242	1.340717505	0.478935118	0.000139748	0.000194667
SMS	10.69932606	20.11237342	0.910563416	7.48E−17	2.80E−16
DGAT2	26.4610131	19.77988279	−0.419834421	2.00E−05	3.01E−05
IDO1	0.444306793	1.213901216	1.450022921	0.000360873	0.000483084
ACSL4	7.321489016	46.1219159	2.65524345	3.57E−12	9.03E−12
NAGK	2.34741402	3.529596543	0.588430966	4.54E−10	9.44E−10
GSTM4	3.97687114	6.010324011	0.595808951	0.003231848	0.00404651
ARSA	13.84392328	23.18197984	0.743750933	1.06E−09	2.14E−09
ACSL5	31.7718056	31.6335848	−0.006290026	0.000220461	0.000300448
ACSL1	181.0926394	77.88915995	−1.217233445	8.80E−18	3.80E−17
DUT	3.95256818	10.54330863	1.415465421	3.56E−24	5.49E−23
G6PD	1.311714874	13.58214647	3.372185434	6.03E−25	1.26E−23
PCK1	310.8995322	98.70077197	−1.655315172	1.33E−19	7.38E−19
CA13	0.64421103	0.599896967	−0.102818626	0.003296604	0.004120755
ME2	1.285344598	2.315627387	0.84924793	4.85E−09	9.30E−09
PDE3B	2.727241112	2.52907411	−0.108832939	0.033469155	0.038286685
PFKP	0.971925658	4.860376484	2.322150197	1.35E−06	2.23E−06
POLE2	0.325707436	1.639062106	2.331221958	5.74E−23	6.11E−22
NNT	17.79027552	16.39284085	−0.118022962	0.005707158	0.006949846
PAFAH1B1	5.69548084	6.833324791	0.262770054	0.016430951	0.019203356
GK	6.33197458	5.984056725	−0.081531613	0.009607045	0.011531509
ASPA	1.222286356	0.511516308	−1.256730176	1.83E−17	7.57E−17
AMDHD1	41.6883632	23.51702173	−0.825939365	1.15E−12	3.01E−12
ASNS	0.500573577	2.570302345	2.36028403	2.43E−09	4.77E−09
PDE6D	2.1416811	4.452477304	1.055864581	5.61E−23	6.05E−22
FMO5	45.5107514	42.94005501	−0.083883366	0.014548154	0.017082202
SRM	12.1914571	32.5068222	1.414871963	2.34E−23	2.76E−22
HCCS	5.76547254	8.626777767	0.581382935	3.21E−10	6.81E−10
POLR1B	2.287206532	3.072608206	0.425877173	0.000107178	0.000151534
ECI1	28.0101036	36.95316634	0.399750668	0.001444436	0.001845261
PIP5K1A	3.47425607	7.244620632	1.060206053	1.21E−14	3.84E−14
LYPLA1	8.63802894	13.72384588	0.667910775	2.84E−09	5.50E−09
CA4	0.042076729	0.569382669	3.758304113	5.34E−07	8.99E−07
ACY3	17.17975988	15.21388658	−0.175321118	0.001858912	0.002358787
FAH	31.8209192	23.57034487	−0.433002643	1.15E−08	2.12E−08
CYP2A7	31.69290318	25.23152162	−0.328932609	2.65E−11	6.26E−11
MTR	1.27380779	3.363203	1.400688261	2.77E−16	9.85E−16
NANS	4.36062666	7.892830021	0.856007202	7.18E−17	2.70E−16
LPCAT4	0.587157698	1.661035346	1.500262836	1.57E−10	3.47E−10
LPL	0.141580397	1.102972743	2.961703712	1.86E−21	1.42E−20
CYP2B6	126.8640398	29.24442074	−2.11704987	1.14E−20	7.23E−20
AOC3	3.922423082	3.742339892	−0.067804562	0.003389248	0.004229557
WARS2	1.72666976	3.099357597	0.843977038	2.58E−16	9.22E−16
AMPD3	0.254917141	0.543059142	1.091080935	7.02E−05	0.000101288
PCCA	10.22722354	8.319849703	−0.297785167	1.59E−06	2.60E−06
BLVRB	72.1631298	95.84324968	0.409414914	0.009920099	0.011850751
NT5E	12.01055636	10.74436329	−0.16072299	0.000622658	0.000813334
PSPH	1.983490216	9.025365702	2.185944109	3.94E−24	5.83E−23
PIK3C2B	0.775023298	2.386662492	1.622682978	2.26E−18	1.05E−17
LTA4H	4.31542466	7.329835003	0.764278188	1.35E−18	6.39E−18
UGT1A1	27.79325188	16.52163987	−0.750377754	8.92E−09	1.66E−08
CYP2C18	28.99648586	18.82865448	−0.622948161	2.50E−09	4.88E−09
ALDH4A1	101.0099032	72.05610849	−0.487304101	1.35E−08	2.47E−08
CYP2C9	287.583338	118.3820532	−1.280529708	2.02E−17	8.19E−17
SGMS1	3.20430224	4.119475713	0.362450503	0.006877689	0.008321563
IMPA2	7.37347282	9.371278293	0.345901581	0.018040114	0.021051447
GUK1	19.8499378	43.05143148	1.116926722	8.60E−22	7.38E−21
POLE4	7.38593728	10.66305657	0.52976813	0.000190569	0.000261125
DLAT	3.66031604	7.052317534	0.946129215	1.16E−10	2.61E−10
PDHA1	13.28151162	19.02741134	0.518659943	7.54E−11	1.70E−10
CYB5R3	30.237305	43.89137451	0.53760789	2.01E−07	3.47E−07
AGPAT2	87.005614	74.04673086	−0.232672451	3.76E−05	5.54E−05
OXCT1	0.275234787	1.493923329	2.440371378	0.000229146	0.000311721
CDIPT	20.9301402	32.18929631	0.620999063	1.08E−11	2.66E−11
CA5A	2.3876375	2.073194093	−0.20372863	0.006409608	0.007780152
PLA2G12B	27.9585252	35.56349409	0.347108814	0.0050749	0.006189902
CHST11	0.731928728	1.923006546	1.393588597	0.008023001	0.009645487
POLR2G	8.5867008	20.36548606	1.245950419	3.97E−26	1.43E−24
SORD	41.8884452	30.0139334	−0.480919936	2.22E−07	3.83E−07
UROS	3.93219928	6.103738102	0.634356623	8.59E−11	1.94E−10
HAL	26.70119602	23.56047414	−0.180535793	0.001882257	0.002384403
UPRT	1.800134228	2.776221087	0.625017977	1.73E−11	4.18E−11
ENTPD6	4.89225452	13.77275387	1.493245689	7.12E−25	1.41E−23
SPHK1	0.741709572	5.347719945	2.849997622	8.75E−05	0.000124871
CANT1	4.13244666	10.66555898	1.367891476	6.59E−25	1.34E−23
ACADL	4.060608256	2.250819133	−0.851245719	6.55E−12	1.63E−11
MIF	18.5365111	45.69842886	1.301774837	2.78E−13	7.93E−13
UCK1	8.54689772	14.03707748	0.715769834	3.83E−13	1.08E−12
GALT	12.9575935	16.26293456	0.327789803	0.004091075	0.005046996
ACSM1	3.973052599	15.02401635	1.91895074	0.000302397	0.000406969
ALDH1A3	0.565172746	0.487798622	−0.212406216	8.54E−06	1.33E−05
POLR2K	10.2636964	29.85350878	1.540350105	2.97E−28	3.93E−26
PCK2	145.7806962	90.77617087	−0.683414157	6.45E−11	1.47E−10
LPCAT1	1.635662076	6.94731366	2.086580512	1.78E−16	6.50E−16
NAA80	1.86636393	4.187413568	1.165829082	1.23E−18	5.83E−18
ADCY9	2.877553656	4.347904755	0.595477507	3.01E−06	4.85E−06
GMPPA	5.56356326	11.56588251	1.055794274	1.45E−24	2.61E−23
CRLS1	14.10303008	21.11234898	0.58208194	0.000158942	0.000219782
SEPHS1	7.92983666	13.77461218	0.796648647	1.75E−20	1.06E−19
CYP2C19	3.085339415	0.382142328	−3.013247236	3.19E−17	1.25E−16
KDSR	7.4475128	6.084439599	−0.291634307	6.26E−05	9.06E−05
PFKFB2	0.382136054	1.420710904	1.89445473	6.30E−20	3.60E−19
POLR3H	2.7771828	4.797098075	0.788539794	1.59E−16	5.83E−16
CYP4F3	37.5404	30.65832969	−0.292164924	1.27E−05	1.94E−05
UXS1	1.5551954	4.725752008	1.603448067	1.67E−26	7.87E−25
POLA2	0.664501946	2.587714342	1.961333037	8.15E−26	2.28E−24
NT5C	5.03591866	11.91640459	1.242622127	8.44E−19	4.19E−18
BDH1	21.2007566	16.85562386	−0.330885726	0.000126492	0.000176528
LIPC	26.95726792	20.48967436	−0.395777233	3.01E−06	4.85E−06
PLA2G5	1.153261896	0.89094941	−0.372304753	0.000109361	0.000153757
PLA2G6	0.58048134	2.296062017	1.983840014	5.46E−24	7.63E−23
NMNAT1	1.26411244	1.805223587	0.514052741	9.85E−06	1.52E−05
ACO2	12.52959346	20.83091178	0.733386383	3.46E−08	6.20E−08
FTH1	139.0992968	286.4863544	1.042351297	8.93E−17	3.30E−16
ACADS	72.100922	32.05868751	−1.169302346	9.35E−22	7.93E−21
GGT5	7.32869966	4.290134866	−0.772534241	1.15E−12	3.01E−12
ALDH1A1	267.4815334	396.7508063	0.568793862	0.021467023	0.024858286
TPMT	20.16445966	18.52541508	−0.122308879	0.000533541	0.000700562
PDE5A	0.195980374	0.503871718	1.362347291	1.58E−06	2.59E−06
PRIM1	0.936742084	3.706683544	1.984405165	4.14E−21	2.89E−20
HIBCH	7.9563316	6.281329144	−0.341033536	5.63E−06	8.82E−06
RDH10	9.06576346	15.41329115	0.765674524	1.50E−06	2.46E−06
HEMK1	0.65588727	1.173926415	0.8398222	7.25E−21	4.89E−20
LPGAT1	8.05441224	21.31594928	1.404082087	2.17E−17	8.77E−17
TYMS	1.561858024	8.89844653	2.51029018	1.44E−20	8.89E−20
DNMT1	1.168587338	4.380206253	1.906233242	7.50E−21	4.97E−20
PC	48.5013028	37.34714447	−0.37702556	4.28E−06	6.78E−06
POLR1A	0.958182464	2.696126111	1.492515664	3.87E−26	1.43E−24
CAT	172.3659592	98.46953686	−0.807725505	2.28E−14	6.99E−14
CP	186.4100454	97.23571025	−0.938921455	7.95E−13	2.13E−12
DBH	10.78850207	1.804720176	−2.5796475	1.78E−24	3.10E−23
UGT2B10	198.5524482	92.80896422	−1.097184086	9.18E−13	2.45E−12
GNPDA2	0.319322526	0.662430617	1.052755026	1.31E−10	2.91E−10
NME4	16.31808562	24.98593807	0.614644568	0.000109914	0.000154247
MLYCD	2.48341314	1.547413766	−0.682465274	8.55E−15	2.74E−14
PFKFB1	8.5895856	7.825367716	−0.134429985	0.002113498	0.002672849
SULT1A2	9.07027648	6.751247492	−0.42599242	1.14E−05	1.75E−05
GALK1	24.967336	42.45332342	0.765835604	9.54E−05	0.000135645
PHGDH	23.51638218	13.43361449	−0.807818596	2.77E−11	6.48E−11
GPAT2	0.090898479	0.478071167	2.394897342	2.26E−10	4.91E−10
OPLAH	8.82522278	16.89672561	0.937039094	2.09E−10	4.56E−10
IMPDH1	1.350320208	4.233778779	1.648644327	7.85E−07	1.31E−06
NPR2	2.597858634	5.96065675	1.19814837	0.000471399	0.000621127
FMO3	137.9187216	93.88484103	−0.554854168	3.50E−08	6.27E−08
AACS	0.31328518	1.03790029	1.728119422	1.45E−19	7.96E−19
NMRK1	6.37048312	5.351685995	−0.251409317	8.27E−05	0.00011827
ENTPD5	22.40752186	18.09005981	−0.308785926	0.000475759	0.000625781
MPST	34.8617012	45.54922933	0.385783672	0.003609969	0.004497569
MTMR1	1.628000482	3.071386948	0.915789155	1.92E−14	5.94E−14
GLA	3.24193056	11.36254672	1.809361132	1.20E−24	2.21E−23
MGST3	5.56648038	10.14191067	0.865492151	1.91E−17	7.85E−17
EPHX1	524.41677	1035.59981	0.981680879	7.88E−06	1.23E−05
LYPLA2	21.932233	34.59496813	0.657509506	4.98E−13	1.38E−12
CAD	1.068638102	4.256222464	1.993800198	5.58E−26	1.76E−24
NEU3	0.572362622	1.294232685	1.177095651	1.28E−17	5.36E−17
EHHADH	68.1731674	41.91216309	−0.701835033	2.39E−10	5.18E−10
CTPS2	1.630246474	3.255587356	0.997827751	8.08E−18	3.53E−17
MTHFD2	0.687717227	1.248735294	0.860580299	0.006481558	0.007854858
SMPD3	0.70683542	0.332301761	−1.088880396	4.31E−17	1.65E−16
RDH16	121.453585	43.35288581	−1.48620514	1.45E−19	7.96E−19
DGKZ	1.274013736	3.212991644	1.334536397	1.11E−23	1.47E−22
TPI1	83.9320608	139.4822363	0.73278749	5.71E−14	1.69E−13
LCMT2	0.797014992	1.12892053	0.502265164	4.15E−07	7.04E−07
TAT	265.3094254	154.2425759	−0.782474879	2.12E−09	4.19E−09
HEXA	3.18901968	6.698987527	1.070830065	1.23E−18	5.83E−18
ARG1	240.2322	166.3619414	−0.530104112	1.68E−08	3.06E−08
CPT1C	0.112497877	0.356503529	1.664018582	1.18E−13	3.43E−13
MTHFD2L	1.241255578	0.761231939	−0.705392202	5.19E−10	1.08E−09
MTAP	1.15203675	1.583067801	0.458536306	2.94E−06	4.75E−06
RDH5	4.71202044	2.420467525	−0.961060059	6.15E−15	1.98E−14
DNMT3L	0.424289348	0.291648168	−0.540819449	2.06E−05	3.08E−05
HPRT1	14.17009836	18.06220948	0.35012461	0.009675838	0.011577274
ASS1	584.625332	252.8417532	−1.209277619	1.11E−18	5.38E−18
ACHE	0.686224915	1.858858895	1.437663848	0.032860592	0.037762172
BUD23	4.074106	8.36906785	1.038583427	1.30E−25	3.27E−24
GMPPB	1.87040842	4.208787714	1.170051414	8.29E−23	8.57E−22
PLCD4	0.125425913	0.438557777	1.805931481	1.23E−18	5.83E−18
PTDSS1	6.74127936	12.91530242	0.937987108	5.06E−15	1.64E−14
CS	5.37878536	13.8183489	1.361232919	1.45E−22	1.42E−21
PIKFYVE	1.39647462	1.870835031	0.421892995	0.004941202	0.006046366
POLD3	0.842582454	2.064203063	1.292695122	2.33E−19	1.24E−18
AOX1	221.5975442	121.9637437	−0.861489553	1.55E−11	3.77E−11
HMGCS2	664.916598	435.8643833	−0.609294073	5.83E−10	1.21E−09
FPGT	1.601297378	2.672968881	0.739201785	9.43E−13	2.51E−12
POLR2A	8.00200448	10.08390693	0.333621366	0.026768153	0.030902072
SGPP1	6.7100943	6.026959952	−0.154902563	0.004352827	0.005352418
POLD2	19.6641202	29.83523623	0.60145156	7.12E−09	1.33E−08
DGAT1	11.44228732	19.98911916	0.804839423	2.18E−09	4.30E−09
PNP	9.04160578	7.688707168	−0.233837983	0.000414134	0.000550477
ACADSB	72.1887034	32.77565001	−1.139148699	1.46E−18	6.86E−18
UGT2B11	0.932454448	13.38406332	3.843339116	1.32E−12	3.46E−12
HK3	1.755092942	1.065993519	−0.719348764	1.59E−10	3.50E−10
UCK2	0.97723134	4.97742167	2.348626575	2.02E−28	3.82E−26
NPL	2.542158828	4.829610362	0.925852632	0.012594123	0.014833951
B4GALT6	0.297784155	0.661726029	1.151967038	2.51E−07	4.30E−07
PLCB3	1.634257378	3.846683794	1.234980031	1.86E−21	1.42E−20
DLD	11.88321106	14.92935626	0.329227228	0.002449057	0.003086875
POLR1E	7.44986644	6.602367032	−0.17423122	0.003118202	0.003917209
POLR3K	2.48922156	5.518207323	1.148505014	2.44E−21	1.81E−20
OGDHL	26.5042672	14.50832449	−0.869343735	4.39E−13	1.23E−12
ALAS1	159.56769	100.1414548	−0.672129238	1.81E−09	3.59E−09
POLR3D	0.963096128	1.733721133	0.848120154	5.64E−08	9.99E−08
INMT	9.65272184	2.507403196	−1.944741802	7.33E−21	4.90E−20
GUSB	30.392177	38.53529635	0.342480467	0.002655896	0.003342003
CPS1	205.3781916	136.0158919	−0.594507771	2.45E−07	4.19E−07
METTL6	0.430309778	1.185576642	1.462141401	6.71E−28	5.07E−26
GOT2	87.1599586	65.06480682	−0.421788102	2.46E−09	4.82E−09
PTGES	0.297396069	3.200945654	3.428040699	0.014797686	0.017348219
AGPS	3.7115833	5.295453451	0.51271948	2.80E−05	4.14E−05
POLR1D	4.90762592	8.660998203	0.819508024	2.72E−18	1.25E−17
ACSL3	6.99338192	11.86570804	0.76273599	6.24E−09	1.17E−08
DHRS4	12.25678654	11.53249242	−0.087876441	0.020310735	0.023555461
HYI	3.01594442	5.084056814	0.753370311	2.55E−11	6.04E−11
GLUD1	180.0245172	133.4265423	−0.43214771	8.27E−10	1.69E−09
POLD4	8.05781742	12.54161247	0.638261825	5.34E−09	1.01E−08
PPOX	1.196744276	4.192724463	1.808773117	1.44E−29	6.98E−27
ADA	0.943392724	2.67245149	1.502233382	1.19E−17	5.01E−17
HMOX1	45.83893488	27.73183896	−0.725030234	9.52E−09	1.77E−08
DMGDH	31.7591144	15.47932348	−1.036828263	5.46E−16	1.88E−15
AHCY	44.0853498	69.89104425	0.664808295	2.94E−08	5.30E−08
PRIM2	0.502543684	1.859031108	1.887229998	4.42E−27	2.57E−25
ACADM	31.879362	20.11711322	−0.664199462	2.07E−11	4.96E−11
PIK3CB	1.966956368	3.395752372	0.787766302	5.26E−13	1.44E−12
PRUNE1	3.4628476	9.611363235	1.472782175	5.02E−23	5.49E−22
CHKB	0.422492212	1.182076973	1.484327329	9.12E−19	4.50E−18
MGLL	13.76947858	12.46297017	−0.143825997	0.009675841	0.011577274
OCRL	1.958109446	5.65422446	1.529867751	1.85E−24	3.10E−23
LDHD	55.065139	32.56811545	−0.757679016	6.24E−13	1.70E−12
HMBS	2.84492594	6.077665142	1.095126093	4.39E−23	4.87E−22
DPYD	9.11150098	7.318402323	−0.316160007	6.13E−05	8.89E−05
ABAT	53.955323	26.73360559	−1.013110868	3.57E−16	1.26E−15
ADH6	89.808434	46.97759442	−0.934878097	6.33E−14	1.86E−13
PDE7B	0.930007826	0.456690251	−1.026026862	1.31E−15	4.37E−15
PISD	2.83145052	4.389637725	0.632560561	7.78E−10	1.60E−09
PIP5K1C	1.467138022	3.719134168	1.341962194	3.88E−23	4.44E−22
ALDH3A1	0.752936723	44.07999412	5.871451597	0.000343499	0.000460643
GBA	5.56522966	22.70087955	2.028235066	3.64E−28	3.93E−26
GALK2	1.588663302	2.448799751	0.624261408	4.17E−08	7.43E−08
MAOB	92.0239682	75.53398313	−0.284883802	2.02E−06	3.29E−06
ACAT1	82.9427028	47.2412729	−0.812067222	2.12E−16	7.66E−16
UGDH	22.73636822	41.03832833	0.85197014	0.000206002	0.00028176
EPHX2	46.3273598	24.00327051	−0.948633475	3.57E−17	1.37E−16
CERK	5.53242282	11.22309283	1.02048698	1.28E−14	4.03E−14
TAZ	1.723546396	5.52082373	1.679503404	7.52E−29	1.89E−26
PGD	20.44094368	39.89972749	0.964917092	3.83E−09	7.38E−09
ACO1	20.96810206	19.6283929	−0.095254226	0.00010772	0.000151732
AMDHD2	2.031742526	4.730317108	1.219219314	1.28E−20	7.99E−20
PDE6G	0.86451153	0.639854258	−0.434141871	5.70E−05	8.28E−05
CYP4A22	60.5277696	19.74899941	−1.61581763	1.17E−20	7.34E−20
SDS	194.0116684	163.218774	−0.249336412	4.38E−06	6.93E−06
FMO4	10.20612596	9.414288939	−0.116511315	0.003193554	0.004005204
GPAT3	5.552175014	4.162315257	−0.415666805	0.012249465	0.014473155
PFKL	12.61516374	19.37322019	0.618904845	2.04E−10	4.46E−10
HAO1	172.769988	92.22580563	−0.905610236	3.35E−17	1.30E−16
CYP4F2	60.3235252	29.46836538	−1.033553704	6.21E−15	1.99E−14
FTCD	155.5260496	86.5196214	−0.846056985	6.71E−13	1.81E−12
HPD	605.159282	532.9277054	−0.183375083	0.000107178	0.000151534
PDE4B	1.062459026	0.963981326	−0.1403301	0.0009672	0.00125686
PRDX6	171.8414344	221.4824048	0.36611415	0.001355076	0.001736982
SUCLG1	21.1592364	27.17118061	0.360789689	0.000143973	0.000200183
PIK3C2G	2.247678282	1.473184304	−0.609497621	2.52E−08	4.56E−08
PGM3	4.06042848	5.511269867	0.440752795	0.00140208	0.001794187
GPAT4	5.23151944	9.836442457	0.910906608	1.77E−12	4.55E−12
CHDH	7.9646379	11.15826518	0.486432065	9.46E−06	1.46E−05
BLVRA	3.276705816	12.07960422	1.882255129	5.93E−14	1.75E−13
MARS2	1.96582365	2.821305799	0.52122914	4.85E−05	7.09E−05
PSAT1	58.4287532	37.24670641	−0.649565645	3.01E−10	6.41E−10
ALDH5A1	25.002095	21.66769979	−0.20650298	0.00010772	0.000151732
GGT7	3.07695892	5.573133026	0.856983407	2.99E−11	6.94E−11
AGPAT4	0.136465682	0.57271225	2.069272273	9.51E−10	1.93E−09
ALDH9A1	48.6317048	40.3139759	−0.270617093	2.24E−06	3.64E−06
PI4KB	4.0879725	11.41793196	1.481843974	1.67E−26	7.87E−25
GALE	10.83432624	18.55155752	0.775930877	2.02E−11	4.85E−11
PLD1	2.33434292	2.279957658	−0.03400948	0.026683904	0.030851987
CYP2U1	1.250805474	1.14419921	−0.128519184	0.012594123	0.014833951
ENOPH1	5.50486998	11.72488047	1.090792821	2.78E−21	2.03E−20
AGXT	394.753526	250.6042388	−0.655541335	4.40E−10	9.18E−10
PLCB2	1.160730889	1.104038541	−0.072242991	0.040462466	0.046007774
CYP1B1	1.337915968	5.94969667	2.152828612	0.006928705	0.008369875
NT5C2	1.540448554	2.299890391	0.578214605	0.001724337	0.002195404
NME6	1.050427416	2.501002343	1.251529933	3.81E−27	2.40E−25
HMGCL	40.4827466	24.59046419	−0.719208206	2.52E−16	9.07E−16
SAT1	121.6407268	113.7395081	−0.096892871	0.033469155	0.038286685
SUCLG2	54.220537	39.39971703	−0.460654133	3.94E−10	8.24E−10
ADH1A	388.209718	164.8505374	−1.235677643	5.65E−17	2.13E−16
CTH	37.1185504	29.77105419	−0.318230061	1.40E−08	2.56E−08
CYP3A4	765.778506	344.3796666	−1.152927204	1.69E−15	5.61E−15
ECHS1	468.98201	301.2351756	−0.638642337	5.40E−13	1.48E−12
PGM1	59.3355864	38.43717944	−0.626395144	1.11E−13	3.24E−13
POLD1	1.184827396	4.730074491	1.997185999	1.56E−26	7.87E−25
NAMPT	30.29449552	16.41230958	−0.884277408	2.28E−07	3.92E−07
PFKM	0.866943006	2.25856606	1.381398054	3.07E−06	4.93E−06
SCLY	0.20643543	0.46320077	1.165947056	1.86E−14	5.79E−14
CHKA	3.36662532	11.5937026	1.78396631	5.27E−22	4.74E−21
GDA	6.26289082	5.902956593	−0.085390996	0.003981116	0.004927447
GPX2	106.9847132	267.7036539	1.323232162	0.010097916	0.012044118
PAPSS2	20.1801266	15.74261221	−0.358260274	1.30E−05	1.98E−05
PLCG1	1.260780372	3.995510695	1.664062936	1.77E−21	1.39E−20
PIP4K2C	4.43018188	8.973248829	1.018264488	9.69E−18	4.13E−17
EARS2	3.1501315	5.085392201	0.690946989	1.33E−11	3.27E−11
GSS	17.1787072	29.50461679	0.780319251	4.06E−18	1.85E−17
AK3	31.4693146	24.77009647	−0.34534627	3.86E−08	6.90E−08
APRT	28.6023808	54.41106794	0.927764906	3.86E−14	1.16E−13
FMO1	0.209436746	1.621167947	2.952447066	0.003981116	0.004927447
DHRS4L2	11.34542994	10.55728929	−0.103871829	0.00698006	0.008405017
AKR1C3	17.68298576	83.41396337	2.237927015	1.18E−23	1.54E−22
GNPAT	8.1486725	23.37881262	1.520564705	1.98E−27	1.36E−25
UROD	21.544076	30.24876272	0.489584908	5.04E−09	9.60E−09
CBR1	58.780441	99.21114632	0.755166033	0.001746125	0.002219401
SMPD4	2.28095394	5.437838743	1.253396055	2.14E−22	2.02E−21
GPX7	1.50203363	4.344910673	1.532409402	0.000302397	0.000406969
GSTO2	0.303990672	0.880769726	1.534737826	0.001201774	0.001545723
GSTK1	45.1586356	63.78578441	0.498233036	8.94E−08	1.57E−07
CMAS	15.22241776	20.10836615	0.401598346	5.00E−05	7.30E−05
PGP	2.46275828	8.344557131	1.76056045	1.37E−25	3.34E−24
LRAT	1.094300194	0.233448683	−2.228831208	9.54E−25	1.80E−23
UCKL1	4.9052273	11.12656123	1.181615888	1.80E−23	2.23E−22
PIP4K2B	3.30158812	7.811014925	1.242349865	1.56E−23	1.96E−22
ACOT12	21.45356354	13.71772584	−0.645175979	9.38E−09	1.75E−08
GSR	15.11395882	25.70655358	0.766254607	4.11E−05	6.04E−05
CPT2	25.1462894	16.46242324	−0.611168817	2.48E−12	6.29E−12
PLD2	1.824123198	3.033992898	0.734014539	8.11E−09	1.52E−08
POLR3GL	12.67931058	19.32978751	0.608349475	1.82E−09	3.61E−09
GGCT	7.3906693	16.97966798	1.200031323	1.92E−23	2.34E−22
CYB5R1	6.89941468	17.42151749	1.336324415	1.52E−25	3.59E−24
PDE2A	2.178131344	1.126729193	−0.950950145	1.15E−13	3.35E−13
OGDH	13.79518708	24.98405926	0.856842875	4.59E−13	1.28E−12
ENPP1	9.1646637	7.547183961	−0.280143502	1.15E−05	1.76E−05
COMT	30.706629	25.72272779	−0.255506498	2.65E−05	3.94E−05
QPRT	43.8780206	54.08350685	0.301690256	0.04267486	0.048305126
AGMAT	32.4994148	25.32091879	−0.360083985	2.71E−05	4.02E−05
GRHPR	62.909347	40.6577625	−0.629743565	9.98E−12	2.48E−11
ACER2	0.36464292	0.323416101	−0.173092878	0.012247144	0.014473155
FLAD1	4.34967458	13.71639311	1.656921782	1.85E−29	6.98E−27
PYCR3	2.93010406	9.072809425	1.630597454	9.42E−25	1.80E−23
HEXB	14.85885542	30.48633278	1.036839629	3.86E−21	2.72E−20
NAT1	2.219190636	1.399346689	−0.66528017	2.09E−11	4.98E−11
GART	3.11647116	5.301358952	0.766448866	3.40E−14	1.03E−13
PLCE1	0.212974455	0.608776786	1.515232948	9.85E−19	4.83E−18
PLCD3	0.266926144	1.228215767	2.202051505	8.51E−16	2.88E−15
CYP3A43	2.620158394	1.416590761	−0.887230991	1.35E−12	3.50E−12
ETNK2	33.401069	32.2062868	−0.052551941	0.004773988	0.005851236
CDS2	2.7498919	4.632668795	0.752468636	1.26E−14	3.97E−14
NME2	6.86305604	19.23153189	1.486550645	1.71E−21	1.36E−20
IMPDH2	12.91924434	36.52956009	1.499542692	3.89E−24	5.83E−23
OTC	88.002804	58.70658403	−0.58402718	6.74E−07	1.13E−06
PIK3C2A	2.259323538	3.277646011	0.536769169	3.36E−05	4.96E−05
CYP4A11	233.0618168	72.59752175	−1.682720458	1.82E−24	3.10E−23
ACSS1	0.750883248	2.273225868	1.598080527	1.27E−05	1.94E−05
POLE	1.3055207	2.220828061	0.766472369	6.30E−10	1.30E−09
ACP1	11.20816652	18.54387434	0.7263924	8.05E−21	5.19E−20
ACMSD	26.174961	21.07948543	−0.312347738	4.05E−05	5.96E−05
CYP2A6	536.9454174	278.4392503	−0.947412844	2.29E−11	5.43E−11
SHMT1	52.7147148	32.16605069	−0.712666918	1.45E−12	3.75E−12
CEL	0.033783259	0.531241933	3.974988605	3.70E−16	1.30E−15
MIOX	0.025307544	0.730525513	4.851295268	1.25E−11	3.07E−11
PLA2G7	1.73344392	4.023914912	1.214958635	0.000584406	0.00076469
GNPNAT1	13.14352562	9.498633602	−0.468560417	5.97E−09	1.12E−08
PNPT1	2.55535368	4.267066066	0.739721463	3.15E−14	9.59E−14
INPP5K	3.03390274	4.147665679	0.451124774	4.84E−07	8.16E−07
ADK	19.8979868	13.99084463	−0.508139411	1.05E−11	2.60E−11
CA12	0.325978387	3.7360177	3.518653069	1.48E−09	2.96E−09
NMNAT3	0.695570616	1.378986438	0.987339375	1.16E−09	2.33E−09
KMO	8.11238004	3.338421404	−1.280959164	8.62E−18	3.74E−17
ARG2	0.49592443	2.010793536	2.019572755	7.62E−05	0.000109157
GNPDA1	2.73301806	7.292774088	1.41597271	3.86E−20	2.26E−19
MAT2A	11.17323804	17.09991339	0.613941674	0.000105036	0.000149064
AGK	1.495999866	2.767032531	0.887229559	4.65E−16	1.62E−15
UROC1	40.84584802	11.96254955	−1.771664535	3.22E−19	1.67E−18
ADH4	804.750736	228.0260374	−1.819343421	8.53E−21	5.46E−20
UGT2B7	279.779916	115.0979264	−1.281430561	6.96E−18	3.07E−17
PAH	116.2219842	94.99517702	−0.290956817	0.000150537	0.000208925
ALOX15B	0.142066661	1.817887709	3.677623143	7.72E−07	1.29E−06
GMPR2	7.85884262	12.66631626	0.688608242	1.09E−18	5.30E−18
GAA	20.41744244	34.96541419	0.776126435	1.55E−11	3.77E−11
APIP	1.640616216	3.661128344	1.158050556	3.30E−22	3.00E−21
PFAS	1.153738786	2.725413061	1.240158275	5.61E−19	2.82E−18
IMPA1	4.43702154	7.935114115	0.838659414	2.13E−12	5.44E−12
NAT2	25.52141146	4.833929648	−2.400439738	1.20E−25	3.13E−24
SMPD2	1.62738852	3.829329681	1.234533154	1.20E−19	6.72E−19
SUCLG2P2	0.361432093	0.260115564	−0.474571886	7.34E−05	0.000105697
SYNJ1	0.648474406	0.91861337	0.502408146	0.000217285	0.000296654
PAICS	11.60426494	16.32540539	0.492463677	1.22E−08	2.24E−08
TK1	2.468539172	20.42635341	3.048702228	5.30E−26	1.74E−24
AFMID	18.4721284	24.5251126	0.408909654	0.000233607	0.000317219
ALAD	38.4233746	37.10296135	−0.050449893	0.01920916	0.022381043
NT5C3A	1.954837404	4.189451043	1.099712601	2.24E−17	9.01E−17
GSTZ1	11.81957816	4.287539533	−1.462956668	2.84E−21	2.06E−20
PGM2L1	0.22568328	0.446572794	0.984595823	0.001355076	0.001736982
DAO	17.87964038	12.9572827	−0.464554521	2.75E−06	4.46E−06
POLR2J	16.07014268	32.76108605	1.027600446	2.75E−19	1.45E−18
FHIT	0.791113118	2.26275842	1.516126666	6.30E−22	5.60E−21
GPD1	36.7540008	17.69852929	−1.05427182	4.63E−13	1.29E−12
CHST12	0.378759634	0.713920394	0.91448063	2.32E−13	6.63E−13
HMGCS1	26.26538034	41.55101209	0.661721142	0.000113855	0.000159481
BHMT	188.5596978	89.71365222	−1.071621902	2.19E−13	6.30E−13
UGT1A4	30.3252135	24.22035055	−0.324298057	6.85E−08	1.21E−07
LAP3	38.7008652	30.80759113	−0.329079938	2.01E−05	3.02E−05
ACP4	0.013881318	0.395615568	4.83288278	1.58E−12	4.10E−12
PDE11A	0.6499311	0.489365997	−0.409372922	4.45E−07	7.54E−07
POLA1	0.530661838	1.544546194	1.541318312	4.30E−19	2.19E−18
ALDH2	154.9258366	71.34767851	−1.118639365	1.47E−22	1.42E−21
CES5A	1.156581576	0.643541115	−0.845762796	2.67E−09	5.18E−09
XDH	15.64604762	9.530809199	−0.715127647	1.12E−09	2.25E−09
JMJD7-PLA2G4B	0.08957713	0.24466282	1.449592525	1.59E−10	3.50E−10
HK1	1.756529668	3.532845207	1.008102594	0.004998106	0.006106101
POLR3A	1.148311722	2.372790232	1.047070233	7.34E−23	7.69E−22
ACSM3	8.26916818	3.080383675	−1.424632156	3.51E−21	2.50E−20
ZNRD1	1.920201268	4.468929856	1.218671864	4.96E−20	2.88E−19
GFPT1	4.77169852	8.360365723	0.809063159	2.23E−11	5.31E−11
LCLAT1	1.079431836	1.825327458	0.757883159	4.13E−11	9.55E−11
SULT1E1	4.766748444	3.612816882	−0.399881359	7.85E−08	1.38E−07
LIPG	4.0745132	2.953168638	−0.464363962	7.83E−06	1.22E−05
MBOAT7	4.30759108	10.22851875	1.247644029	2.35E−24	3.77E−23
PCYT2	15.79421824	27.65662255	0.808228454	6.16E−10	1.27E−09
GMPS	2.12571058	5.125880879	1.269854768	3.78E−23	4.40E−22
SGPL1	5.76193592	8.91802992	0.630171424	5.88E−09	1.11E−08
PYCR1	0.861568492	8.121217599	3.236658648	9.35E−06	1.45E−05
PIPOX	96.0303444	72.54766923	−0.404561087	4.98E−06	7.84E−06
MDH1	20.8540302	33.17323875	0.669693649	3.56E−14	1.07E−13
PAFAH1B3	2.183462234	13.95513656	2.676106759	2.23E−19	1.19E−18
ETNK1	3.83300098	5.261446108	0.456985012	0.000160517	0.000221554
PRPS2	6.2703327	8.859932778	0.498753759	0.003710583	0.004615306
ENTPD8	4.7108131	3.744468425	−0.33121517	1.12E−05	1.73E−05
ALOX12	0.109011063	0.275266308	1.336353479	1.04E−12	2.76E−12
ACACB	8.46489348	5.654933634	−0.581981821	3.48E−09	6.71E−09
GMDS	3.66597734	7.7145025	1.073375254	3.91E−10	8.20E−10
ME3	0.531760212	1.606608404	1.595170589	3.70E−15	1.21E−14
PIK3CA	1.177370638	1.455137701	0.305587128	0.039746951	0.045262365
DCK	1.436440984	3.648930365	1.344974898	1.32E−15	4.39E−15
GYS1	1.954834784	4.327244832	1.146402068	3.57E−18	1.63E−17
ADO	2.54632174	3.430583608	0.430039305	5.05E−05	7.37E−05
AGPAT1	8.15078736	20.35100554	1.320088745	2.02E−24	3.31E−23
ADSL	3.82300396	8.444661914	1.143332971	6.03E−24	8.28E−23
ALDH6A1	64.384127	28.49727636	−1.175881016	9.37E−20	5.28E−19
INPP5A	3.35817814	4.788868444	0.512006041	1.30E−07	2.25E−07
MTMR7	0.146421709	0.848734424	2.535183728	1.88E−05	2.83E−05
SULT1A1	13.47559808	9.050285627	−0.574314075	3.86E−09	7.41E−09
CYP26A1	6.569500214	0.658808858	−3.31785176	1.48E−21	1.21E−20
PDHB	10.74619842	14.41091844	0.423335903	4.93E−09	9.42E−09
ALDH1B1	67.6148248	40.63371028	−0.734662494	5.75E−11	1.32E−10
ACOX1	33.2083646	25.9762862	−0.354351492	1.12E−07	1.94E−07
PCYT1A	3.16719152	5.454631679	0.784277673	2.39E−20	1.42E−19
NEU1	7.53752836	26.66679468	1.822880995	3.40E−28	3.93E−26
POLR3G	0.27411095	0.623002221	1.184477344	3.97E−13	1.11E−12
GPD1L	0.656858692	1.902400678	1.534166188	5.88E−09	1.11E−08
TSTA3	11.35656626	26.20332018	1.206222933	9.59E−18	4.11E−17
AK1	2.37631508	3.937539076	0.728568102	1.41E−08	2.57E−08
PRODH2	39.0309294	28.08474289	−0.474831221	1.12E−07	1.94E−07
AOC1	1.798183299	0.745771443	−1.269734631	0.001027935	0.001333489
SPTLC2	1.75140612	2.742051601	0.646742062	3.29E−10	6.95E−10
KHK	82.384005	61.54386532	−0.420749206	1.71E−07	2.97E−07
DGKQ	1.457440362	4.03086916	1.467654105	4.12E−23	4.65E−22
ACAA2	105.504459	52.75512735	−0.99992075	5.19E−20	2.99E−19
ADPRM	2.7431114	3.986350382	0.53925531	1.35E−05	2.06E−05
AGXT2	21.68184318	9.089264569	−1.254251933	2.47E−18	1.14E−17
TYMP	15.65976866	31.83914656	1.023738765	1.52E−06	2.49E−06
SYNJ2	1.00822186	2.334073703	1.211036978	8.08E−10	1.65E−09
UPB1	32.3556494	25.21832405	−0.359545233	0.00015816	0.000219102
ENTPD2	0.372271672	1.96584486	2.400721728	3.97E−12	9.92E−12
GPX1	124.116905	222.3074623	0.840856749	1.77E−10	3.88E−10
PRPS1	12.66083042	18.44318764	0.542715993	3.69E−06	5.87E−06
GLB1	9.81417212	18.17416942	0.888950954	8.00E−18	3.51E−17
DHDH	0.083578928	0.534516557	2.677023475	3.88E−10	8.16E−10
PTGES2	7.21657504	16.7167287	1.211906346	8.10E−22	7.03E−21
NME1	5.12519878	17.66287993	1.785040727	4.09E−24	5.94E−23
GLYCTK	55.138394	52.81353627	−0.062149503	0.028147703	0.032445062
TKFC	25.9085641	18.57767451	−0.479859139	9.44E−08	1.65E−07
ADCY3	0.561033466	1.082943581	0.948799348	0.000235869	0.000319141
HAGHL	0.048049798	0.557557535	3.536518411	1.02E−21	8.52E−21
UGT2A1	0.98181545	0.696545876	−0.495233494	0.000447104	0.000590145
PTGS2	0.685695488	0.209820152	−1.708414783	5.31E−17	2.01E−16
ALDOB	2772.70035	1401.228338	−0.98459964	1.94E−14	5.97E−14
TYRP1	0.008600457	0.267724997	4.960194749	0.000375099	0.000501238
NUDT9	8.49499344	10.95659974	0.367115405	0.000437831	0.000578918
MPI	2.65308182	4.29403827	0.694665881	6.65E−13	1.80E−12
PPAT	0.863819914	1.765653998	1.031400175	3.59E−13	1.02E−12
GPD2	0.764990188	1.774788278	1.214133781	1.94E−12	4.95E−12
DTYMK	2.81252568	11.25842326	2.001066619	4.78E−28	4.51E−26
GBA3	29.65604208	9.090840342	−1.705840501	2.15E−20	1.29E−19
PGS1	1.087267554	2.837714227	1.384022308	3.10E−24	4.88E−23
ITPA	8.35692768	20.76255349	1.312939329	2.62E−22	2.42E−21
GSTA2	171.5788165	122.8201096	−0.482324649	3.52E−06	5.61E−06
ENPP2	4.152915006	9.060232331	1.1254237	9.16E−05	0.000130485
GLDC	19.3329667	15.99389614	−0.273541615	0.001078603	0.001394427
PKM	4.01863632	25.24352982	2.65113573	1.86E−14	5.79E−14
MTMR6	2.721699042	3.270919686	0.26518879	0.033367063	0.038285916
UPP1	2.553424264	3.724478367	0.544605114	0.000545856	0.000715488
RRM2	0.469398657	6.378298565	3.764286013	3.18E−26	1.33E−24
ACSL6	0.2617771	1.020156731	1.962380012	0.035460273	0.040503035
LDHA	83.2189104	80.19834618	−0.053338913	0.042299176	0.047951769
GPI	20.7880496	42.19970111	1.021478374	1.93E−16	7.02E−16
ACSM5	50.4663964	20.22345997	−1.319293229	2.44E−17	9.70E−17
PMM1	7.81445296	10.63144872	0.444121415	0.001111858	0.001432513
HADH	41.8828638	35.57719138	−0.235407469	5.47E−05	7.95E−05
SAT2	85.0755162	76.44819578	−0.154261546	0.006385788	0.00776372
GNE	25.03165064	13.02163252	−0.942843099	2.36E−17	9.45E−17
GSTM2	0.271784875	0.548345501	1.012620017	0.000818116	0.001066801
AKR1C2	16.24967136	50.10020952	1.624406096	5.01E−06	7.87E−06
CDO1	96.0541018	74.41269956	−0.368298364	2.75E−05	4.08E−05
CNDP1	6.560749296	1.024432657	−2.679035444	1.45E−22	1.42E−21
AHCYL1	15.2581398	20.6807703	0.438710837	2.04E−05	3.05E−05
ACP6	0.71604374	1.761317951	1.298535743	2.41E−20	1.42E−19
NME7	0.643526996	1.362272741	1.081942999	4.98E−17	1.90E−16
ADH1C	476.144326	233.435638	−1.02837411	1.14E−12	3.00E−12
TBXAS1	1.383732803	1.149415109	−0.267665469	0.000395307	0.000526379
BAAT	190.9706308	179.3099227	−0.090895458	0.01578084	0.018472146
AOC2	0.219990869	0.495573818	1.171656326	1.73E−09	3.44E−09
AGL	6.99793654	3.850530943	−0.861872192	9.11E−17	3.36E−16
IDO2	0.862516082	0.34741407	−1.311895173	9.61E−15	3.06E−14
UMPS	3.1273573	4.722024104	0.59446135	3.46E−13	9.83E−13
RDH11	16.88686872	20.31333093	0.26652499	0.041311007	0.04690197
AADAT	11.0372536	2.431650612	−2.182373373	7.44E−26	2.16E−24
ME1	2.644506866	8.410134567	1.669130164	0.000433795	0.000574588
ACSM2A	35.2888359	22.45481559	−0.652186966	3.27E−08	5.87E−08
CYP2C8	449.461848	100.5747926	−2.159929897	6.04E−26	1.82E−24
LCMT1	2.3080183	5.778087404	1.323937364	5.58E−25	1.20E−23
PLA2G12A	5.69083708	5.41609293	−0.071388385	0.004386592	0.005385166
AZIN2	0.124387656	0.277501557	1.157652541	9.96E−10	2.02E−09
PTGIS	2.191950054	1.166763256	−0.909703067	2.41E−14	7.37E−14
GPX4	118.53575	188.6938862	0.670725443	1.28E−10	2.86E−10
RRM1	3.92014216	10.15498263	1.373209893	1.84E−21	1.42E−20
ACAA1	48.7472452	25.20723697	−0.951482702	4.88E−18	2.21E−17
NNMT	469.3829578	140.666585	−1.738485801	1.15E−17	4.88E−17
TDO2	85.2300468	35.60804767	−1.259158787	2.05E−15	6.71E−15
ADCY6	1.052476086	3.845742664	1.869474775	3.47E−25	7.71E−24
SARDH	34.544682	18.5565624	−0.896534155	1.76E−17	7.30E−17
RFK	5.10336714	7.08934908	0.474203736	1.99E−05	3.00E−05
GAMT	189.2736142	172.100408	−0.137222788	0.0195276	0.022717008
ACYP1	0.435832892	1.541828841	1.822795635	4.19E−26	1.44E−24
MTHFD1	36.8332948	23.39997829	−0.654503263	3.30E−11	7.65E−11
GNMT	134.7991696	71.11434267	−0.922599145	2.73E−11	6.39E−11
POLR2E	23.3947028	33.18577334	0.504382993	1.39E−11	3.40E−11
PAFAH2	5.46319746	4.725647625	−0.209233512	7.56E−05	0.000108525
RRM2B	3.53135628	5.709601422	0.693167654	1.77E−08	3.21E−08
CYP1A2	162.0610482	24.79812246	−2.708234578	4.83E−24	6.89E−23
ALDH18A1	7.39242978	15.6829565	1.085077018	4.28E−15	1.39E−14
HDC	0.257674991	0.232402497	−0.148926952	0.000172793	0.000238063
GANC	0.61022308	1.019814907	0.740898679	3.01E−10	6.41E−10
ADCY1	1.645927202	1.296404921	−0.344384126	1.17E−09	2.34E−09
FBP1	394.352886	138.9132655	−1.505302825	4.76E−21	3.27E−20
POLR1C	3.35005178	6.978792797	1.058794103	2.33E−21	1.74E−20
GLS	1.201759816	4.164693323	1.793061676	3.87E−12	9.70E−12
LPCAT2	0.474627134	1.255891704	1.40384558	0.000234735	0.000318178
P4HA2	1.008157448	4.303512033	2.093793535	1.64E−22	1.57E−21
CKB	3.60284462	23.67772586	2.716322184	3.20E−06	5.13E−06
ITPKA	0.460224664	4.393506423	3.254962596	5.55E−18	2.48E−17
POLR2C	9.07856552	14.29939552	0.655417896	2.19E−10	4.77E−10
COX10	2.5153264	3.46926254	0.4638834	0.000317057	0.000425939
POLR2L	45.7742558	93.97066345	1.037674004	3.18E−19	1.66E−18
ALDH3B1	1.04183392	2.518889866	1.27366273	1.40E−06	2.30E−06
ITPKB	0.905496968	1.49022587	0.718749297	4.85E−05	7.09E−05
TXNRD1	6.76314148	28.10662419	2.055144747	1.93E−17	7.90E−17
CA5B	0.142795734	0.550149828	1.945871693	6.48E−16	2.22E−15
PLCB1	0.225675628	1.091508615	2.274000988	4.77E−14	1.42E−13
NUDT5	9.44814408	17.61621736	0.898801304	1.61E−21	1.30E−20
ODC1	18.19301946	33.29143499	0.871766053	1.65E−08	3.00E−08
ACACA	1.393724136	3.919558477	1.491746117	6.37E−20	3.62E−19
INPP5J	0.03995144	0.299135465	2.904479538	2.70E−11	6.36E−11
DNMT3B	0.128279551	0.715787579	2.480240297	7.59E−21	4.97E−20
PDE7A	0.574264186	1.45451893	1.340755577	3.24E−14	9.82E−14
INPPL1	4.00731938	9.917278334	1.30730675	4.82E−21	3.28E−20
POLR3F	1.290860464	2.890671173	1.163071445	2.94E−25	6.72E−24
DPYS	89.3997666	56.69502639	−0.657048886	2.46E−09	4.82E−09
CBR3	0.238376351	1.432379474	2.587100731	8.94E−16	3.01E−15
CA2	43.516094	27.05355184	−0.68573105	1.63E−13	4.71E−13
PLA2G1B	0.358601163	2.766570668	2.947646705	2.58E−09	5.01E−09
NIT2	8.36875624	7.98024058	−0.068580986	0.03755159	0.042826964
PAFAH1B2	5.55860366	8.178248219	0.557069332	2.81E−10	6.00E−10
GYS2	38.5893622	12.55410139	−1.620044433	4.34E−21	3.00E−20
GLUL	58.1799336	366.9451525	2.656970884	0.000190569	0.000261125
GPT2	34.4213786	29.04180666	−0.245173675	0.000173655	0.000238815
FMO2	0.513662603	0.392428653	−0.388390661	1.83E−05	2.78E−05
TXNDC12	10.49130104	17.80804426	0.763335484	7.64E−21	4.97E−20
MTMR2	0.825414542	1.902368228	1.204605765	2.99E−11	6.94E−11
AHCYL2	1.248491948	1.918117883	0.619504871	8.41E−07	1.40E−06
ALDOA	20.55360858	72.12748631	1.811157437	2.08E−19	1.12E−18
MDH2	46.934258	79.50191043	0.760348177	6.89E−16	2.34E−15
FADS2	9.642111654	20.05759983	1.056727937	0.011186336	0.013321267
PAPSS1	2.28088236	5.466021302	1.260899043	7.56E−17	2.81E−16
NANP	0.94921358	1.727478428	0.863863049	2.01E−15	6.61E−15
MAT1A	492.371978	208.7499778	−1.237972613	2.22E−21	1.68E−20
GSTA4	3.88265552	12.89632448	1.73184433	2.91E−16	1.03E−15
ADCY4	0.314709281	0.7262269	1.206400646	6.53E−13	1.77E−12
INPP4B	0.149343996	0.294366749	0.97897548	0.001456753	0.001857852
UGT1A10	0.008250359	0.550736449	6.060761396	0.01163501	0.013812001
NOS2	0.097262382	0.402505335	2.049054083	5.21E−13	1.44E−12
ACER3	0.859076874	1.427533057	0.732665013	9.70E−08	1.69E−07
PIK3C3	0.909520322	1.266871369	0.47809227	2.98E−07	5.07E−07
HAAO	80.8500472	45.99512303	−0.813767719	3.64E−12	9.15E−12
DEGS1	7.60895486	17.27093585	1.182576051	6.59E−18	2.93E−17
NUDT2	7.28607756	17.12103503	1.232555663	4.35E−19	2.20E−18
ADH1B	527.994414	221.1341117	−1.255601077	3.38E−17	1.31E−16
POLR2I	11.61097278	19.34012161	0.736108019	2.47E−10	5.33E−10
NT5M	0.40577981	1.573026546	1.954774029	3.24E−17	1.27E−16
GATM	201.0644772	134.4996308	−0.580056005	1.14E−08	2.11E−08
PLPP3	35.3993676	17.43031475	−1.022124966	3.08E−17	1.22E−16
PHPT1	13.54757584	48.38776828	1.836607678	3.87E−26	1.43E−24
AKR1B10	21.56249119	348.4054764	4.014171528	1.73E−11	4.18E−11
DGKA	0.329520905	0.633643682	0.943301801	2.37E−05	3.54E−05
TREH	1.851689492	1.32430031	−0.483611878	3.78E−06	6.00E−06
UGT1A6	2.955261072	7.241389897	1.292981046	0.006980058	0.008405017
B4GALT2	5.90955282	11.4905014	0.959320883	1.47E−17	6.13E−17
SGMS2	3.765996564	1.802598975	−1.062953208	1.68E−05	2.55E−05
ENTPD1	0.634465836	1.48168498	1.223624363	2.79E−19	1.46E−18
INPP5E	1.353489428	3.225346035	1.252770331	1.93E−19	1.05E−18
AMPD2	4.28833292	7.542024427	0.814534911	7.54E−13	2.02E−12
KYNU	2.4308026	2.247638585	−0.113022668	9.84E−07	1.63E−06
UGP2	58.5313736	40.07029916	−0.546676855	1.31E−10	2.91E−10
CDA	29.0720696	17.30167458	−0.7487221	1.20E−10	2.69E−10
NME1-NME2	0.318942837	0.531576005	0.736978107	0.000431791	0.000572938
DGUOK	11.27155494	19.37659762	0.781628714	1.50E−13	4.35E−13
AK2	14.9739754	17.94421295	0.261061357	0.000889768	0.001158232
PI4KA	2.861807906	5.047843187	0.818740256	2.57E−10	5.53E−10
COX15	6.69914978	8.729761864	0.381964291	2.57E−08	4.65E−08
CHST13	13.29861354	25.15093577	0.919336234	5.40E−07	9.06E−07
GLO1	32.9487238	51.30883037	0.638984552	5.19E−09	9.87E−09
HAO2	86.8433924	25.34855366	−1.776512651	1.58E−20	9.67E−20
GCDH	24.69117048	14.79007361	−0.739365996	2.95E−12	7.47E−12
CPT1B	0.121235197	0.398590265	1.717097872	1.74E−14	5.44E−14
SPTLC1	3.060500882	4.809781218	0.652203488	1.51E−05	2.29E−05
PYGB	4.35291446	18.82163409	2.112338313	8.70E−26	2.35E−24
POLR2H	5.75387058	12.47900765	1.116898537	1.04E−22	1.07E−21
POLR2B	4.42658506	7.625983009	0.784729173	7.24E−11	1.65E−10
RETSAT	38.7021028	37.57376368	−0.04268632	0.019656262	0.022831504
PTDSS2	1.662328	4.164425941	1.324912563	2.15E−23	2.57E−22
PYCR2	5.37894786	15.26388831	1.50472661	5.48E−28	4.59E−26
METTL2B	1.507055692	2.85584579	0.922185347	1.48E−21	1.21E−20
DGKH	0.113162972	0.327426191	1.532767749	3.75E−16	1.31E−15
GSTA1	630.2036156	548.263622	−0.200948279	0.00110705	0.001428757
MAT2B	7.7057353	10.40578761	0.433381633	4.64E−06	7.33E−06
BPNT1	5.53523458	10.96770642	0.986545495	3.39E−21	2.44E−20
DNMT3A	0.555289364	2.215351506	1.996223959	7.94E−24	1.07E−22
SEPHS2	127.9557976	190.1067404	0.571164168	2.59E−10	5.56E−10
POLE3	8.66220048	16.03828744	0.888714631	6.27E−19	3.13E−18
INPP4A	0.504742884	0.934387362	0.888472096	7.54E−11	1.70E−10
CYP2E1	530.2645806	266.8712181	−0.990568666	5.25E−11	1.21E−10
CYP2J2	34.1905172	23.20978966	−0.558862799	5.17E−11	1.19E−10
ITPK1	7.76110164	15.32881022	0.981912369	6.29E−16	2.16E−15
POLR2D	3.37104148	5.942900711	0.817972898	1.73E−20	1.05E−19
CSAD	8.06462968	6.521514989	−0.306401137	7.35E−05	0.000105697
NADSYN1	1.32031081	2.690367867	1.026925864	1.07E−21	8.84E−21
AMD1	4.19170934	5.335768195	0.348157311	0.011707199	0.01387588
GSTM5	0.363642937	0.231850861	−0.649325476	3.60E−10	7.60E−10
GAL3ST1	0.673563029	6.610136193	3.294795138	0.00012214	0.000170769
GPT	47.2407036	38.45088064	−0.297013812	4.98E−06	7.84E−06
AGPAT3	8.2516119	11.64639678	0.497135802	4.54E−07	7.67E−07
POLR3B	2.42816858	2.951770627	0.281712031	0.003889012	0.004829283
ASL	53.904885	52.08163964	−0.049641151	0.001055415	0.001366789
PLA2G15	2.72944324	4.31619806	0.661154372	5.77E−08	1.02E−07
BDH2	10.50709572	6.470275333	−0.699464937	1.71E−12	4.41E−12
DGKD	0.77171494	1.905129995	1.303749501	4.07E−19	2.09E−18
ADI1	91.0607558	69.40997785	−0.391686365	2.37E−07	4.07E−07
CHPT1	14.3208144	19.59827237	0.452612945	1.36E−06	2.24E−06
NME3	9.19139972	22.42554503	1.286786564	6.38E−22	5.60E−21
DHODH	15.80542924	6.798435324	−1.217145566	4.99E−16	1.73E−15
LCAT	68.1154072	16.82335661	−2.01751558	2.17E−26	9.64E−25
GLS2	4.778706618	1.78680446	−1.419238437	1.24E−15	4.17E−15
PLPP1	14.99118618	30.87743991	1.042438601	9.08E−10	1.85E−09
CKMT1B	0.007144548	0.294667545	5.366101652	0.004188776	0.005159096
ENTPD4	1.775126334	2.540169076	0.517002824	0.002229478	0.002814809
PLCD1	1.691310116	2.545895876	0.590032202	2.56E−07	4.37E−07
G6PC	204.0482294	152.8745478	−0.416561961	7.14E−06	1.12E−05
ATIC	7.75802162	18.69461756	1.268862257	1.56E−23	1.96E−22
PEMT	52.8566338	30.87396532	−0.775693761	4.96E−14	1.47E−13
AMY2B	0.407575554	0.944786186	1.212920347	3.39E−06	5.43E−06
MGST1	91.208676	75.16583894	−0.279093923	0.000239299	0.000323204
CBS	1.188079795	0.956133191	−0.313348227	0.028413017	0.032700957
PFKFB4	0.151297184	0.756509371	2.321972815	5.38E−18	2.42E−17
MTHFD1L	0.557753522	2.524923016	2.178539778	2.35E−22	2.19E−21
SUOX	10.01822034	9.128650786	−0.134152697	0.011544688	0.013726361
ACP2	20.4062632	24.48232224	0.262728389	0.004059381	0.005016093
GOT1	154.184527	113.1036936	−0.447011949	1.05E−06	1.73E−06
MCEE	8.66946072	8.069239048	−0.103509624	0.013633303	0.016032934
POLR3C	2.99792546	7.304838	1.284887766	1.45E−22	1.42E−21

### Analysis of the differential expression and prognostic relationship of DEGs in HCC based on TCGA database

**Figure 3:**
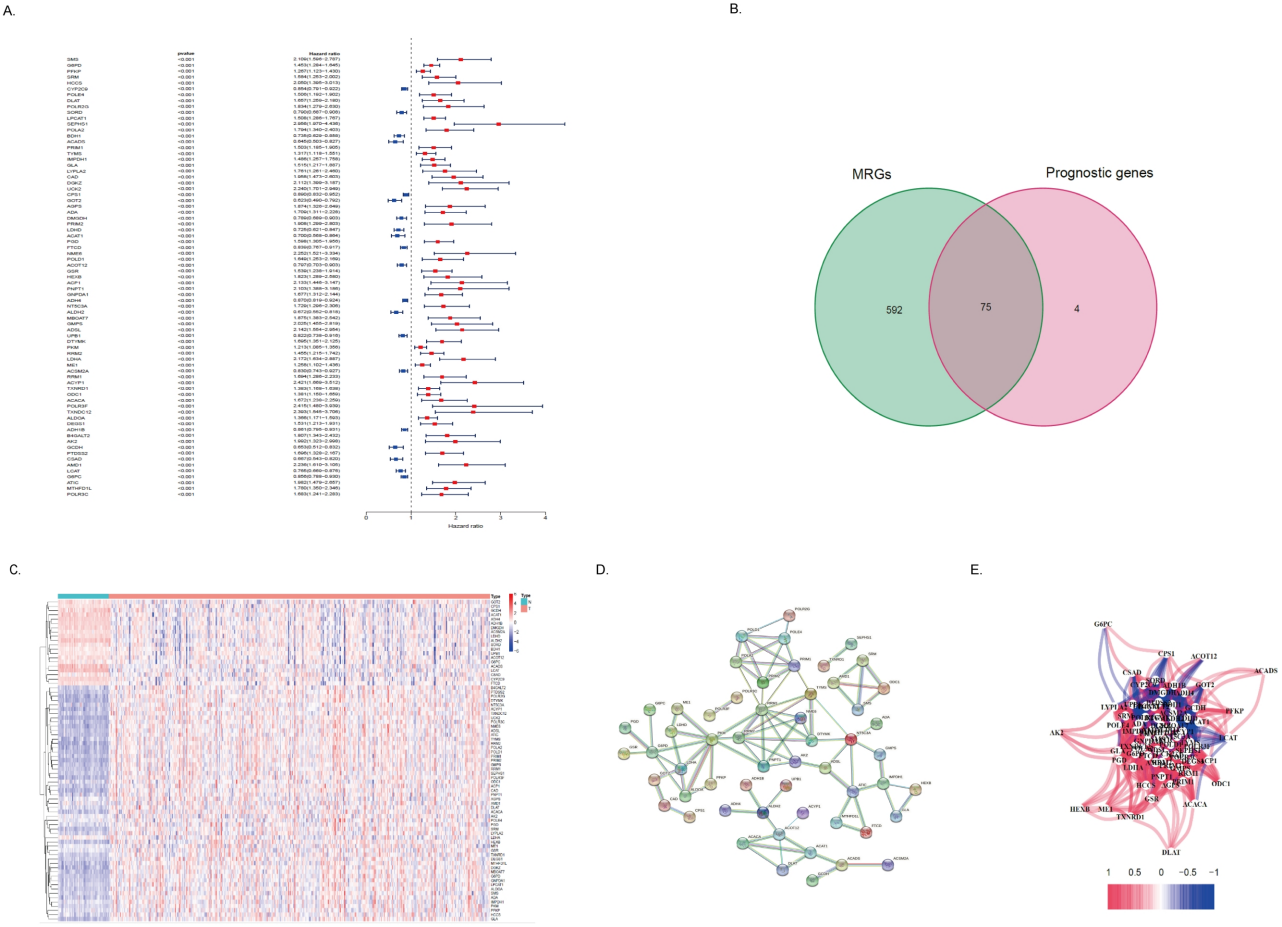
Identification of the metabolism-related DEGs with prognostic values in the TCGA cohort. (A) Forest plot shows the results of univariate Cox regression analysis between gene expression and OS. (B) Venn diagram shows the intersection of differentially expressed MRGs and prognostic-related genes. (B) The heatmap shows the expression difference of these 75 MRGs with a prognostic value. (D) The PPI network showed the interaction between 75 genes. (E) A correlation network of 75 MRGs with prognostic value. The correlation coefficient is distinguished by different colors.

By using univariate COX regression analysis, we found that 79 DEGs in tumor tissues of HCC patients were associated with the prognosis of HCC patients, of which 21 genes with high expression indicated a better prognosis and the other 58 genes were associated with a poor prognosis (Fig. 3A). Of these, 75 genes were MRGs (Fig. 3B). Among the 75 MRGs associated with the prognosis of HCC patients, 55 genes were highly expressed in tumor tissues of HCC patients, and 20 genes were poorly expressed. We used heatmaps for illustration (Fig. 3C). Then, we evaluated the protein-protein interactions of these genes, further revealing the strong interaction activity of these molecules at the protein level, as shown in Fig. 2D. Similarly, correlated networks built based on mRNA expression levels in TCGA showed a negative (blue) and positive (red) correlation between these prognostically relevant MRGs, as shown in Fig. 3E.

### Development and evaluation of metabolism-related risk score

**Figure 4:**
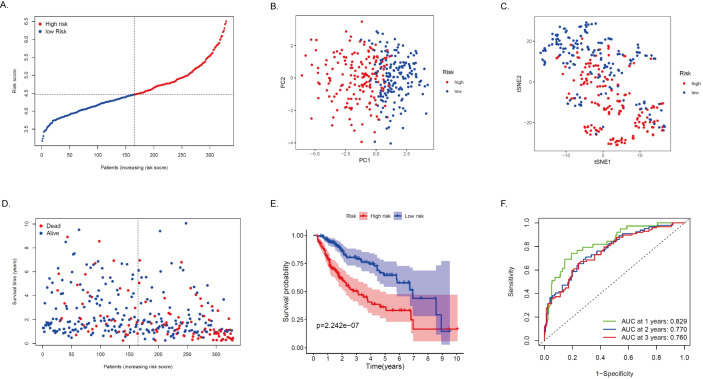
Construction and verification of the MRRS in the TCGA cohort. (A) The risk score distribution of HCC patients in the TCGA database. The color from blue to red indicates the level of expression from low to high. Scatter plots of high-risk and low-risk risk scores. (B) PCA analysis of CRA patients. (C) T-SNE analysis of CRA patients. (D) Survival status and survival time of patients. Red dots indicate patients who have died, and blue dots indicate that they are still alive. (E) Survival curves of low-risk and high-risk populations in HCC patients. Red represents the high-risk group, and blue represents the low-risk group. (F) 1-year, 2-year, and 3-year AUCs. AUC, the area under the curve; ROC, receiver operating characteristic.

The expression profiles of the above 75 genes were used to establish a prognostic model by LASSO Cox regression analysis. Based on the optimal value of *λ*, 14 genes that contribute the most to the prognosis of HCC patients were identified (Table 2). Among these genes, except for ACADS, GOT2, ADH4, and CSAD, high expression of the other 10 genes was associated with a poor prognosis in HCC patients (*P* < 0.05). The risk score is calculated as follows: risk score = (0.0875 * DLAT expression) + (0.2953 * SEPHS1 expression) + (-0.1116 * ACADS expression) + (0.1978 * UCK2 expression) + (-0.0143 * GOT2 expression) + (-0.0295 * ADH4 expression) + (-0.3244 * LDHA expression) + (0.0520 * ME1 expression) + (0.0105 * TXNRD1 expression) + (0.0433 * B4GALT2 expression) + (0.1975* AK2 expression) + (0.1783* PTDSS2 expression) + (-0.023 * CSAD expression) + (0.0207 * AMD1 expression). Based on the median of the risk score, the sample was rated as low and high risk (Fig. 4A). Of these, 164 patients were in the high-risk group and 165 patients were in the low-risk group. Principal component analysis (PCA) and t-distribution random neighborhood embedding (t-SNE) analysis showed that patients in different risk groups were distributed in two directions (Figs. 4B–4C). This suggests that the MRRS model can distinguish well between high- and low-risk patients. It is shown that the patients in the high-risk group had a higher probability of death than those in the low-risk group (Fig. 4D). In addition, Kaplan–Meier survival analysis also confirmed that the survival time of the high-risk group was significantly reduced compared to the low-risk group (Fig. 4E, *P* < 0.001). The predictive performance of the model was evaluated using a time-varying ROC curve, with the area under the curve (AUC) reaching 0.829 at 1 year, 0.770 at 2 years, and 0.760 at 3 years (Fig. 4F). Overall, these findings suggest that MRRS was able to distinguish the prognosis of HCC patients in the TCGA database.

**Table 2:** The genes that contribute to the prognosis of HCC patients. Based on the optimal value of *λ*, 14 genes that contribute the most to the prognosis of HCC patients were identified.

Gene	Coef
DLAT	0.087527704
SEPHS1	0.295255334
ACADS	−0.111567502
UCK2	0.197793632
GOT2	−0.014390167
ADH4	−0.029554667
LDHA	0.324459855
ME1	0.052008356
TXNRD1	0.010457298
B4GALT2	0.043281636
AK2	0.19745112
PTDSS2	0.178350248
CSAD	−0.023302247
AMD1	0.02060711

### Validation of MRRS in the ICGC cohort

**Figure 5:**
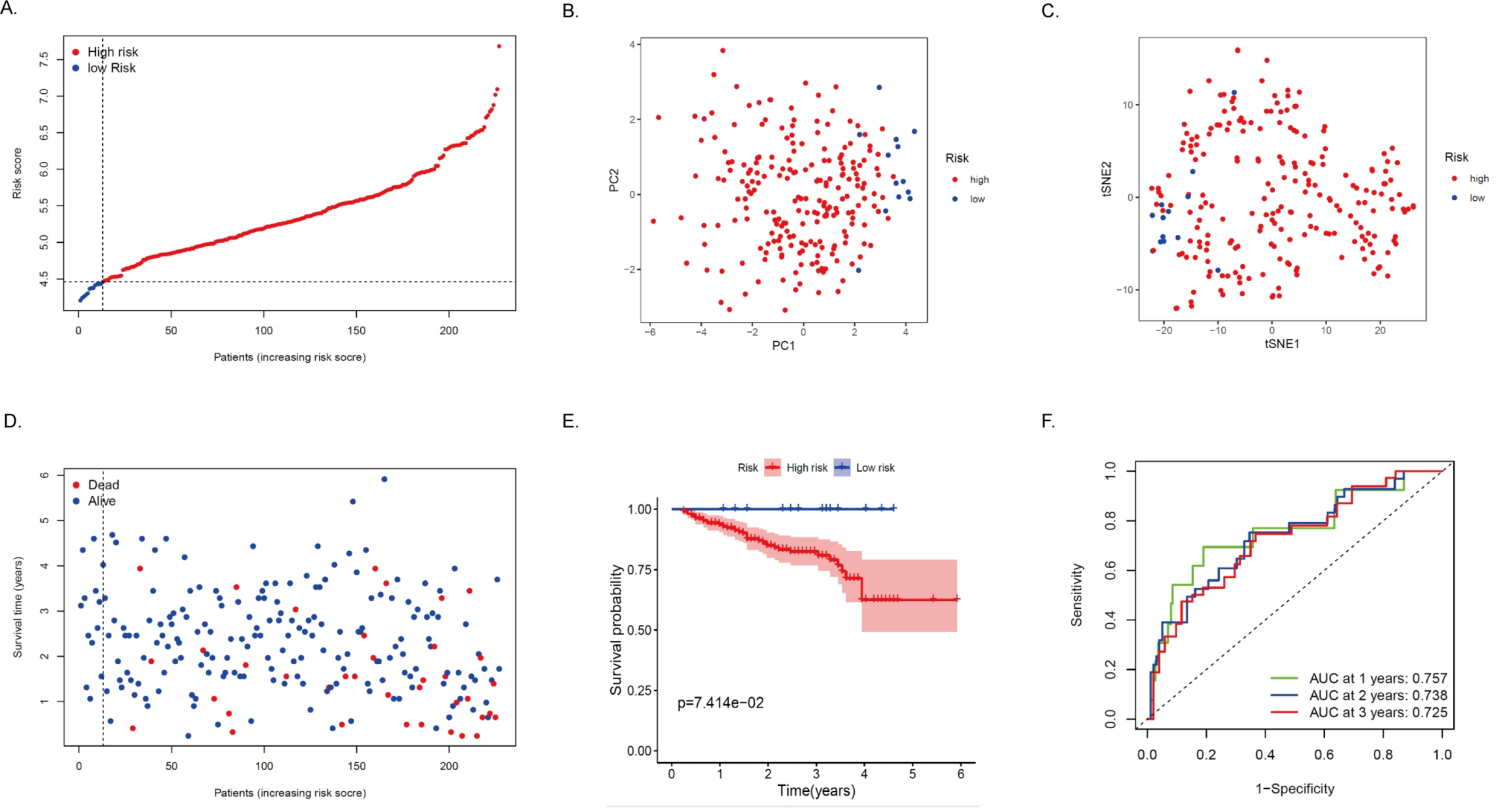
Validation of the MRRS in the ICGC cohort. (A) The risk score distribution of HCC patients in the ICGC database. The color from blue to red indicates the level of expression from low to high. Scatter plots of high-risk and low-risk risk scores. (B) PCA analysis of CRA patients. (C) T-SNE analysis of CRA patients. (D) Survival status and survival time of patients. Red dots indicate patients who have died, and blue dots indicate that they are still alive. (E) Survival curves of low-risk and high-risk populations in HCC patients. Red represents the high-risk group, and blue represents the low-risk group. (F) 1-year, 2-year, and 3-year AUCs. AUC, the area under the curve; ROC, receiver operating characteristic.

To test the robustness of the model developed by the TCGA cohort, we validated the model with HCC patients’ data from the ICGC database. We calculated the risk score for each patient in the ICGC cohort using the same formula. Based on the above median, we divided HCC patients from the ICGC cohort into high-risk (*n* = 214) and low-risk (*n* = 13) (Fig. 5A). In the ICGC cohort, the results of PCA and t-SNE analyses were similar to those of TCGA (Figs. 5B–5C). The high-risk group had a worse prognosis than the low-risk group (Fig. 5D). The survival curve showed that MRRS was effective in differentiating patients with different prognostic conditions, and patients in the high-risk group tended to have a higher risk of death and significantly shorter survival times than those in the low-risk group (Fig. 5E, *P* < 0.05). ROC analysis showed that MRRS had good predictive power on the prognosis of patients in the ICGC cohort (AUC = 0.757, 0.738, and 0.725; at 1, 2, and 3 years, respectively in ICGC, Fig. 5F). Taken together, this evidence shows that the model was able to predict the prognosis of HCC patients well.

### Independent prognostic value of MRRS in patients with HCC

**Figure 6:**
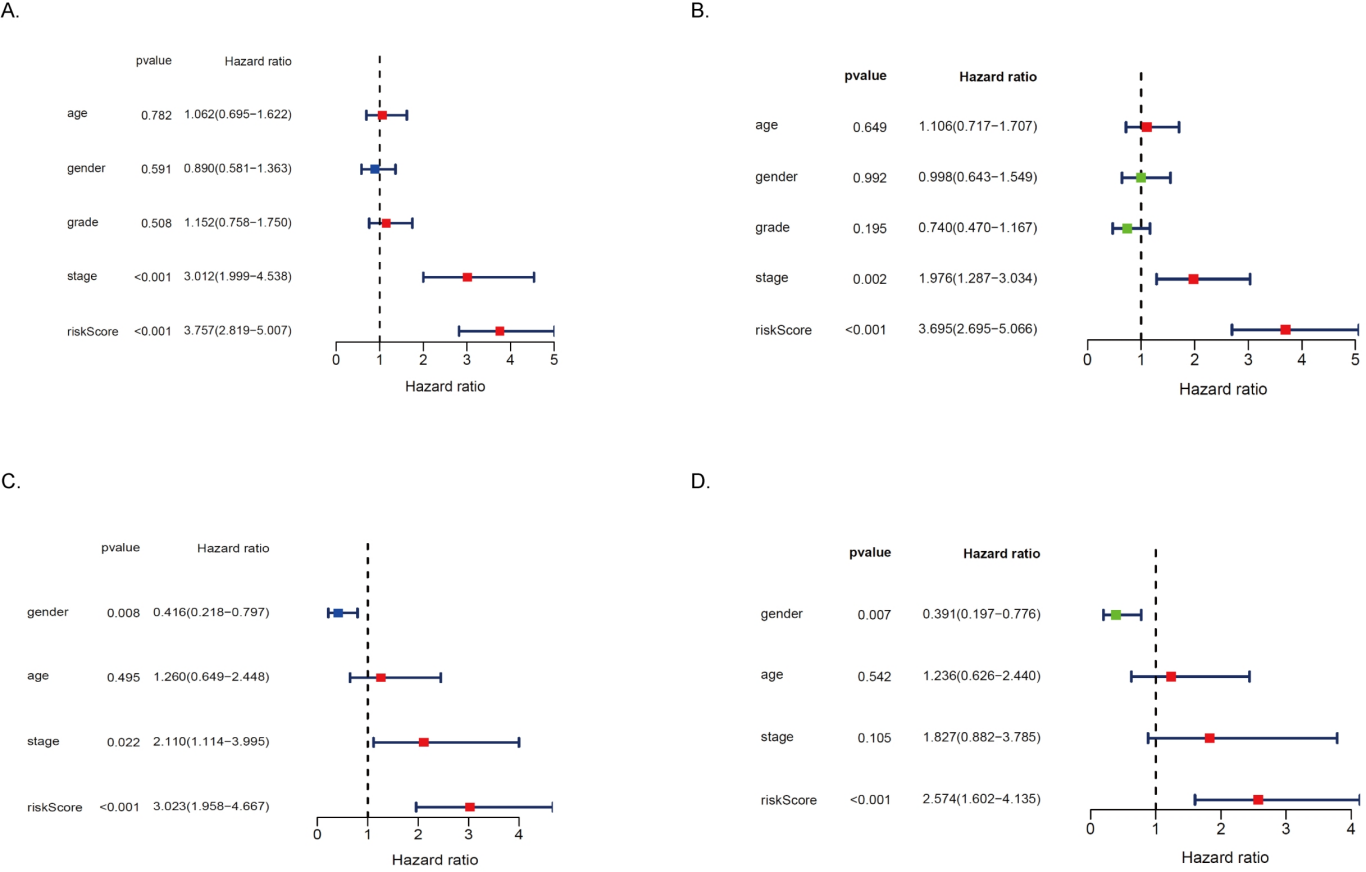
MRRS is an independent prognostic signature for HCC patients. (A) In the TCGA cohort, risk factors analysis of OS in the univariate Cox regression. (B) In the TCGA cohort, risk factors analysis of OS in the multivariate Cox regression. (C) In the ICGC cohort, risk factors analysis of OS in the univariate Cox regression. (D) In the ICGC cohort, risk factors analysis of OS in the multivariate Cox regression.HR hazard ratio.

To further determine whether MRRS can be used as an independent predictor of prognosis in HCC patients, we first performed univariate and multivariate Cox regression analysis in the TCGA cohort. As shown in Figs. 6A–6B, MRRS was found to be significantly correlated with the prognosis of HCC patients in both univariate Cox regression analyses (Hazard ratio (HR) = 3.757, 95% CI [2.819–5.007], *p* < 0.001) and multivariate Cox regression analysis (HR = 3.695, 95% CI [2.695–5.066], *p* < 0.001). Univariate and multivariate Cox regression analyses were then performed in the ICGC cohort for validation. The results showed that MRRS remained an independent predictor of prognosis in the ICGC cohort (HR = 3.023, 95% CI [1.958–4.667], *p* < 0.001, and HR = 2.574, 95% CI [1.602–4.135], *p* < 0.001; Figs. 6C–6D). In addition, the multivariate analysis to remove confounding factors, age, sex, and stage could not be used as independent prognostic indicators for HCC patients, showing the superiority of MRRS in the prognosis assessment of HCC patients. Overall, our study suggests that MRRS can serve as an independent prognostic factor for patients with HCC.

### GO and KEGG analysis of DEGs in high and low-risk groups in the TCGA and ICGC cohorts

**Figure 7:**
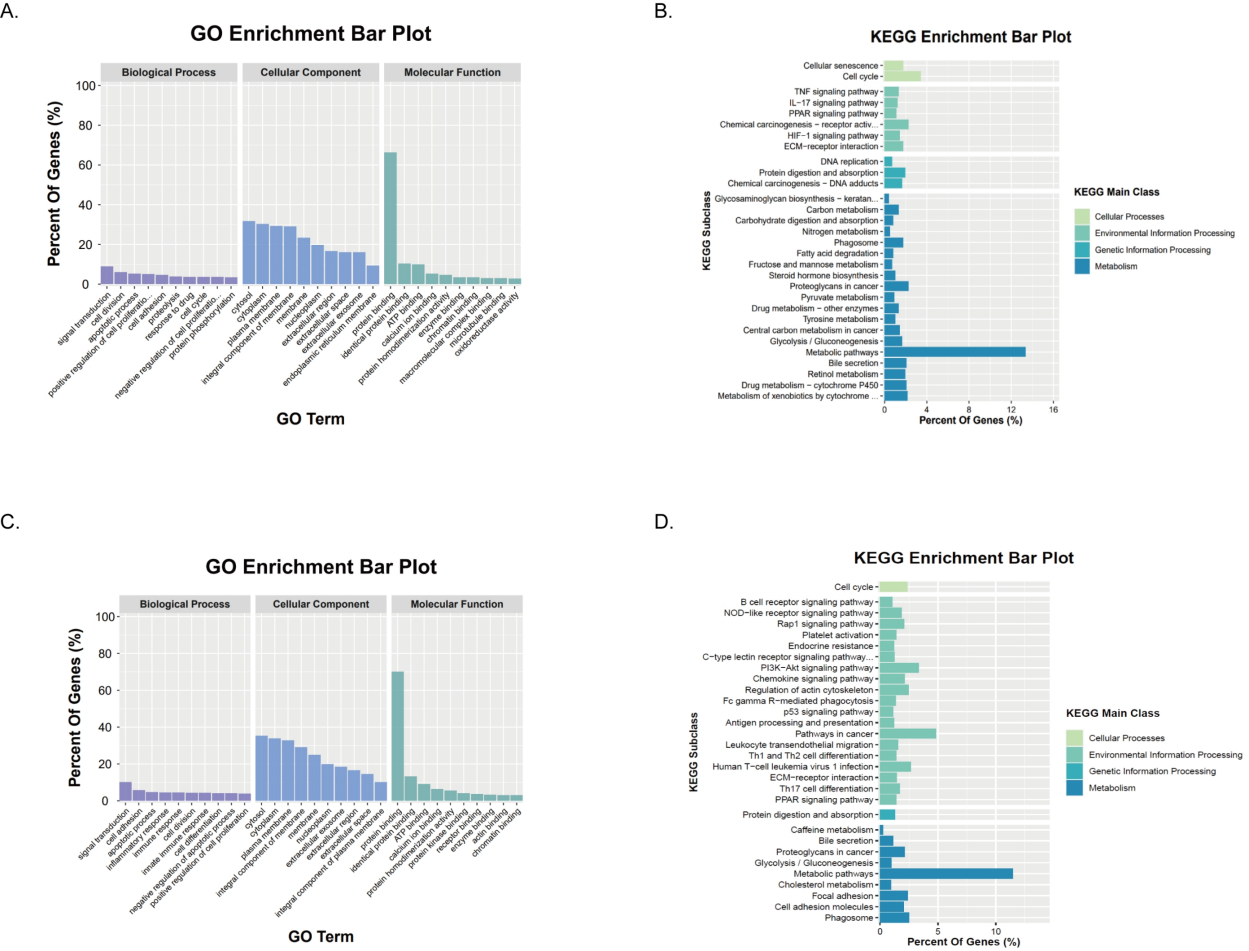
Functional enrichment of the DEGs between risk groups. (A–B) In the TCGA cohort, results of GO and KEGG pathway analysis. (C–D) In the ICGC cohort, results of GO and KEGG pathway analysis.

To elucidate the biological functions and pathways associated with MRRS risk scores, we first used GO enrichment analysis and KEGG pathway analysis for DEGs between high and low-risk groups in the TCGA cohort. The results of GO enrichment analysis showed that in terms of Biological Processes, signal transduction, cell division, and apoptotic process were significantly enriched. In terms of cellular components, cytosol, cytoplasm, and plasma membrane were significantly enriched; In terms of Molecular Function, protein binding, identical protein binding, and ATP binding were significantly enriched (Fig. 7A). KEGG enrichment results showed significant enrichment of metabolic pathways such as drug metabolism-cytochrome P450, retinol metabolism, central carbon metabolism, and tyrosine metabolism. Cell cycle, glycolysis/gluconeogenesis, ECM-receptor interaction, and signaling pathways such as the HIF-1 signaling pathway and PPAR signaling pathway were also enriched (Fig. 7B). In addition, immune-related signaling pathways such as the IL-17 signaling pathway and the TNF signaling pathway were significantly affected (Fig. 7B). Then we performed GO enrichment analysis and KEGG enrichment analysis for DEGs between high and low-risk groups in the ICGC cohort. GO enrichment analysis showed significant enrichment of cell adhesion, cell division, and positive regulation of T cell proliferation and activation pathways (Fig. 7C). KEGG enrichment results showed that in addition to the significant enrichment of metabolic signaling pathways, a variety of immune-related signaling pathways were also significantly enriched, such as Th17 cell differentiation, human T-cell leukemia virus 1 infection, Th1 and Th2 cell differentiation, leukocyte transendothelial migration, antigen processing and presentation, Fc gamma R-mediated phagocytosis, and B cell receptor signaling pathway (Fig. 7D). In addition, consistent with the results of the analysis in the TCGA cohort, the PPAR signaling pathway and PI3K-Akt signaling pathway were also significantly affected (Fig. 7D). Taken together, these findings highlight that HCC patients in the high and low-risk groups may lead differences in tumor malignant characterization through these pathways, ultimately leading to different prognoses of HCC patients.

### Infiltration of immune cells in TME in high- and low-risk populations

**Figure 8:**
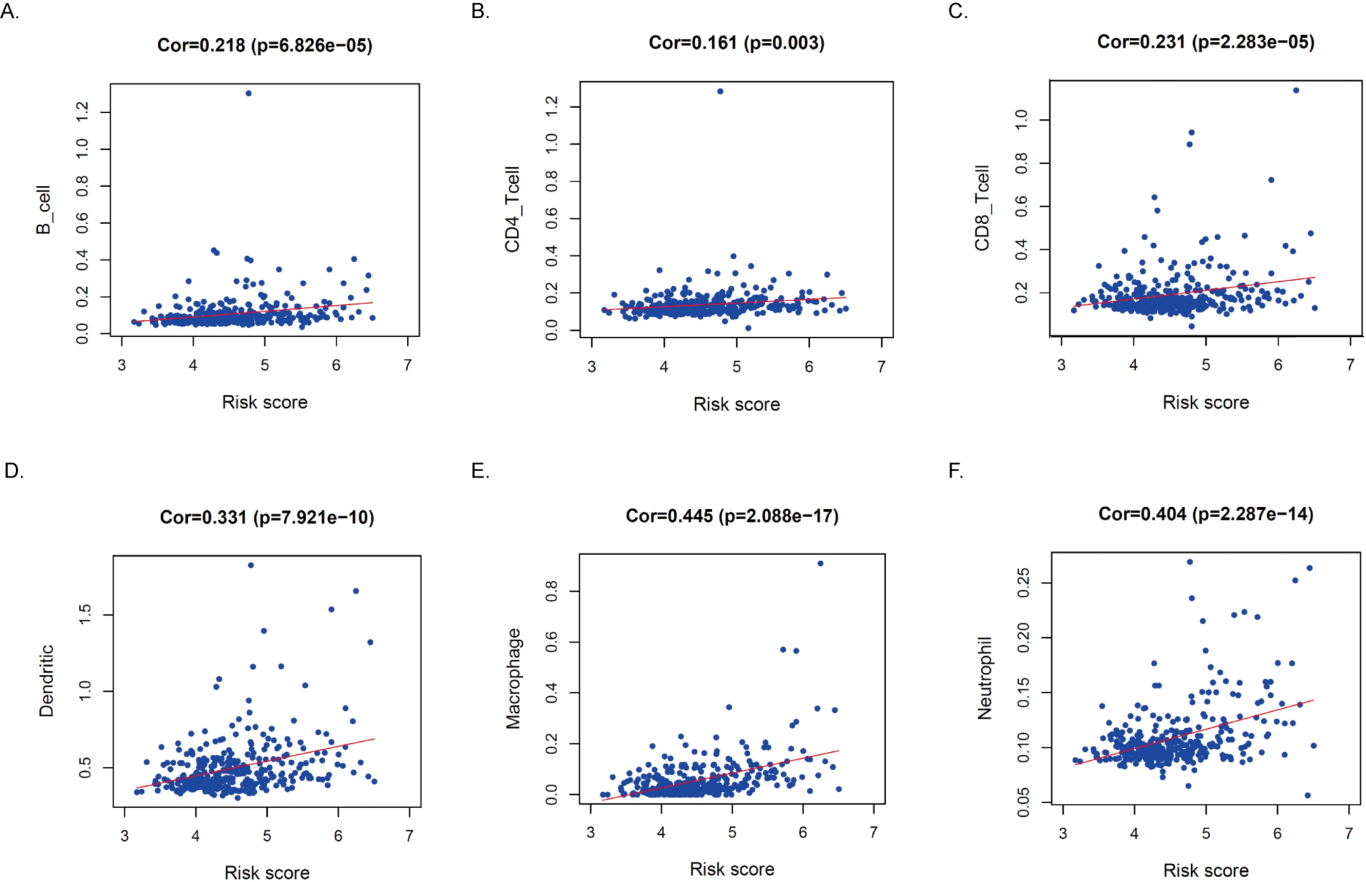
Relationships between the MRRS and infiltration abundances of immune cells. (A–F) The relationship between risk score and six types of immune cells.

GO and KEGG enrichment analysis showed differences in function and pathways between risk groups in ECM-receptor interaction pathways and multiple immune-related signaling pathways. To further explore the correlation between different risk scores and immune status, we further explored the content of immune matrix components in the tumor microenvironment. According to the ESTIMATE algorithm, B cells, CD4+ T cells, CD8+ T cells, DC cells, macrophages, and neutrophil subsets in tumor tissues of high-risk groups were significantly upregulated significantly higher in the high-risk group (*P* < 0.05, Fig. 8), indicating that there are more immune cell components in TME in high-risk HCC patients.

### Downregulation of GOT2 promotes the migration capacity of hepatocellular carcinoma

**Figure 9:**
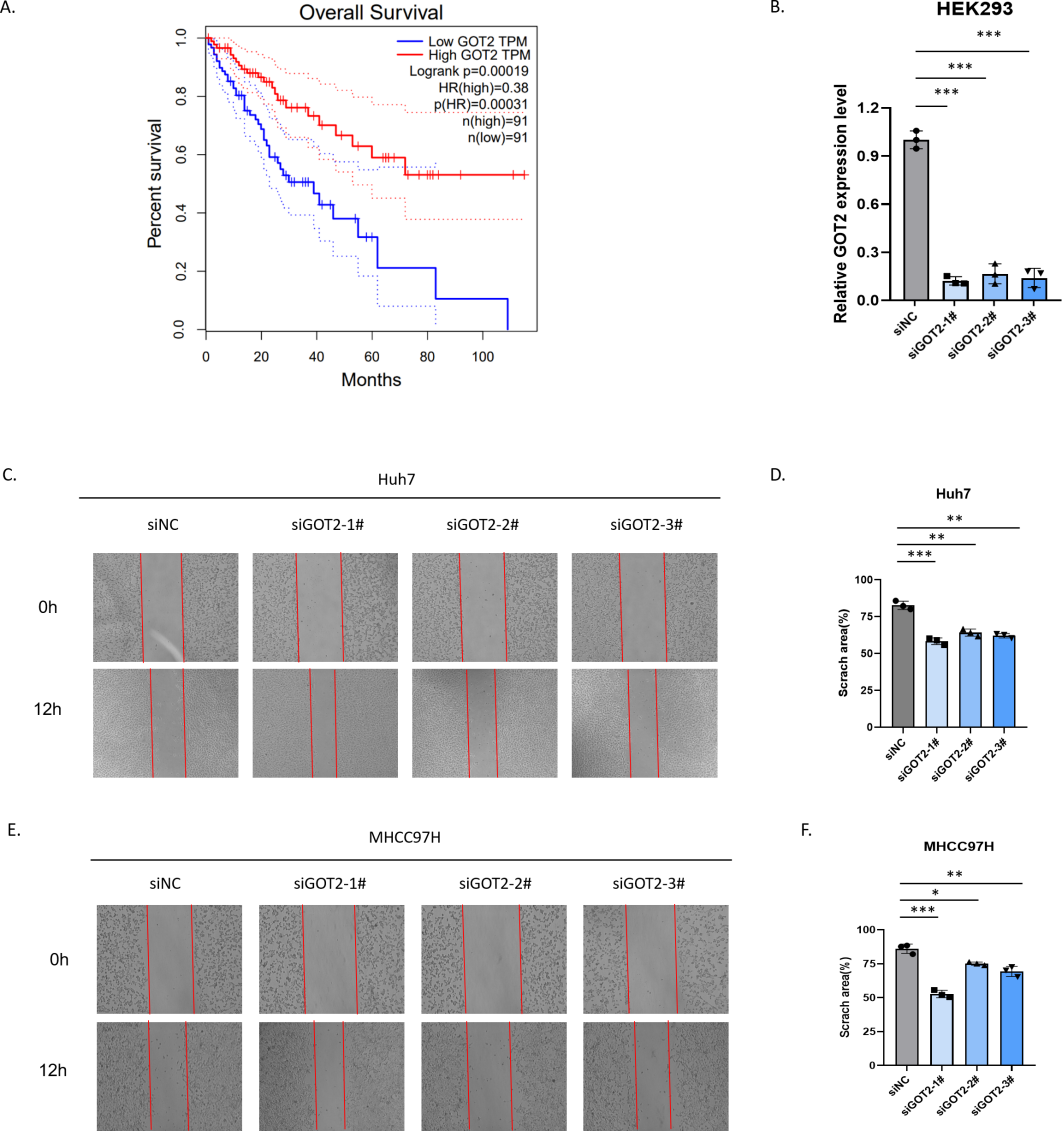
Decreased expression of GOT2 in hepatocellular carcinoma inhibits migration. (A) The Kaplan–Meier plots plot shows the relationship between GOT2 expression levels and patient outcomes. (B) HEK293 cell line was transfected with siNC or siGOT2 and the mRNA expression level of GOT2 was by RT-qPCR. (C–F) Wound healing assays showed that GOT2 reduced cell migration in Huh7 (C–D) and MHCC97H (E–F). Error bar indicates SD of the mean. ^∗^*p* < 0.05, ^∗∗^*p* < 0.01, ^∗∗∗^*p* < 0.001 by biological repeated-measures analysis of variance (*n* = 3).

By analyzing the relationship between prognosis and GOT2 mRNA expression levels in patients with hepatocellular carcinoma in the TCGA database, we found that patients with low GOT2 expression had a worse prognosis in the TCGA database (Fig. 9A). To further explore the biological significance of GOT2 in the progression of hepatocellular carcinoma, we first transfected siRNAs targeting GOT2 (siGOT2) or negative control siRNA (siNC) in HEK293 cells and verified their knockdown efficiency by RT-qPCR (Fig. 9B). The wound healing assay was used to assess the effect of GOT2 on the migration of hepatocellular carcinoma cells (Huh7 and MHCC97H). As shown in Figs. 9C–9F, down-regulation of GOT2 significantly inhibited migration in both cell lines compared to the control group. Together, our findings provide new insights into the role of GOT2 in influencing malignant phenotypes by regulating migration in hepatocellular carcinoma cells.

## Discussion

HCC is an extremely common malignancy worldwide, it is important to develop reliable prognostic indicators for HCC patients. In this study, we established 14 genetic biomarkers as new prognostic models of MRRS and analyzed their ability to predict prognosis in HCC patients. The prognostic performance of the model is verified by the survival curve and ROC curve, and the results show that the model has good predictive performance. Its prediction efficiency was verified in the ICGC cohort. MRRS is a good independent indicator for predicting the prognosis of HCC patients in both TCGA and ICGC cohorts. Our prognostic model can help predict the prognosis of HCC patients clinically, thereby recommending better treatment measures for high-risk HCC patients.

GO and KEGG analysis showed that cell proliferation signals were significantly altered in patients in the high-risk group, suggesting that metabolism significantly affects cell proliferation, and cell proliferation may be the cause of poor prognosis in HCC patients. The mechanisms involved may be related to the PPAR signaling pathway, and PI3K-Akt signaling pathway, so PPAR inhibitors and PI3K-Akt inhibitors can be tried in high-risk patients to have a better prognosis for HCC patients. Functional analysis showed that immune signals were widely involved in tumor processes in high-risk patients, and more immune cells were infiltrated in the tumor microenvironment of high-risk patients. Our results are consistent with previous studies, and that increased expression of tumor blasts such as regulatory T cells, tumor-associated macrophages, tumor-associated neutrophils, and myeloid-derived suppressor cells in most cancers generally predicts worse outcomes (Senovilla et al., 2012). TME is generally divided into three categories (Chen & Mellman, 2017; Lanitis et al., 2017): (1) immune-inflamed: immune cells exist near tumor cells, (2) immune-excluded: immune cells exist around the stroma but do not penetrate the tumor, (3) immune-desert: lack of immune cell infiltration. In the current study, we reasonably speculate that tumors in patients with high-risk HCC may be Immune-excluded tumors (IETs). In this case, although TME shows abundant immune cell infiltration, cytotoxic T lymphocytes (CTL) cannot effectively infiltrate the tumor and exert a killing effect. This suggests that immunotherapy alone in high-risk patients may be less effective in patients. At present, the possibility that high-risk patients are immune-inflamed cannot be ruled out, and further evaluation of immunotherapy responses between different patients is needed to obtain better treatment outcomes. High-risk patients with immune inflammation should be aggressively treated with immunotherapy to achieve better clinical outcomes. This strategy may also be used in other cancer patients to seek better treatment targets and carry out precise treatment of cancer.

Although the mechanism of metabolism in tumors has become an increasingly popular area of research in recent years, the potential regulation between tumor immunity and metabolism still needs more research. Different states of tumor-associated macrophages (TAMs) are able to adapt to the tumor microenvironment by altering metabolism. It can inhibit the differentiation of M2-type TAMs by inhibiting the metabolism pathway (Van den Bossche et al., 2016; Divakaruni et al., 2018). Cancer-related MDSCs, whose main energy supply mode is converted from glycolysis to FAO. Increased fatty acid uptake and higher expression of key enzymes, which in turn upregulated the FAO rate necessary to produce immunosuppressive ARG1 (Hossain et al., 2015). Cytokines that drive the expansion of MDSCs. Fatty acid transporters (FATPs), as long-chain fatty acid transporters, upregulate and control the inhibitory activity of MDSCs on tumors in tumor-derived MDSCs (Veglia et al., 2019). This evidence suggests that fatty acid metabolism targeting MDSCs and M2 TAMs may be an important means of enhancing the efficacy of cancer immunotherapy. In this study, antigen presentation signaling pathways differed significantly between different risk groups. One possible hypothesis is that differences in mediators in tumor tissues attract antigen-presenting cells (APCs) to the site of tumor cells (Friedmann Angeli, Krysko & Conrad, 2019). Our study shows that many immune-related biological processes and pathways are enriched among HCC patients with different metabolism risk groups. Therefore, it is reasonable to assume that metabolism in tumor tissues in HCC patients is closely related to tumor immunity.

Recent studies have found that amino acid metabolism (Bertero et al., 2019; Ericksen et al., 2019) and tricarboxylic acid metabolism (Nie et al., 2020) play an important role in tumor development and cancer drug resistance (Yoo & Han, 2022; Ma et al., 2018). As a member of glutamate-oxaloacetate aminotransferase, a recent study showed that GOT2 expression levels affect metabolism (Wang et al., 2018), suggesting that GOT2 may be an important regulator of cellular metabolism. In our study, the prognostic curve confirms that GOT2 plays an important role in predicting survival in patients with hepatocellular carcinoma. To gain a deeper understanding of the mechanism by which GOT2 affects tumor progression, wound healing assays are performed. These findings suggest that GOT2 has a significant activating effect on the migration ability of hepatocellular carcinoma.

In summary, our study defines a new prognostic model MRRS constructed from 14 MRGs. The model was shown to be independently correlated with OS in the derivation and validation cohorts, providing insights for predicting HCC prognosis. The study had several limitations. First, our prognostic model was constructed and validated with retrospective data from a public database. More forward-looking, real-world data are needed to validate its clinical utility. Secondly, since the model focuses on the evaluation ability of metabolism in HCC patients, a variety of indicators should be included in the comprehensive judgment of clinical decision-making. For example, it should be emphasized that the responsiveness of HCC patients in the high-risk score group to immunotherapy needs more research to elucidate.

## Supplemental Information

10.7717/peerj.16335/supp-1 Supplemental Information 1 Risk Score The penalty parameter (*λ* ) of the model is determined by tenfold cross-validation following the minimum criterion (*i.e.*, the *λ* value corresponding to the lowest partial likelihood bias). Subsequently, the patient’s risk score is calculated based on gene expression and the corresponding Cox regression coefficient as follows: score= sum (expression of each gene × corresponding coefficient)

10.7717/peerj.16335/supp-2 Supplemental Information 2 Raw data of Fig. 9B

10.7717/peerj.16335/supp-3 Supplemental Information 3 Raw data of Figs. 9C–9D

10.7717/peerj.16335/supp-4 Supplemental Information 4 Numeric data of Figs. 9D–9F

10.7717/peerj.16335/supp-5 Supplemental Information 5 Raw data of Figs. 9E–9F

## Additional Information and Declarations

### Data Availability

The following information was supplied regarding data availability: The raw measurements are available in the Supplementary Files.
