# Supplementary material for: A novel metabolism-related gene signature in patients with hepatocellular carcinoma
Source: PeerJ. 2023 Nov 9;11:e16335. doi: 10.7717/peerj.16335 (PMC10640845; doi:10.7717/peerj.16335)
Supplement: Supplemental Information 1 — The penalty parameter (λ ) of the model is determined by tenfold cross-validation following the minimum criterion (i.e., the λ value corresponding to the lowest partial likelihood bias). Subsequently, the patient’s risk score is calculated based on gene expression and the corresponding Cox regression coefficient as follows: score= sum (expression of each gene × corresponding coefficient) [file peerj-11-16335-s001.docx]

| risk score = (0.0875 * DLAT expression) + (0.2953 * SEPHS1 expression) + (-0.1116 * ACADS expression) + (0.1978 * UCK2 expression) + (-0.0143 * GOT2 expression) + (-0.0295 * ADH4 expression) + (-0.3244 * LDHA expression) + (0.0520 * ME1 expression) + (0.0105 * TXNRD1 expression) + (0.0433 * B4GALT2 expression) + (0.1975* AK2 expression) + (0.1783* PTDSS2 expression) + (-0.023 * CSAD expression) + (0.0207 * AMD1 expression) |
| --- |
