# Supplementary figures and images for: A novel metabolism-related gene signature in patients with hepatocellular carcinoma
Source: PeerJ. 2023 Nov 9;11:e16335. doi: 10.7717/peerj.16335 (PMC10640845; doi:10.7717/peerj.16335)

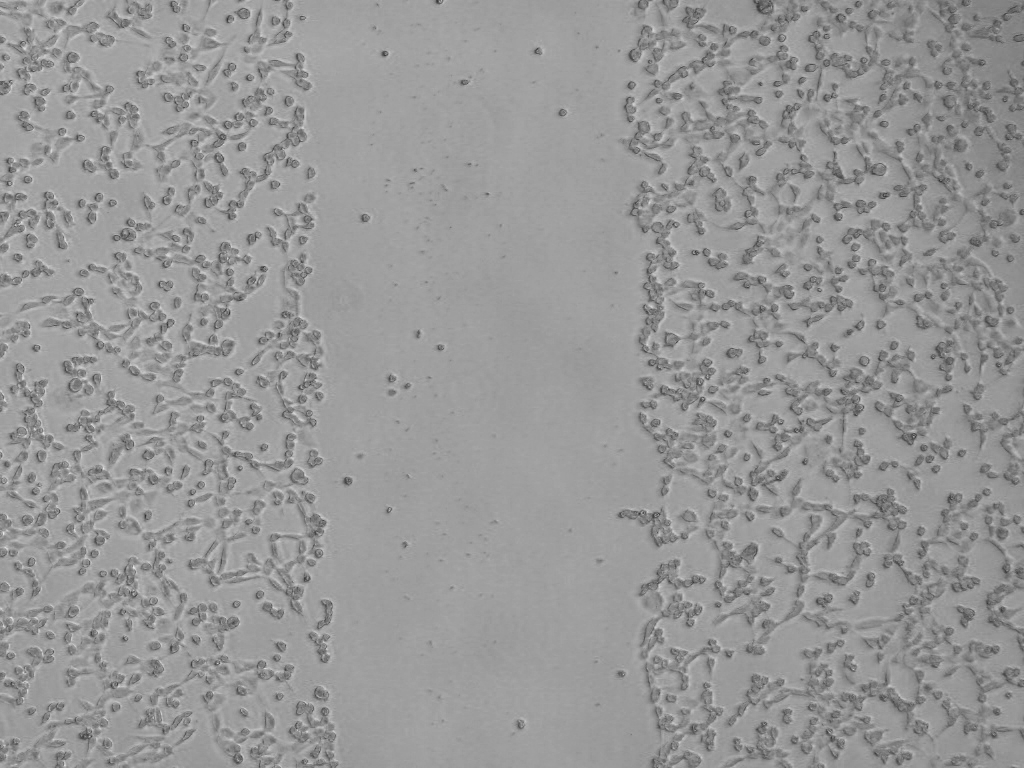

Supplement: Supplemental Information 3 [file peerj-11-16335-s003.zip › Raw data of Figures 9 C-D/peerj-86802-raw_data_of_figure9C-D-1.tiff]

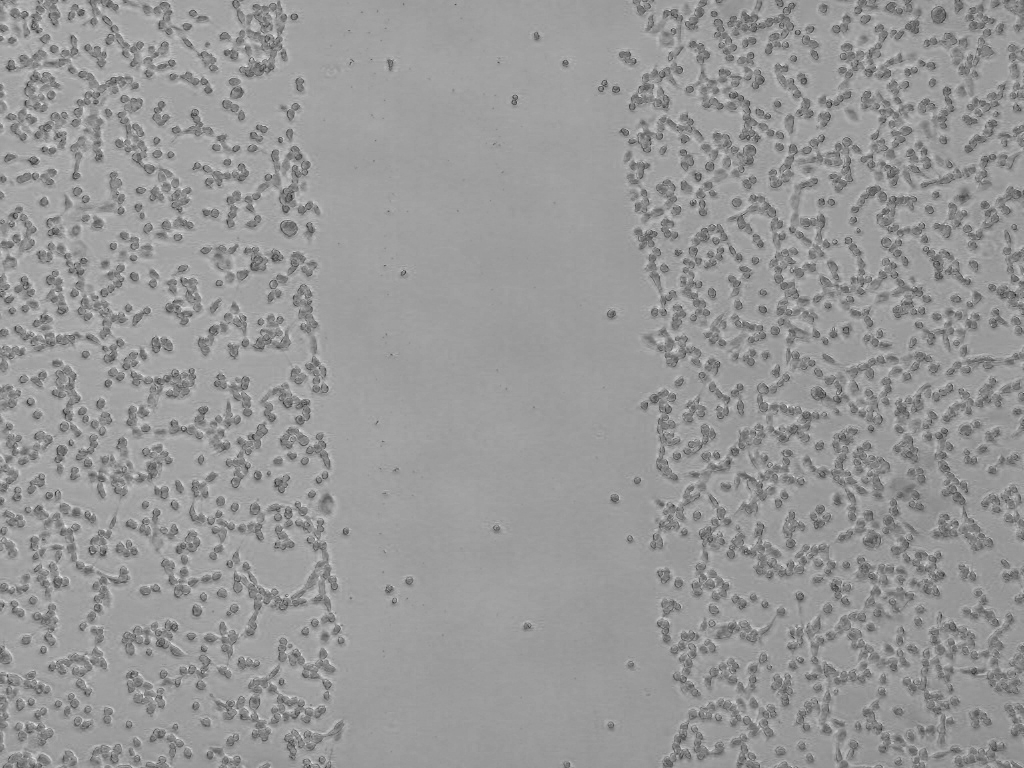

Supplement: Supplemental Information 3 [file peerj-11-16335-s003.zip › Raw data of Figures 9 C-D/peerj-86802-raw_data_of_figure9C-D-10.tiff]

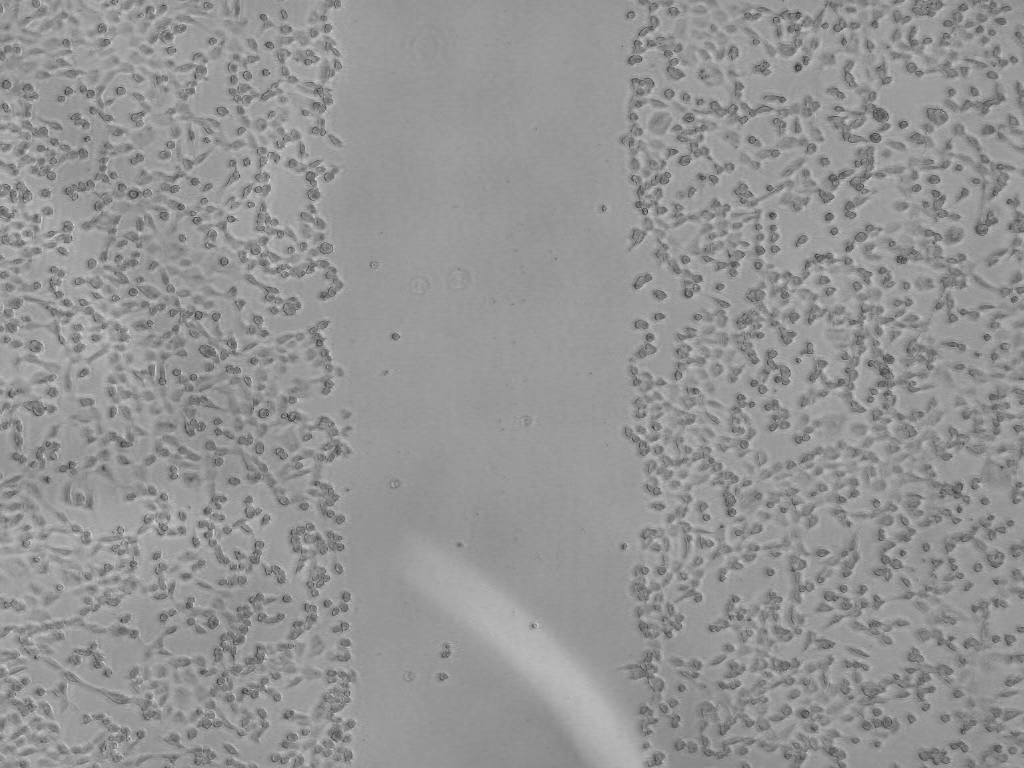

Supplement: Supplemental Information 3 [file peerj-11-16335-s003.zip › Raw data of Figures 9 C-D/peerj-86802-raw_data_of_figure9C-D-11.tiff]

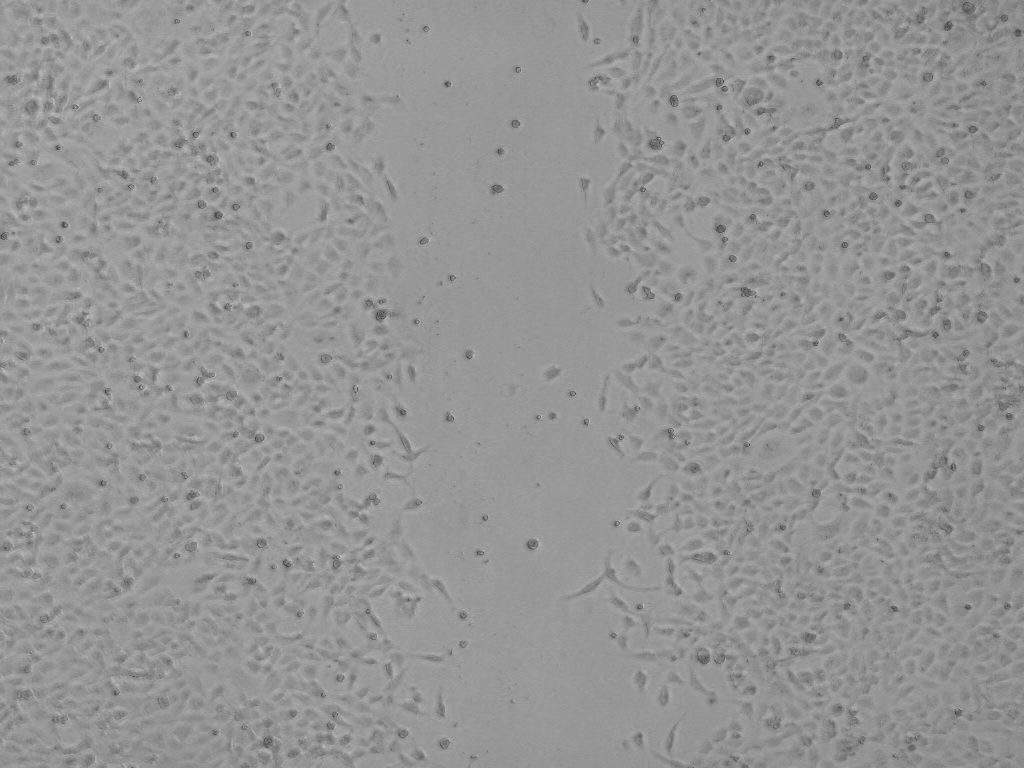

Supplement: Supplemental Information 3 [file peerj-11-16335-s003.zip › Raw data of Figures 9 C-D/peerj-86802-raw_data_of_figure9C-D-12.tiff]

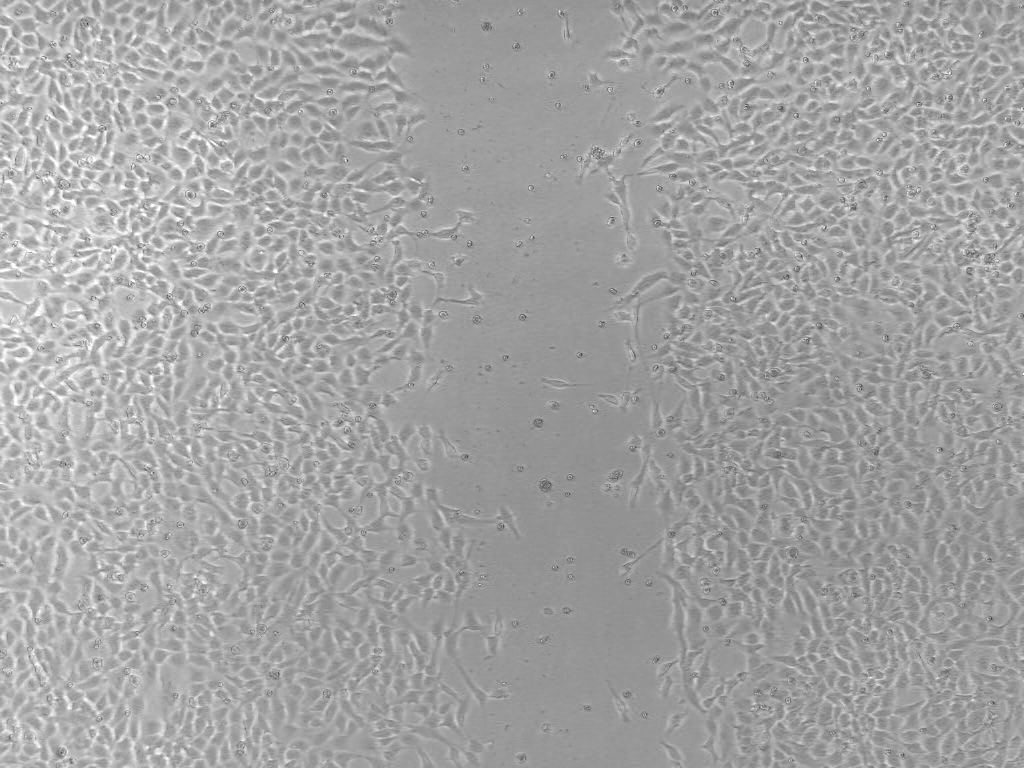

Supplement: Supplemental Information 3 [file peerj-11-16335-s003.zip › Raw data of Figures 9 C-D/peerj-86802-raw_data_of_figure9C-D-13.tiff]

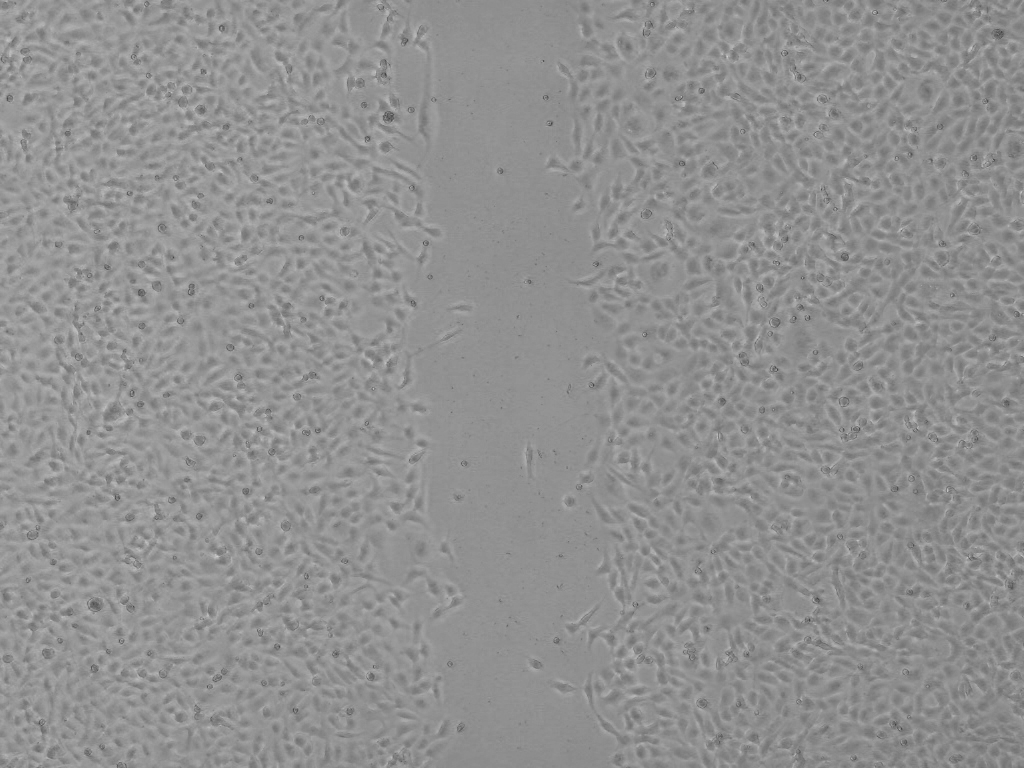

Supplement: Supplemental Information 3 [file peerj-11-16335-s003.zip › Raw data of Figures 9 C-D/peerj-86802-raw_data_of_figure9C-D-14.tiff]

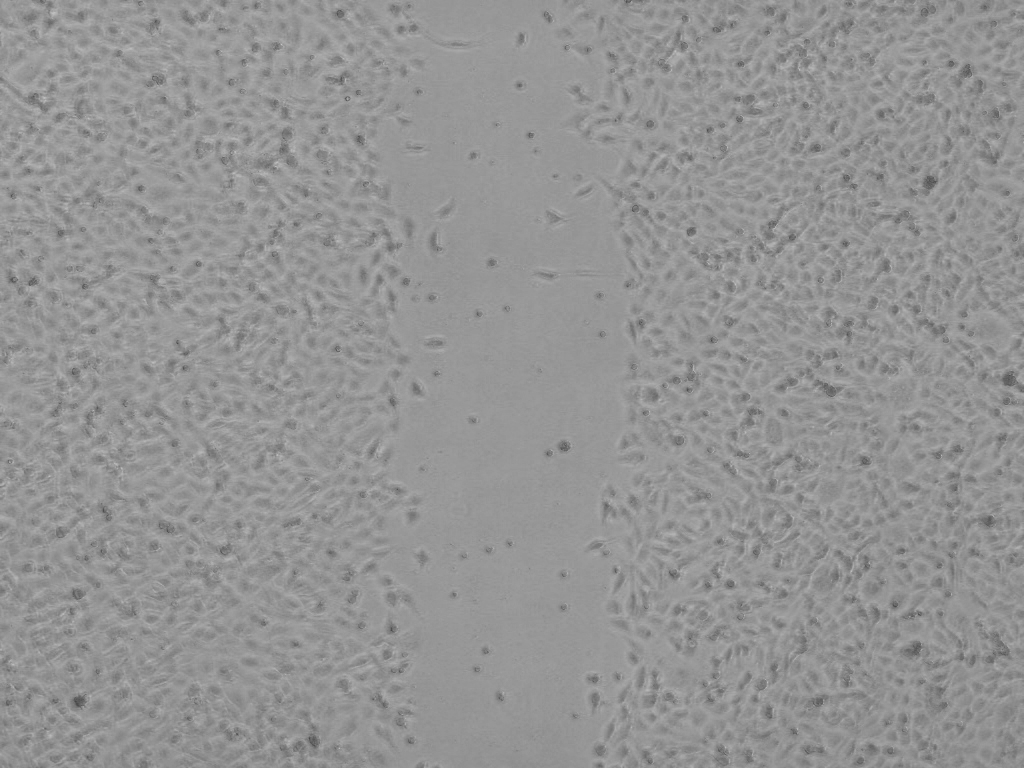

Supplement: Supplemental Information 3 [file peerj-11-16335-s003.zip › Raw data of Figures 9 C-D/peerj-86802-raw_data_of_figure9C-D-15.tiff]

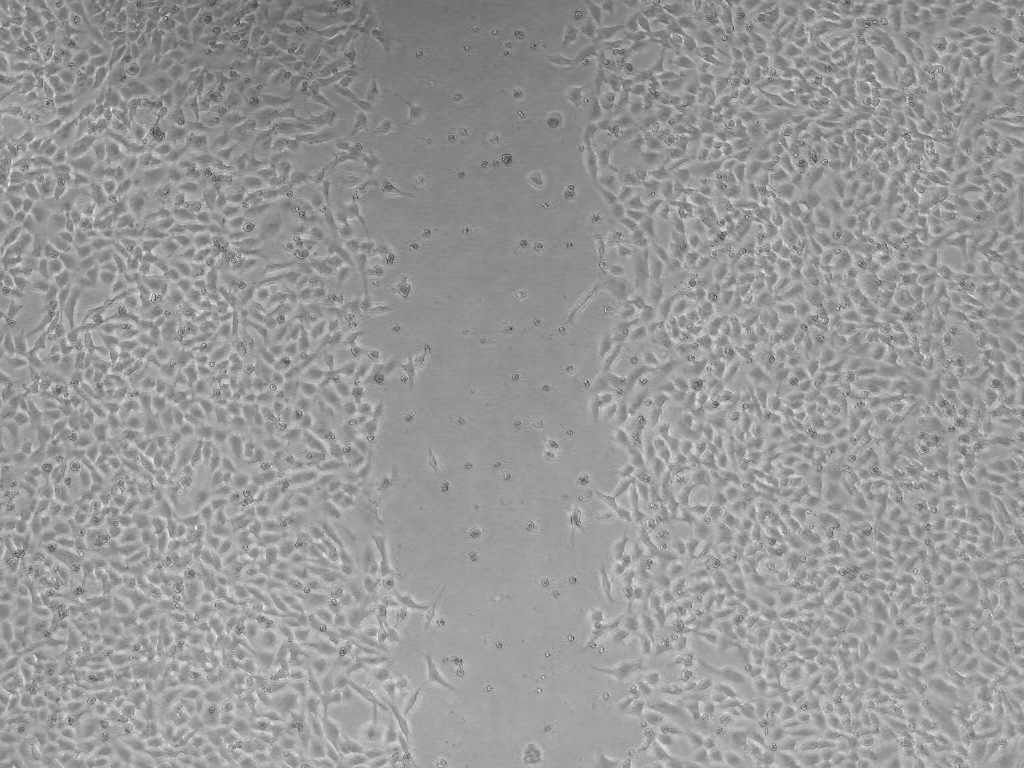

Supplement: Supplemental Information 3 [file peerj-11-16335-s003.zip › Raw data of Figures 9 C-D/peerj-86802-raw_data_of_figure9C-D-16.tiff]

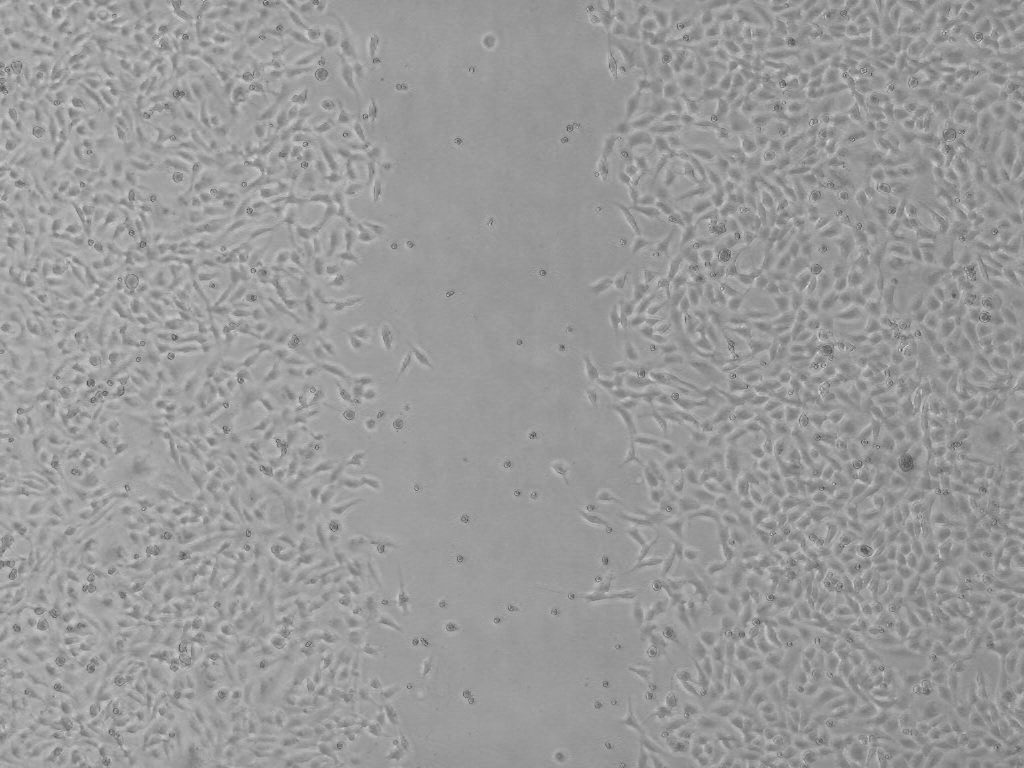

Supplement: Supplemental Information 3 [file peerj-11-16335-s003.zip › Raw data of Figures 9 C-D/peerj-86802-raw_data_of_figure9C-D-17.tiff]

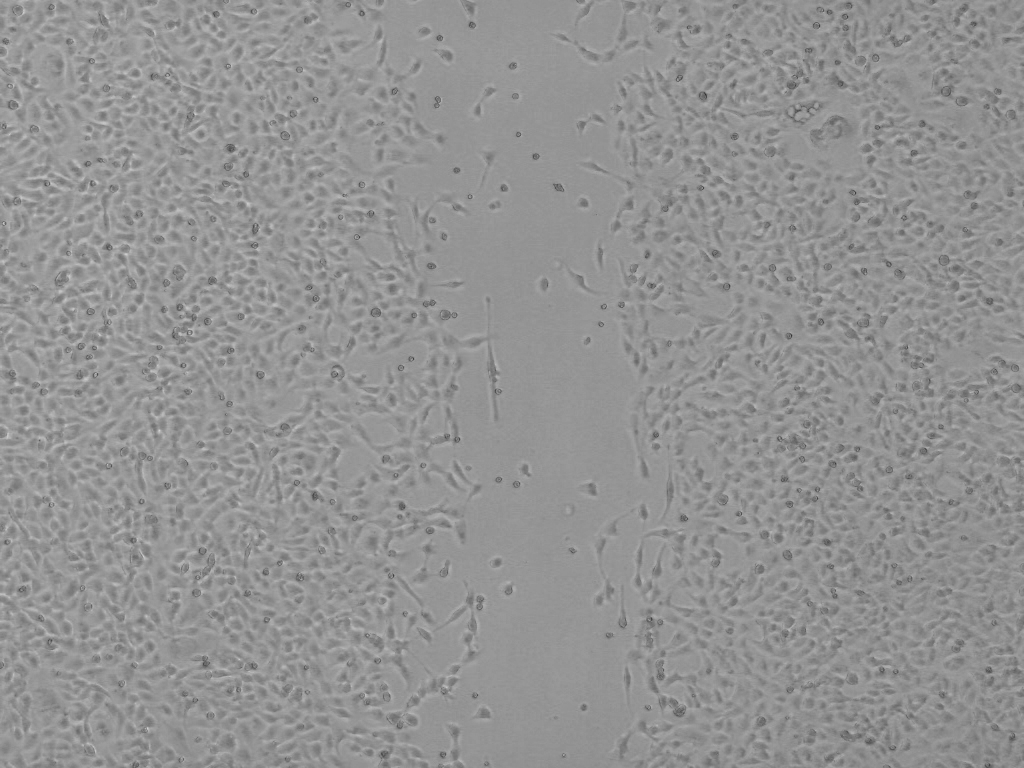

Supplement: Supplemental Information 3 [file peerj-11-16335-s003.zip › Raw data of Figures 9 C-D/peerj-86802-raw_data_of_figure9C-D-18.tiff]

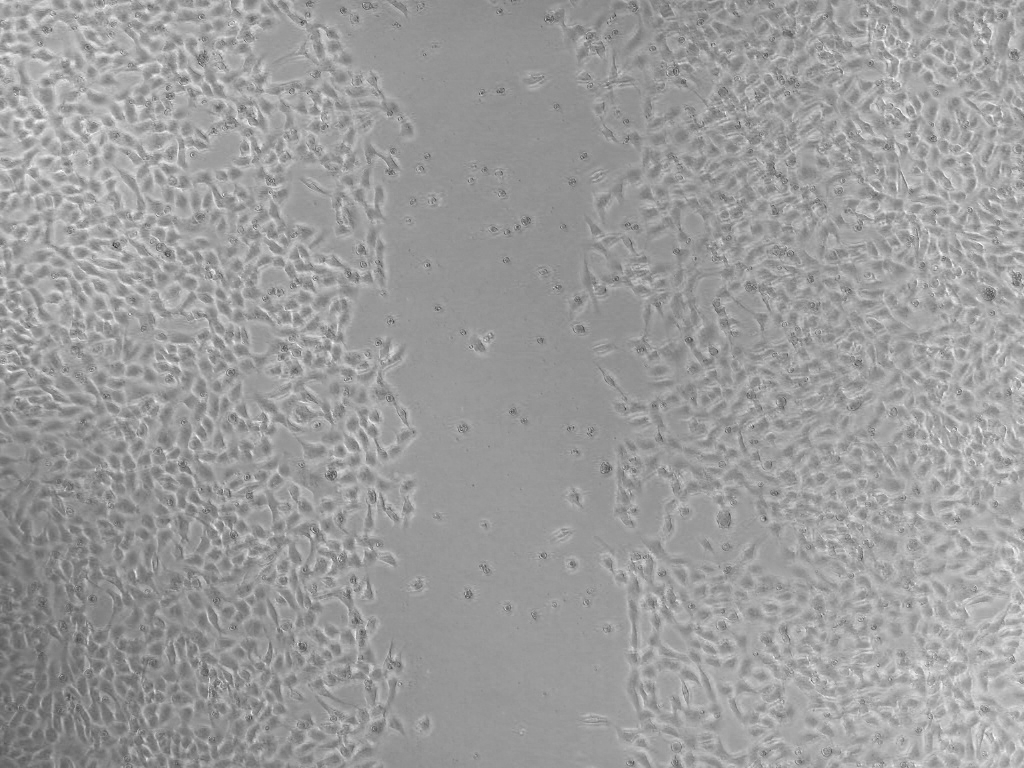

Supplement: Supplemental Information 3 [file peerj-11-16335-s003.zip › Raw data of Figures 9 C-D/peerj-86802-raw_data_of_figure9C-D-19.tiff]

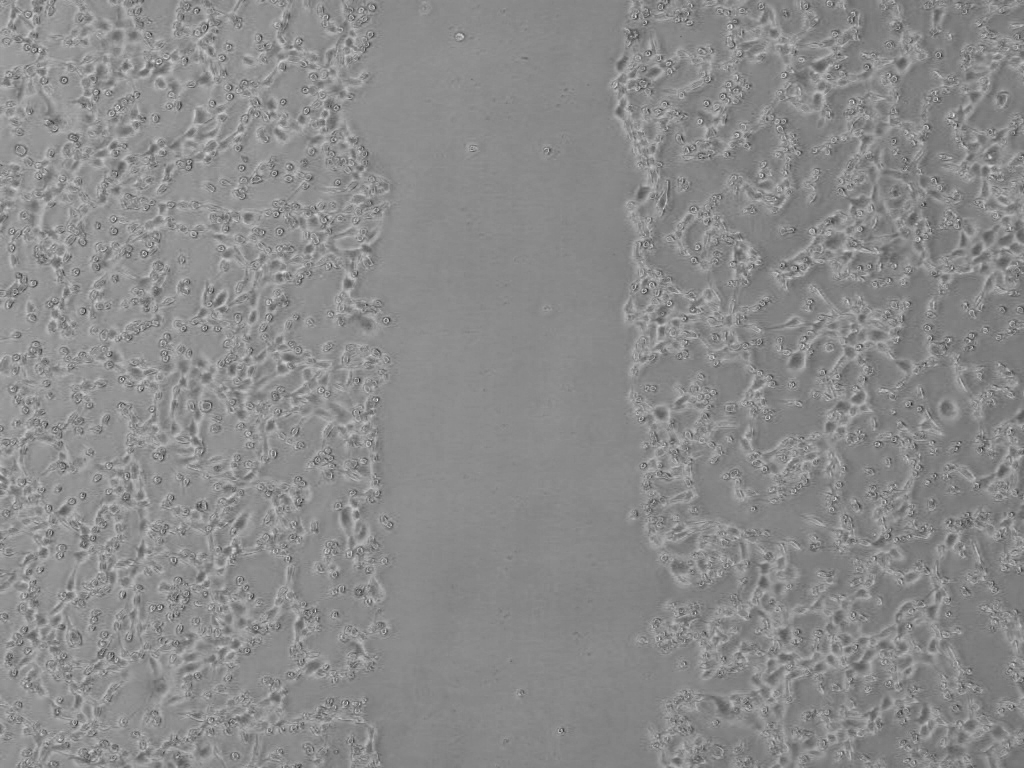

Supplement: Supplemental Information 3 [file peerj-11-16335-s003.zip › Raw data of Figures 9 C-D/peerj-86802-raw_data_of_figure9C-D-2.tiff]

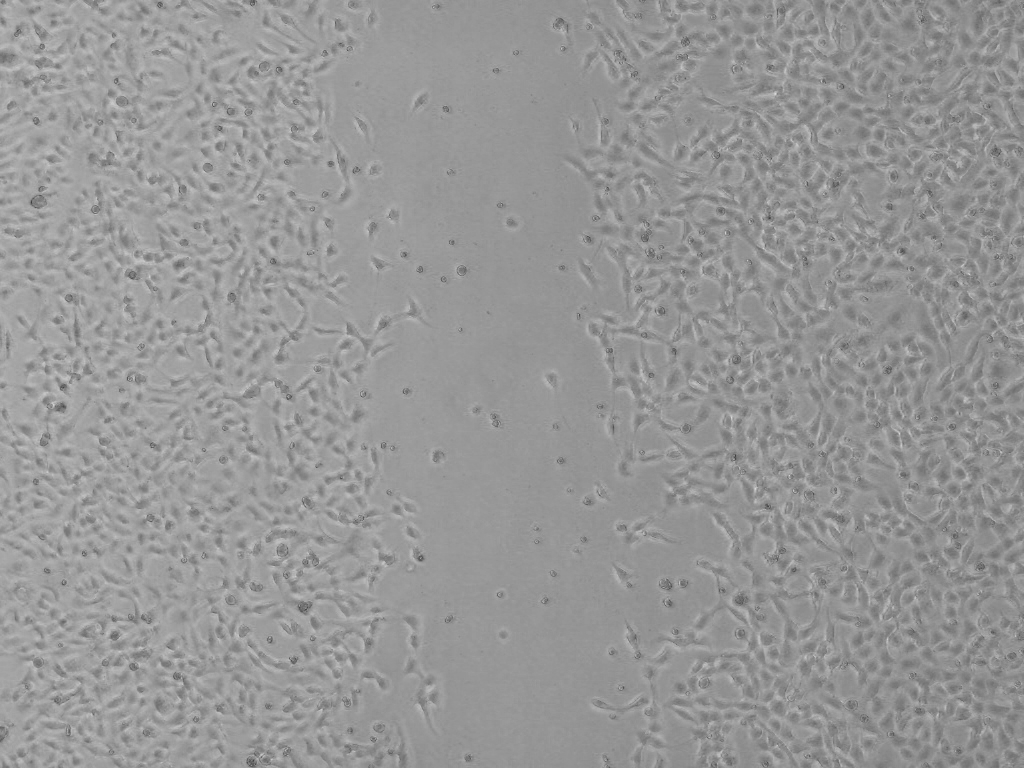

Supplement: Supplemental Information 3 [file peerj-11-16335-s003.zip › Raw data of Figures 9 C-D/peerj-86802-raw_data_of_figure9C-D-20.tiff]

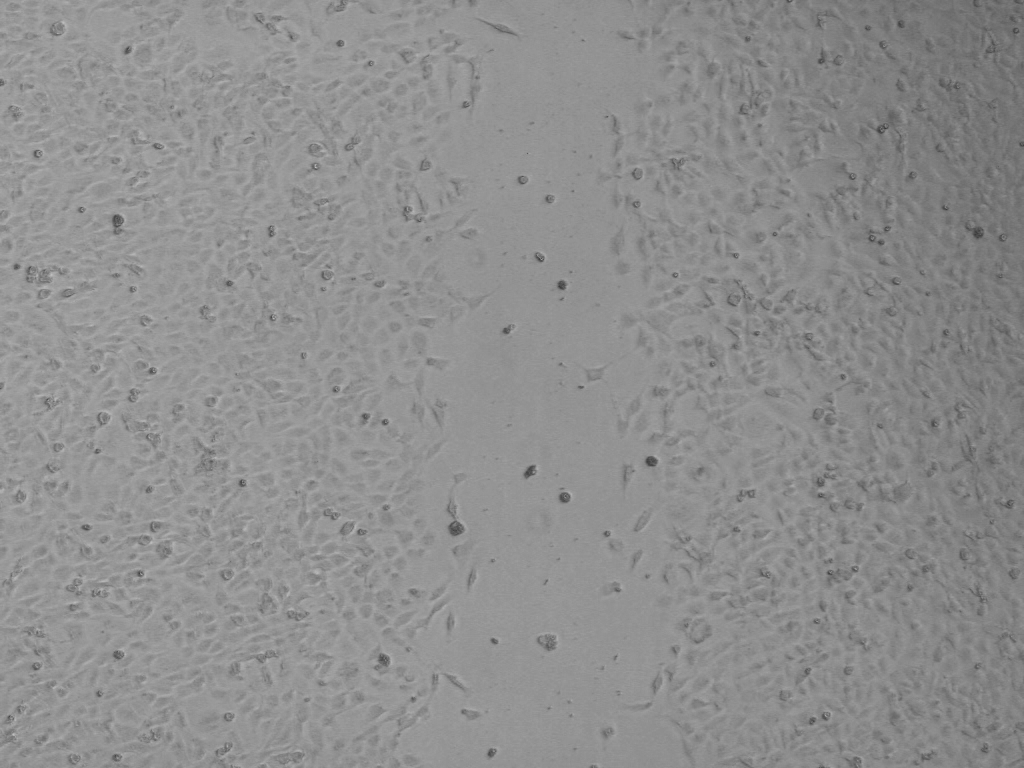

Supplement: Supplemental Information 3 [file peerj-11-16335-s003.zip › Raw data of Figures 9 C-D/peerj-86802-raw_data_of_figure9C-D-21.tiff]

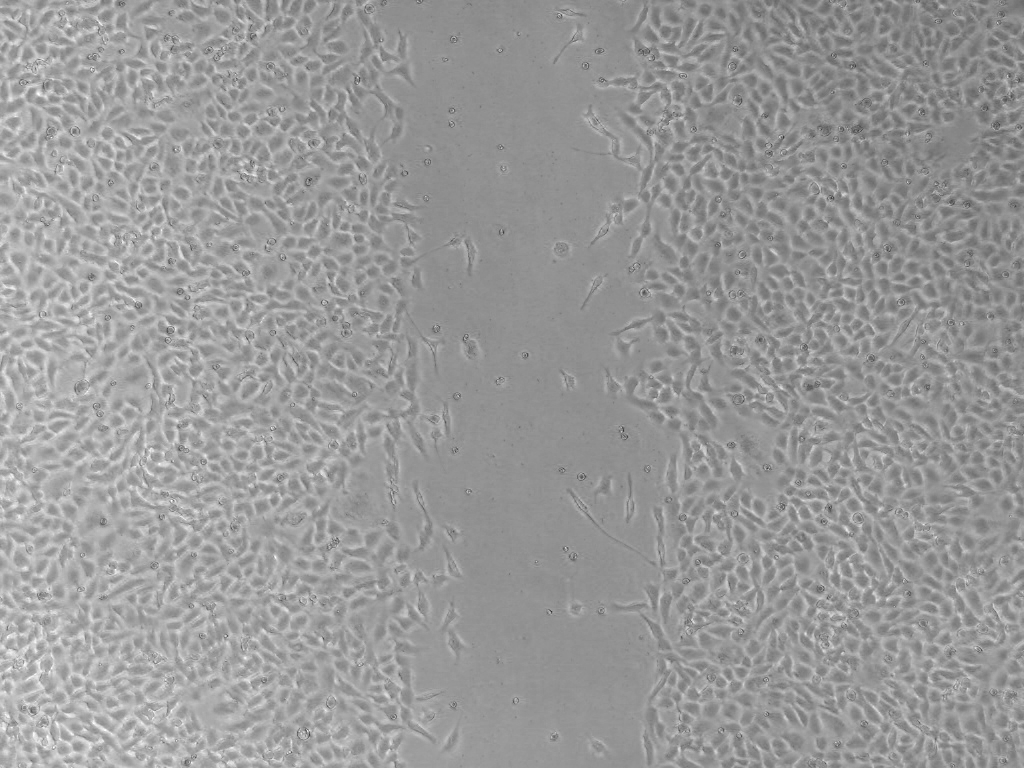

Supplement: Supplemental Information 3 [file peerj-11-16335-s003.zip › Raw data of Figures 9 C-D/peerj-86802-raw_data_of_figure9C-D-22.tiff]

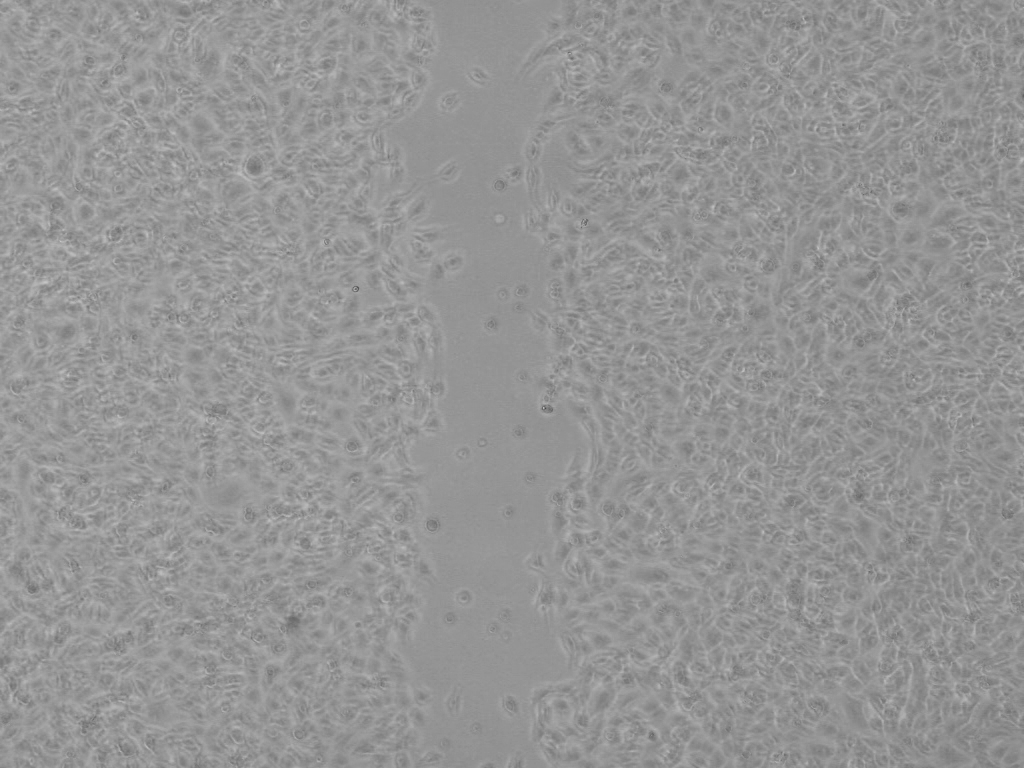

Supplement: Supplemental Information 3 [file peerj-11-16335-s003.zip › Raw data of Figures 9 C-D/peerj-86802-raw_data_of_figure9C-D-23.tiff]

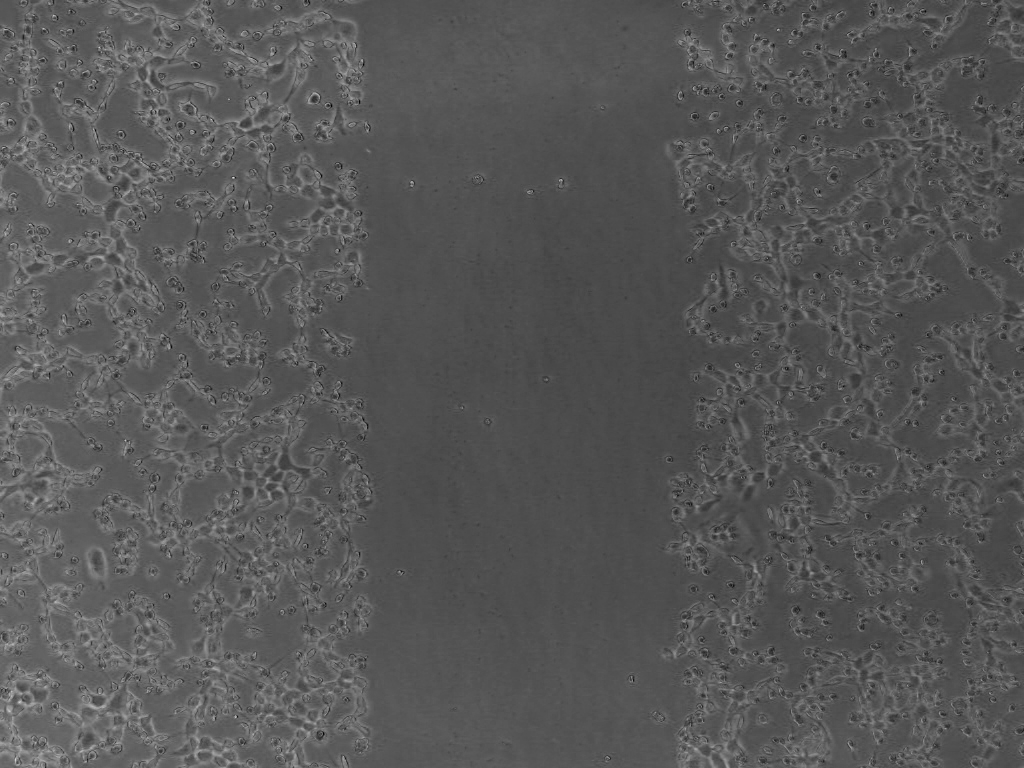

Supplement: Supplemental Information 3 [file peerj-11-16335-s003.zip › Raw data of Figures 9 C-D/peerj-86802-raw_data_of_figure9C-D-3.tiff]

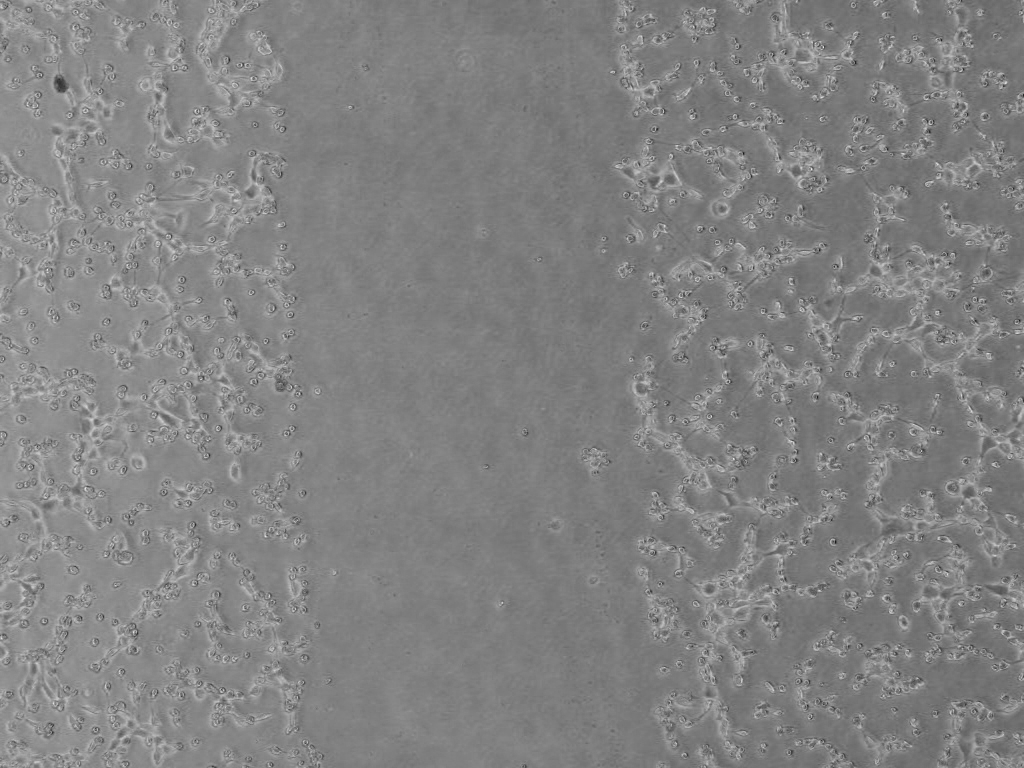

Supplement: Supplemental Information 3 [file peerj-11-16335-s003.zip › Raw data of Figures 9 C-D/peerj-86802-raw_data_of_figure9C-D-4.tiff]

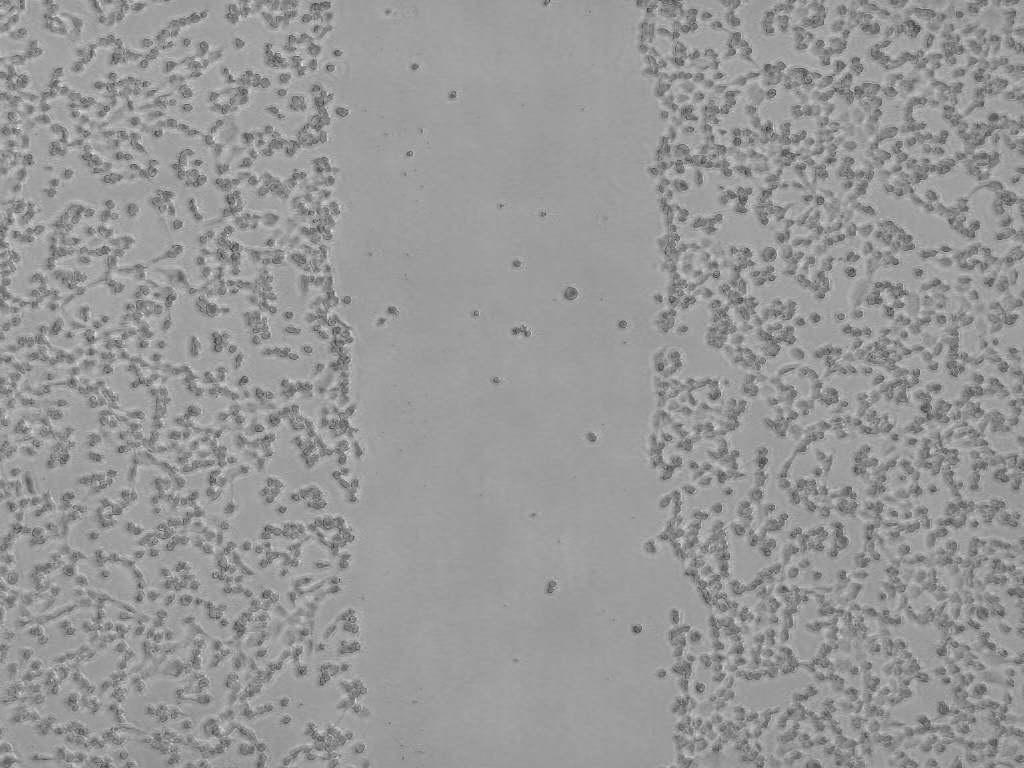

Supplement: Supplemental Information 3 [file peerj-11-16335-s003.zip › Raw data of Figures 9 C-D/peerj-86802-raw_data_of_figure9C-D-5.tiff]

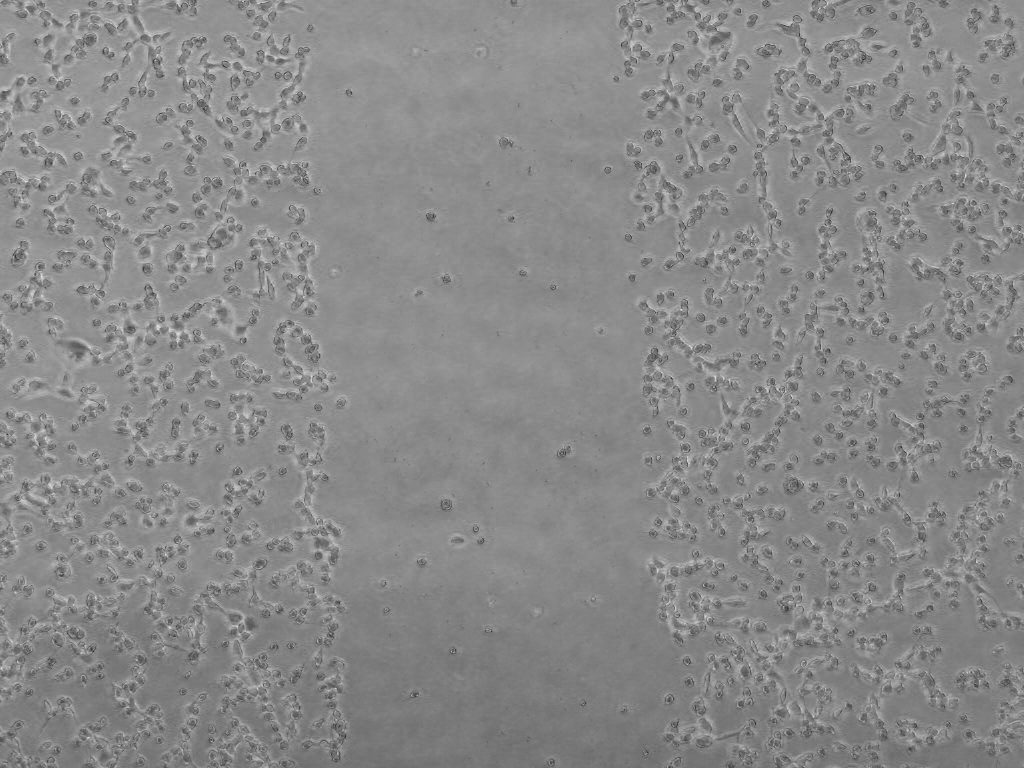

Supplement: Supplemental Information 3 [file peerj-11-16335-s003.zip › Raw data of Figures 9 C-D/peerj-86802-raw_data_of_figure9C-D-6.tiff]

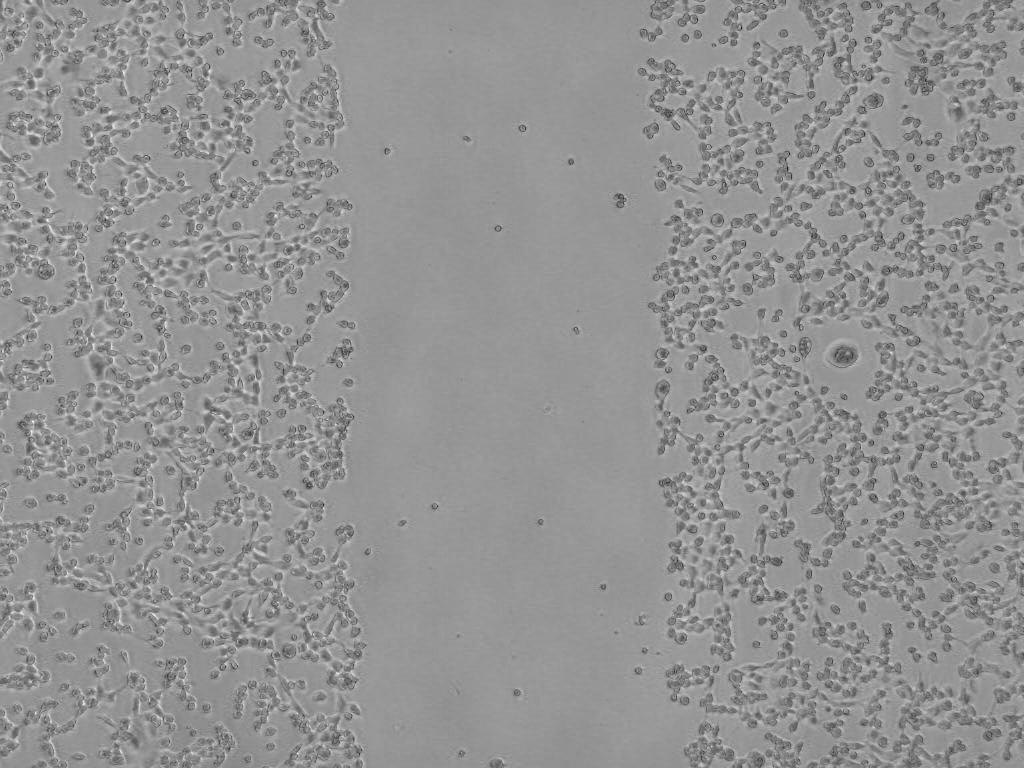

Supplement: Supplemental Information 3 [file peerj-11-16335-s003.zip › Raw data of Figures 9 C-D/peerj-86802-raw_data_of_figure9C-D-7.tiff]

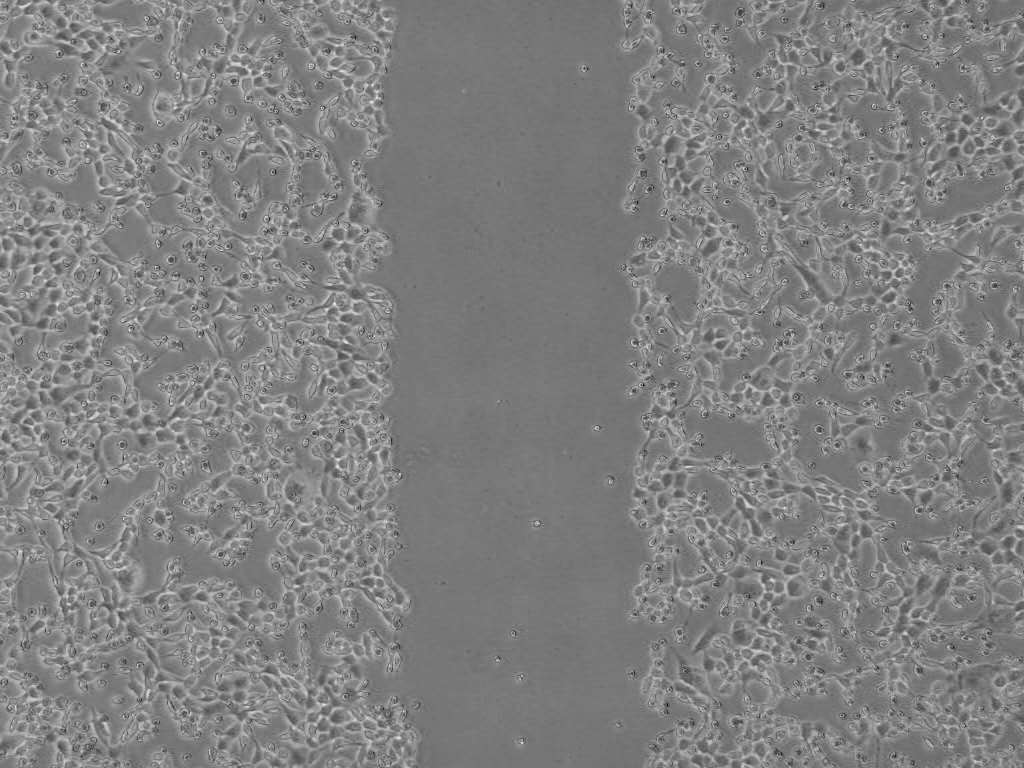

Supplement: Supplemental Information 3 [file peerj-11-16335-s003.zip › Raw data of Figures 9 C-D/peerj-86802-raw_data_of_figure9C-D-8.tiff]

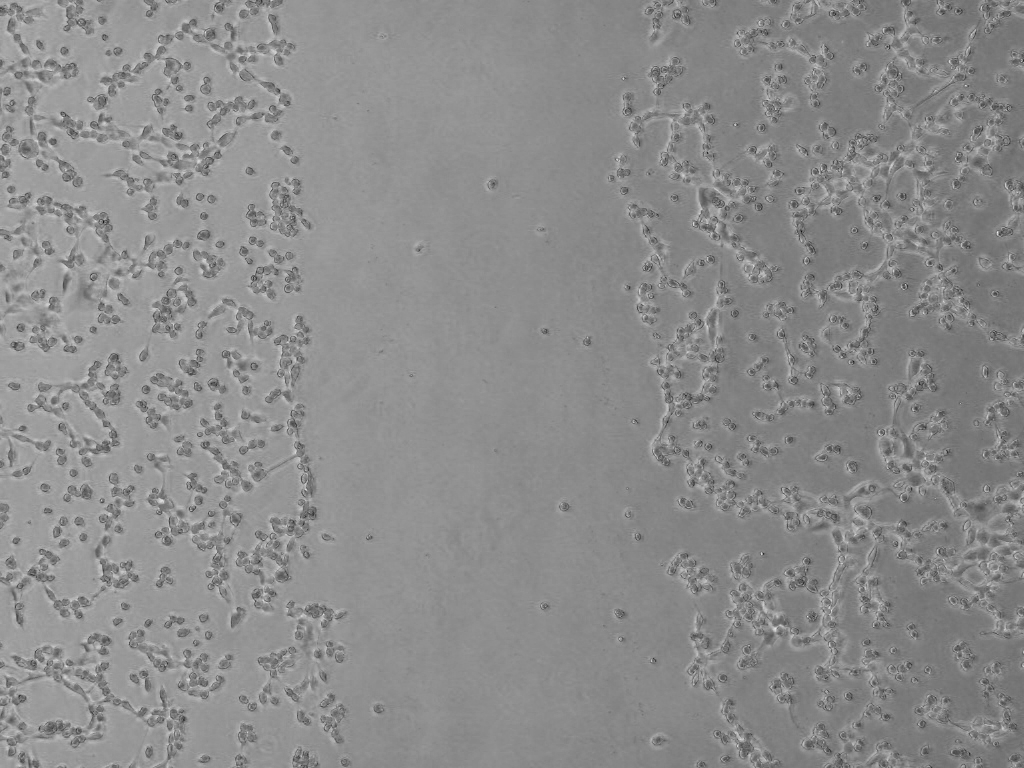

Supplement: Supplemental Information 3 [file peerj-11-16335-s003.zip › Raw data of Figures 9 C-D/peerj-86802-raw_data_of_figure9C-D-9.tiff]

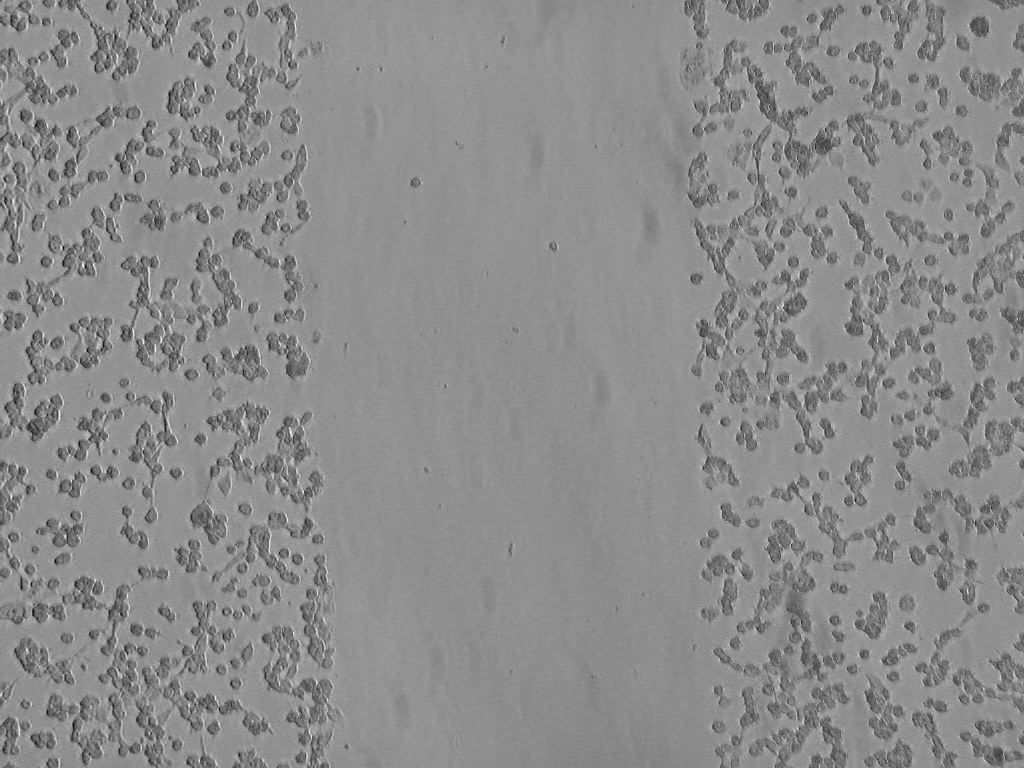

Supplement: Supplemental Information 5 [file peerj-11-16335-s005.zip › Raw data of Figures 9 E-F/peerj-86802-raw_data_of_figure9E-F1.tiff]

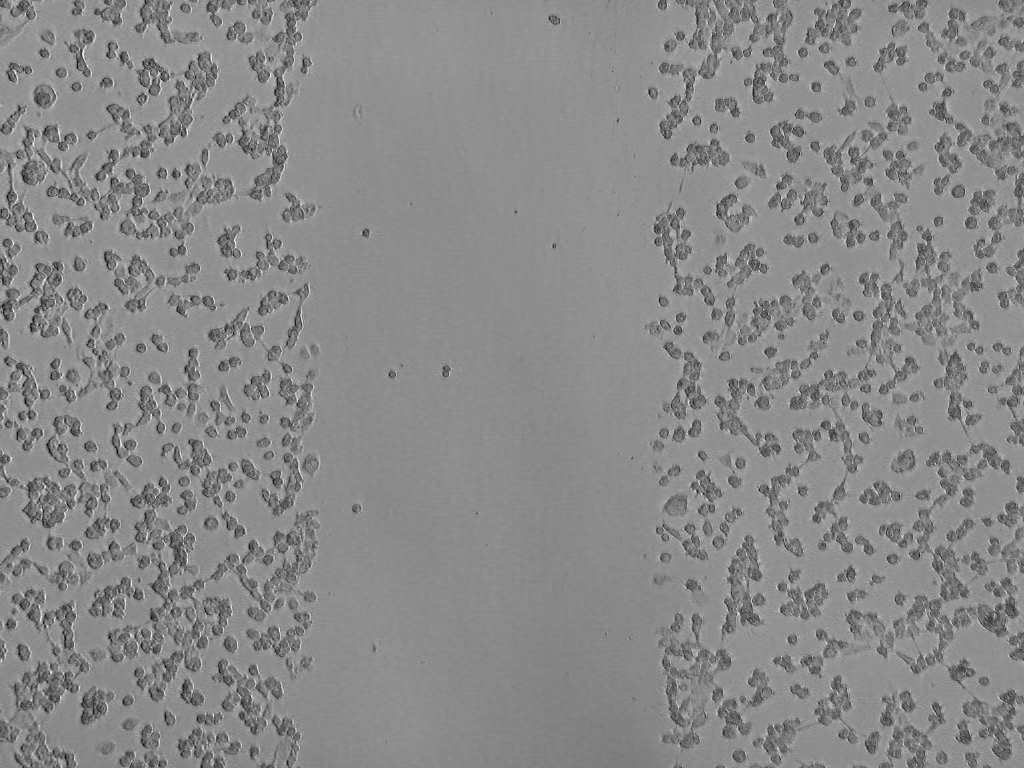

Supplement: Supplemental Information 5 [file peerj-11-16335-s005.zip › Raw data of Figures 9 E-F/peerj-86802-raw_data_of_figure9E-F-10.tiff]

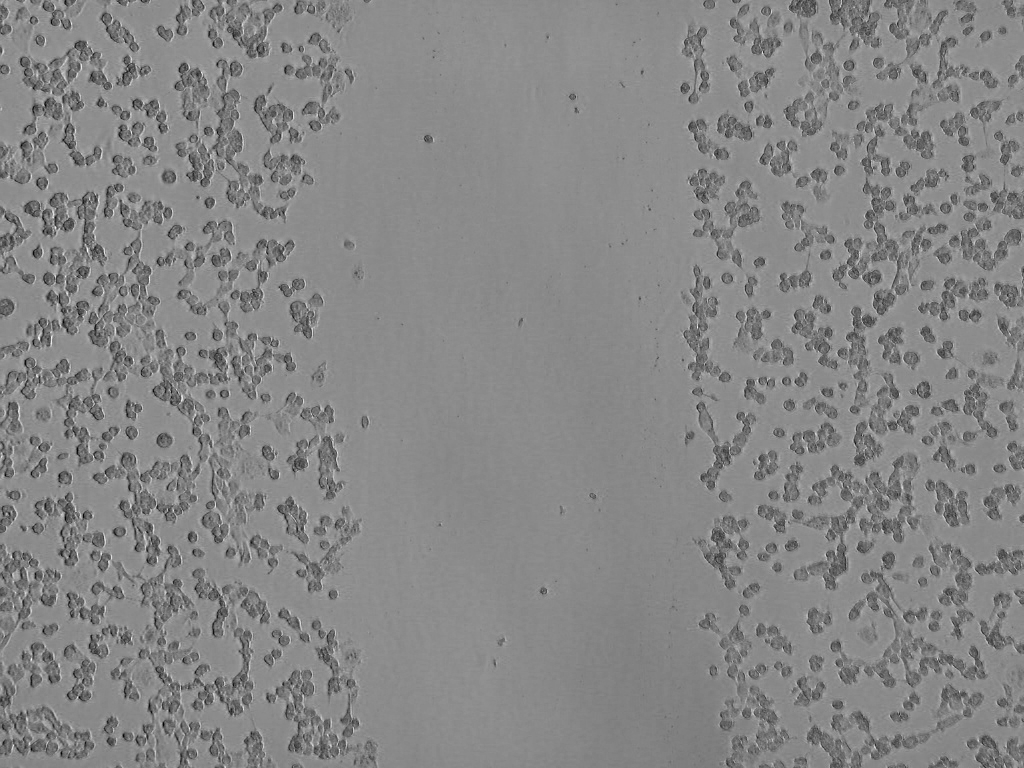

Supplement: Supplemental Information 5 [file peerj-11-16335-s005.zip › Raw data of Figures 9 E-F/peerj-86802-raw_data_of_figure9E-F-11.tiff]

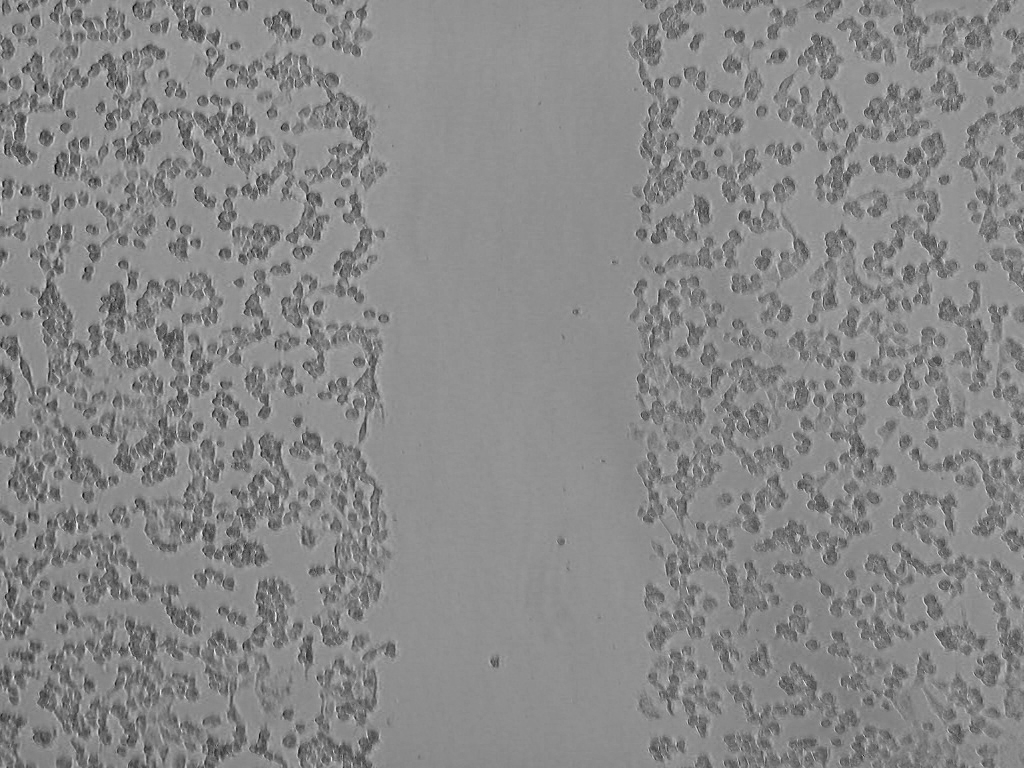

Supplement: Supplemental Information 5 [file peerj-11-16335-s005.zip › Raw data of Figures 9 E-F/peerj-86802-raw_data_of_figure9E-F-12.tiff]

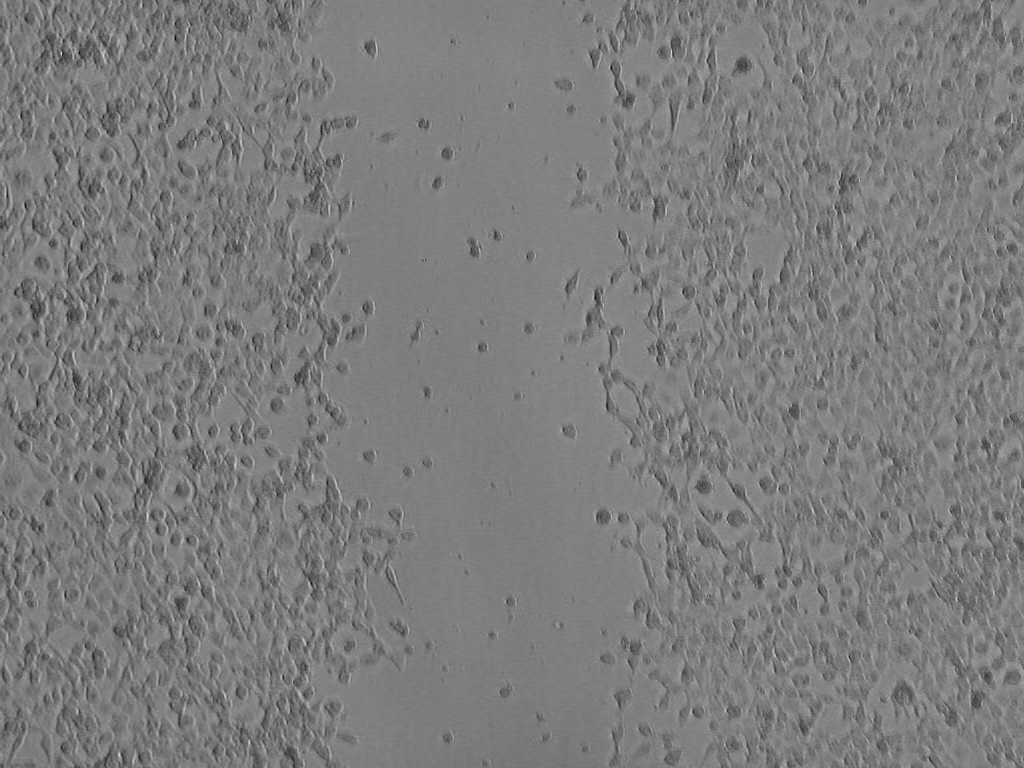

Supplement: Supplemental Information 5 [file peerj-11-16335-s005.zip › Raw data of Figures 9 E-F/peerj-86802-raw_data_of_figure9E-F-13.tiff]

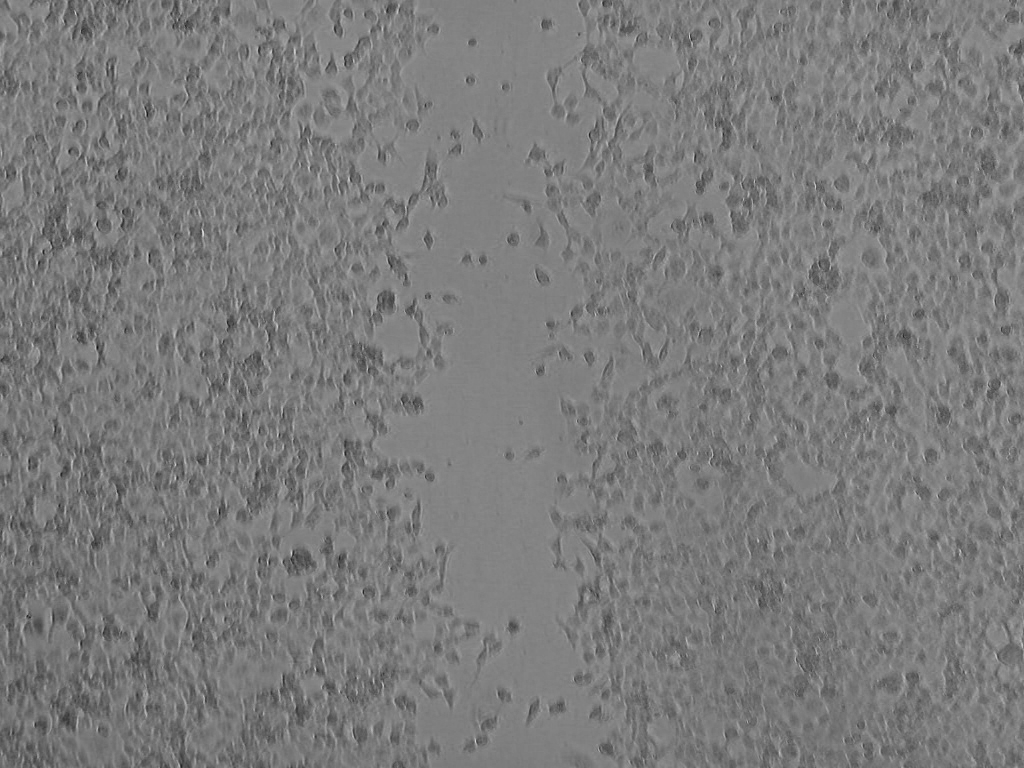

Supplement: Supplemental Information 5 [file peerj-11-16335-s005.zip › Raw data of Figures 9 E-F/peerj-86802-raw_data_of_figure9E-F-14.tiff]

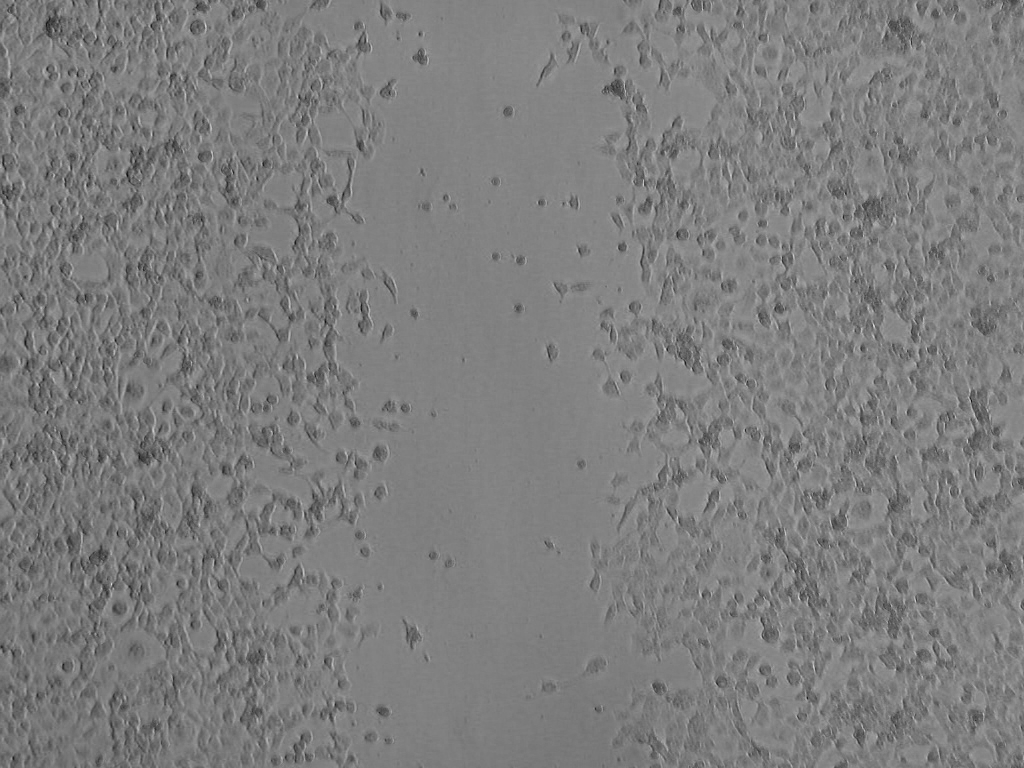

Supplement: Supplemental Information 5 [file peerj-11-16335-s005.zip › Raw data of Figures 9 E-F/peerj-86802-raw_data_of_figure9E-F-15.tiff]

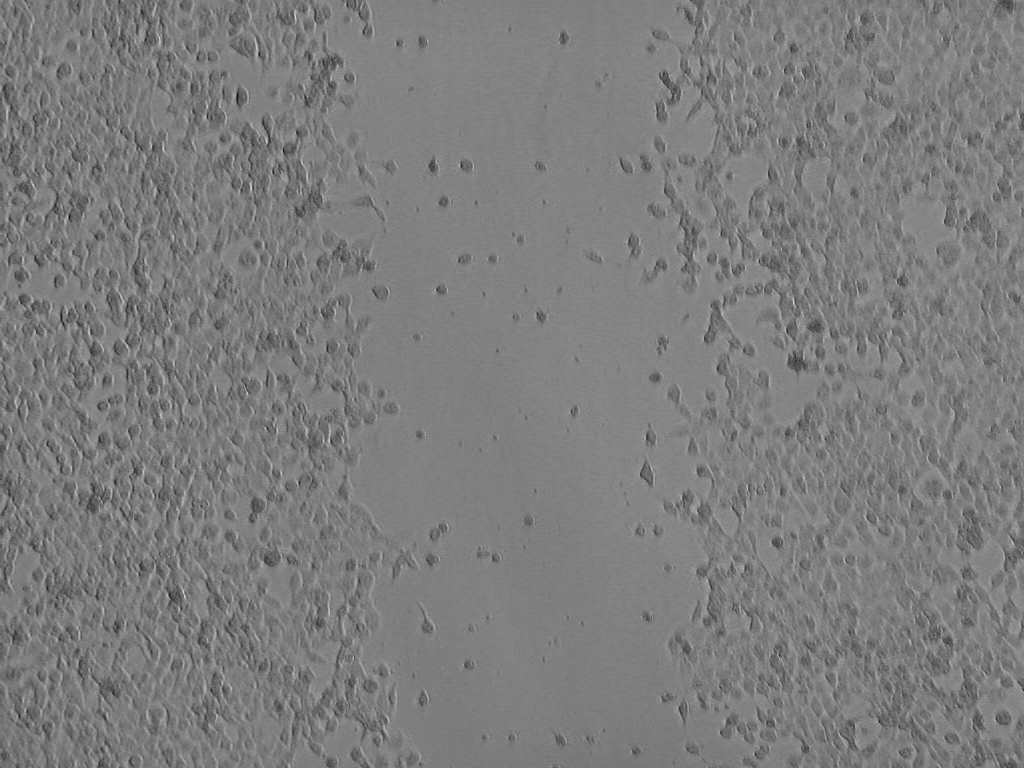

Supplement: Supplemental Information 5 [file peerj-11-16335-s005.zip › Raw data of Figures 9 E-F/peerj-86802-raw_data_of_figure9E-F-16.tiff]

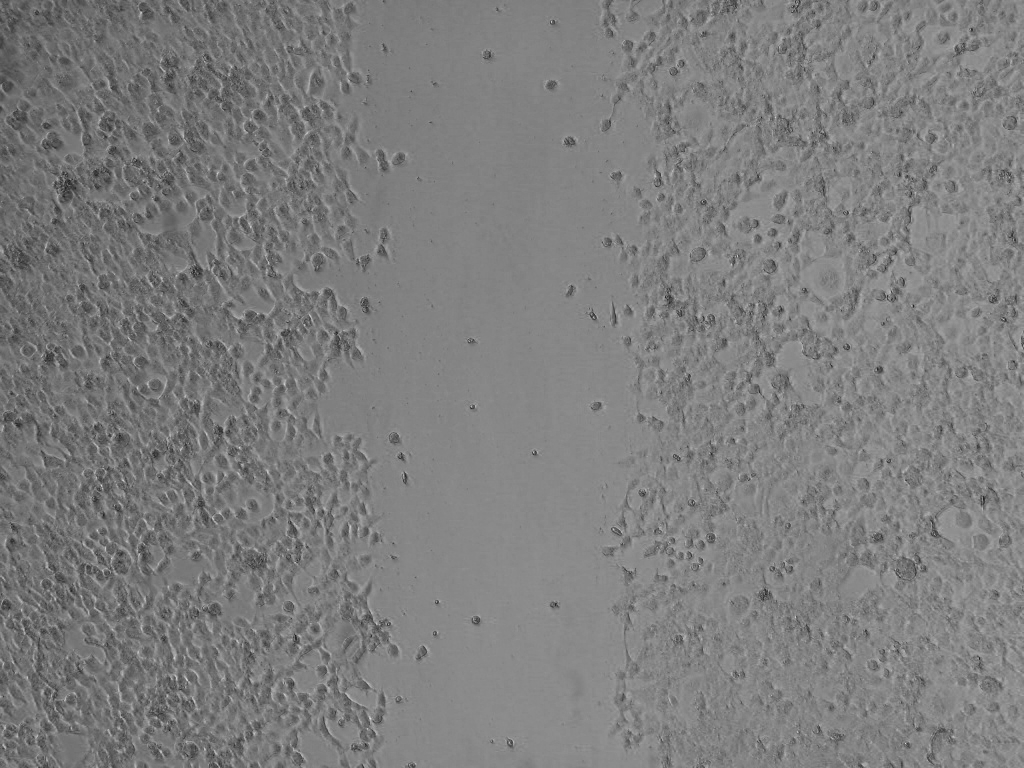

Supplement: Supplemental Information 5 [file peerj-11-16335-s005.zip › Raw data of Figures 9 E-F/peerj-86802-raw_data_of_figure9E-F-17.tiff]

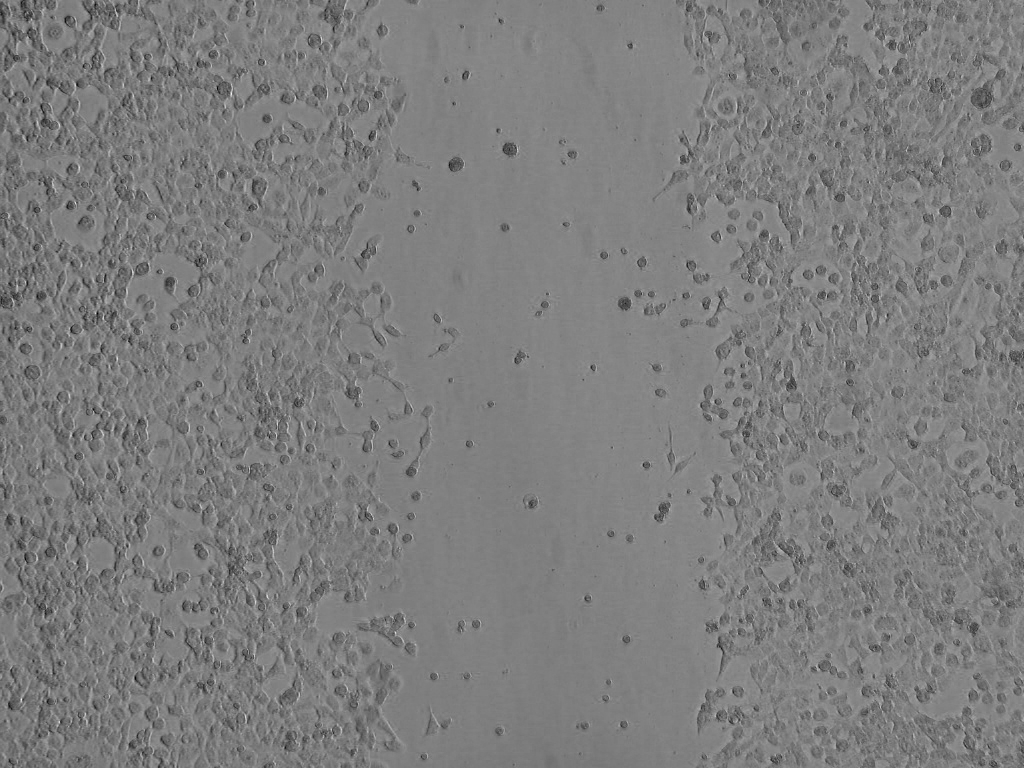

Supplement: Supplemental Information 5 [file peerj-11-16335-s005.zip › Raw data of Figures 9 E-F/peerj-86802-raw_data_of_figure9E-F-18.tiff]

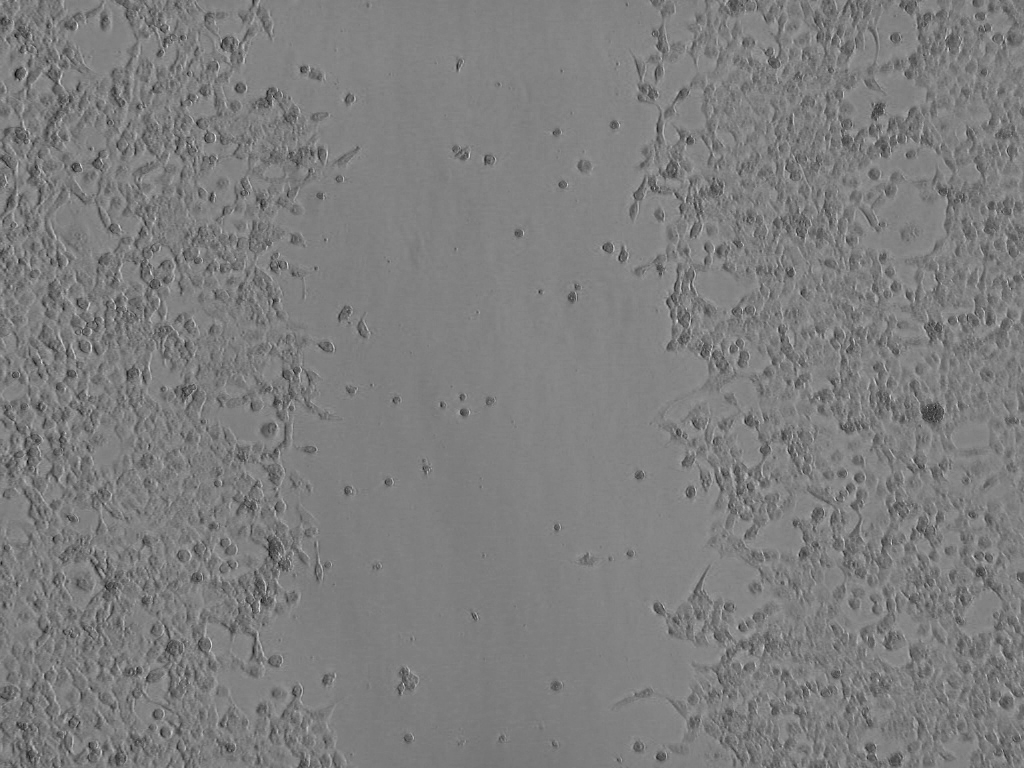

Supplement: Supplemental Information 5 [file peerj-11-16335-s005.zip › Raw data of Figures 9 E-F/peerj-86802-raw_data_of_figure9E-F-19.tiff]

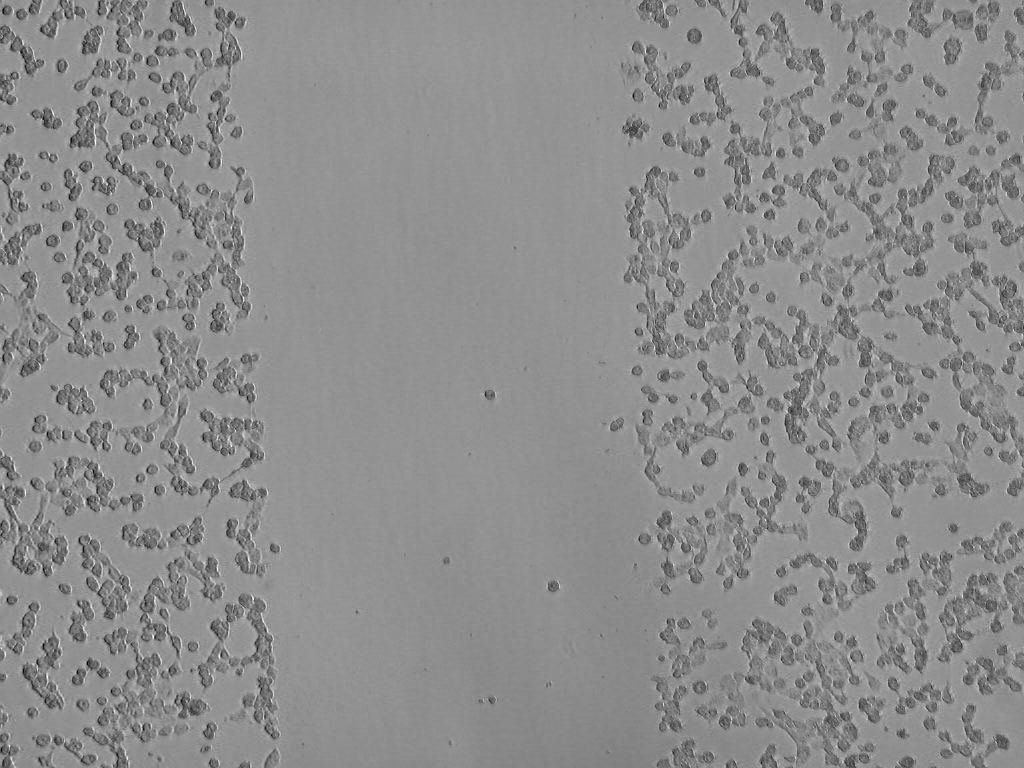

Supplement: Supplemental Information 5 [file peerj-11-16335-s005.zip › Raw data of Figures 9 E-F/peerj-86802-raw_data_of_figure9E-F-2.tiff]

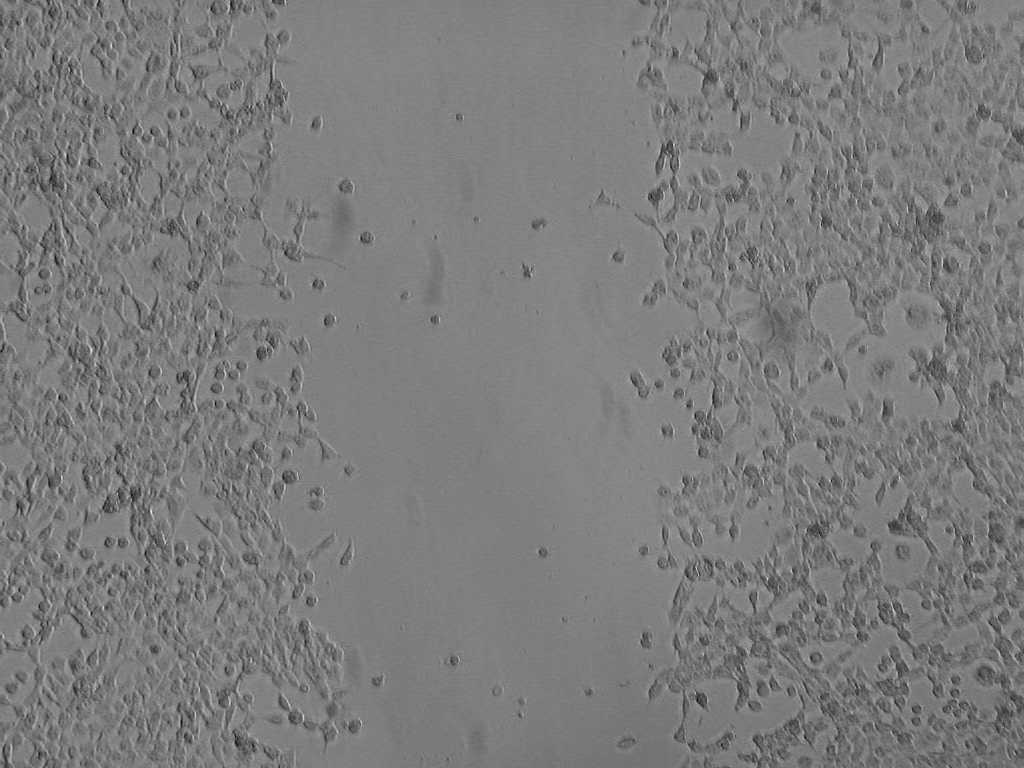

Supplement: Supplemental Information 5 [file peerj-11-16335-s005.zip › Raw data of Figures 9 E-F/peerj-86802-raw_data_of_figure9E-F-20.tiff]

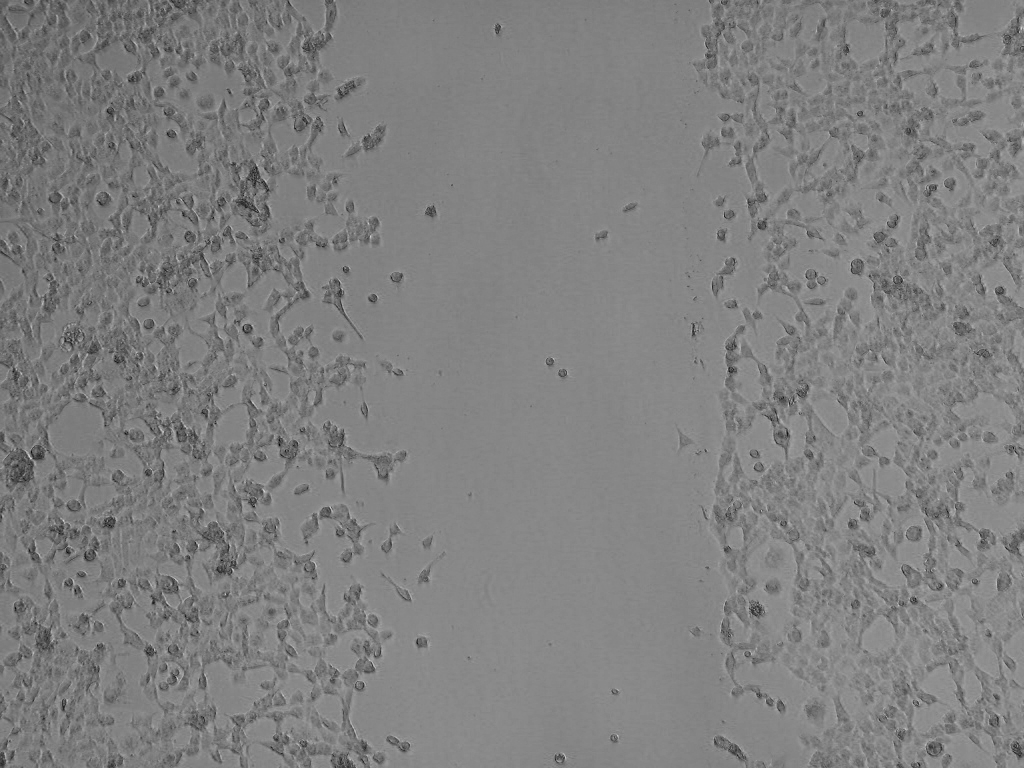

Supplement: Supplemental Information 5 [file peerj-11-16335-s005.zip › Raw data of Figures 9 E-F/peerj-86802-raw_data_of_figure9E-F-21.tiff]

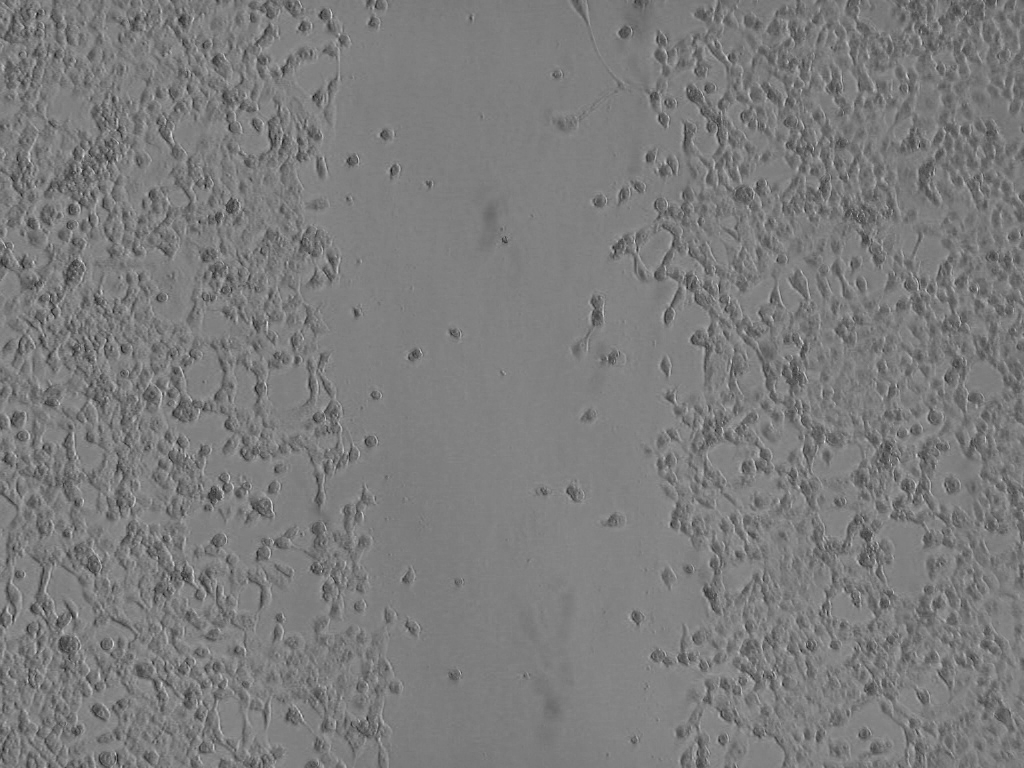

Supplement: Supplemental Information 5 [file peerj-11-16335-s005.zip › Raw data of Figures 9 E-F/peerj-86802-raw_data_of_figure9E-F-22.tiff]

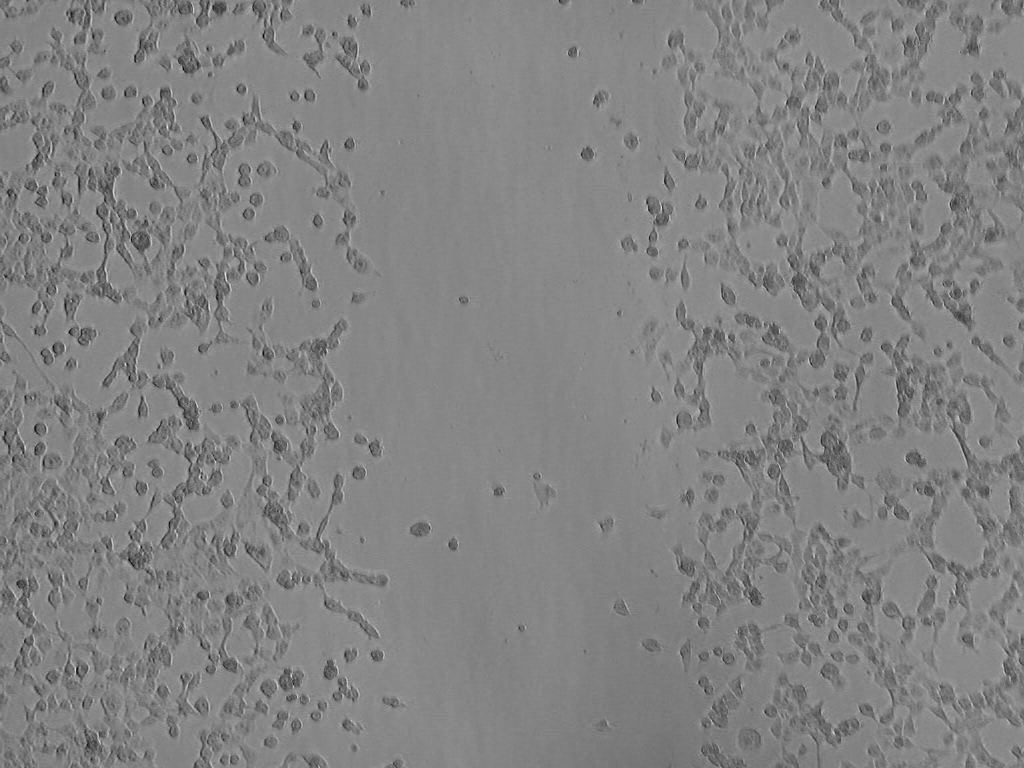

Supplement: Supplemental Information 5 [file peerj-11-16335-s005.zip › Raw data of Figures 9 E-F/peerj-86802-raw_data_of_figure9E-F-23.tiff]

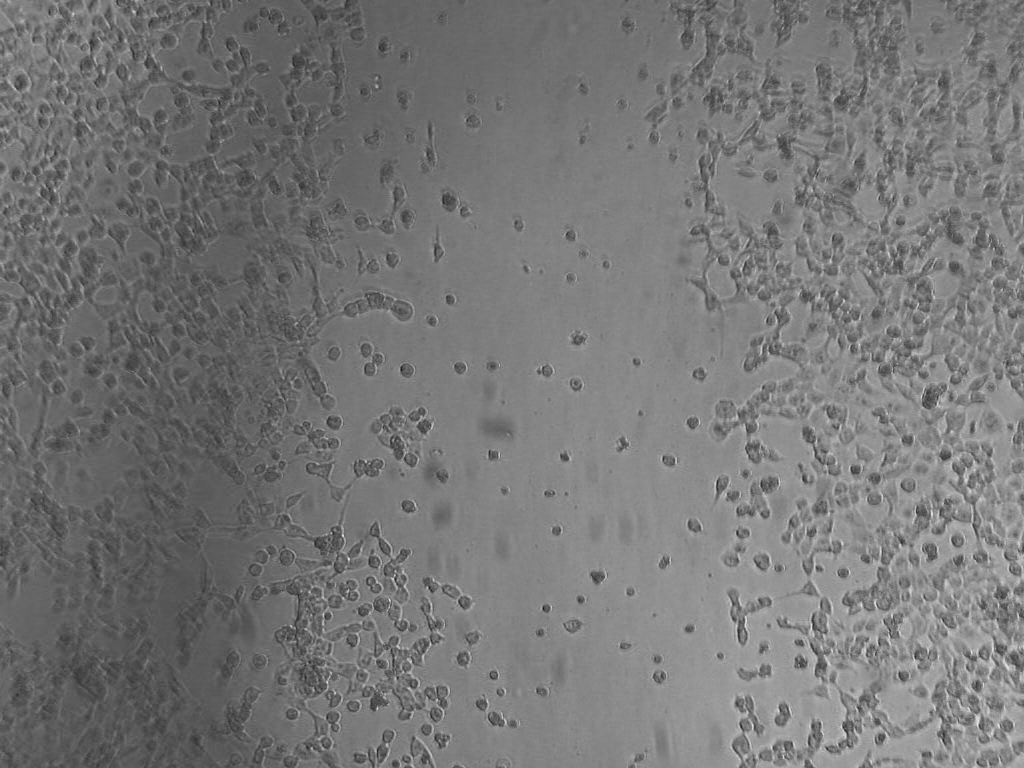

Supplement: Supplemental Information 5 [file peerj-11-16335-s005.zip › Raw data of Figures 9 E-F/peerj-86802-raw_data_of_figure9E-F-24.tiff]

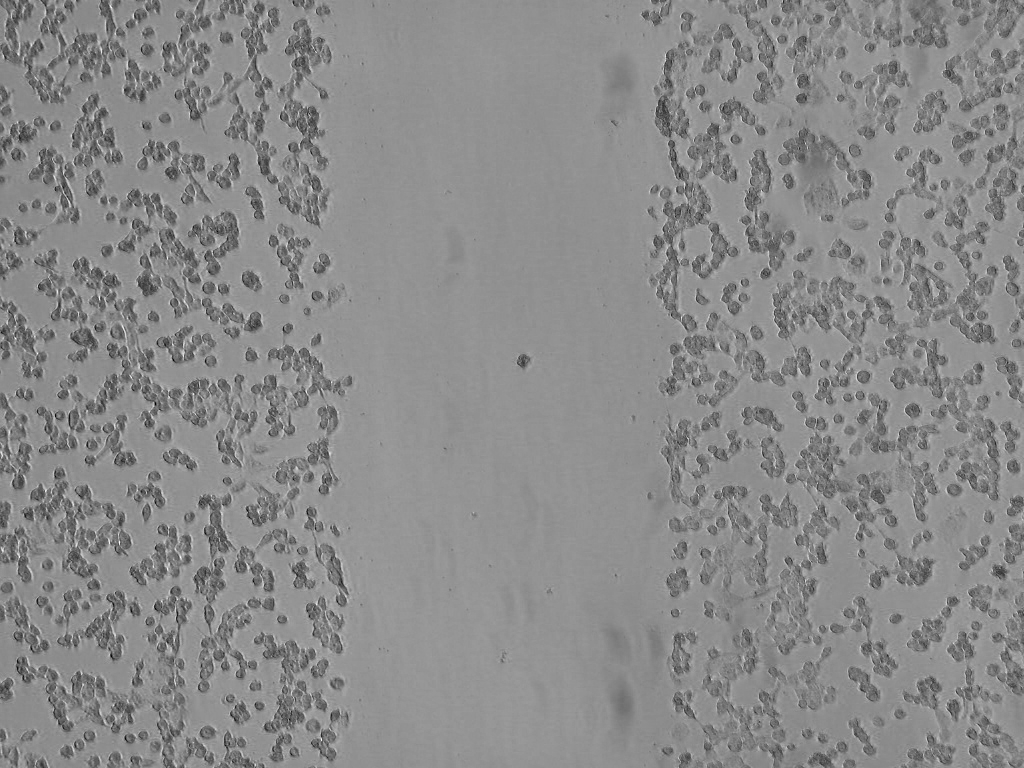

Supplement: Supplemental Information 5 [file peerj-11-16335-s005.zip › Raw data of Figures 9 E-F/peerj-86802-raw_data_of_figure9E-F-3.tiff]

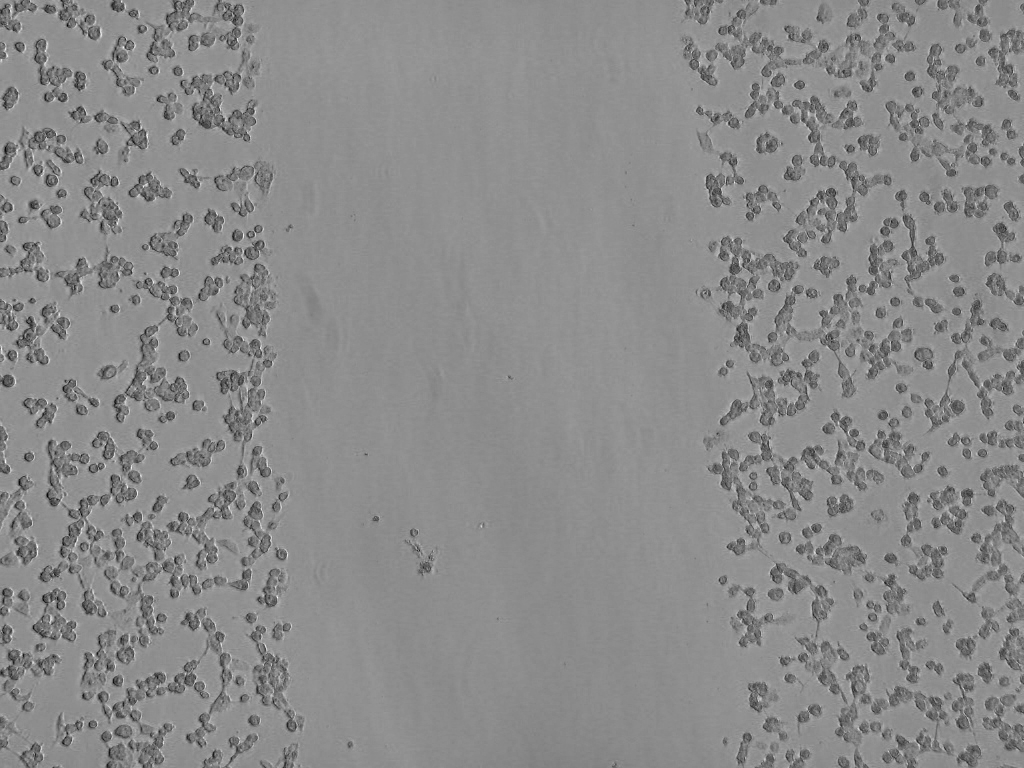

Supplement: Supplemental Information 5 [file peerj-11-16335-s005.zip › Raw data of Figures 9 E-F/peerj-86802-raw_data_of_figure9E-F-4.tiff]

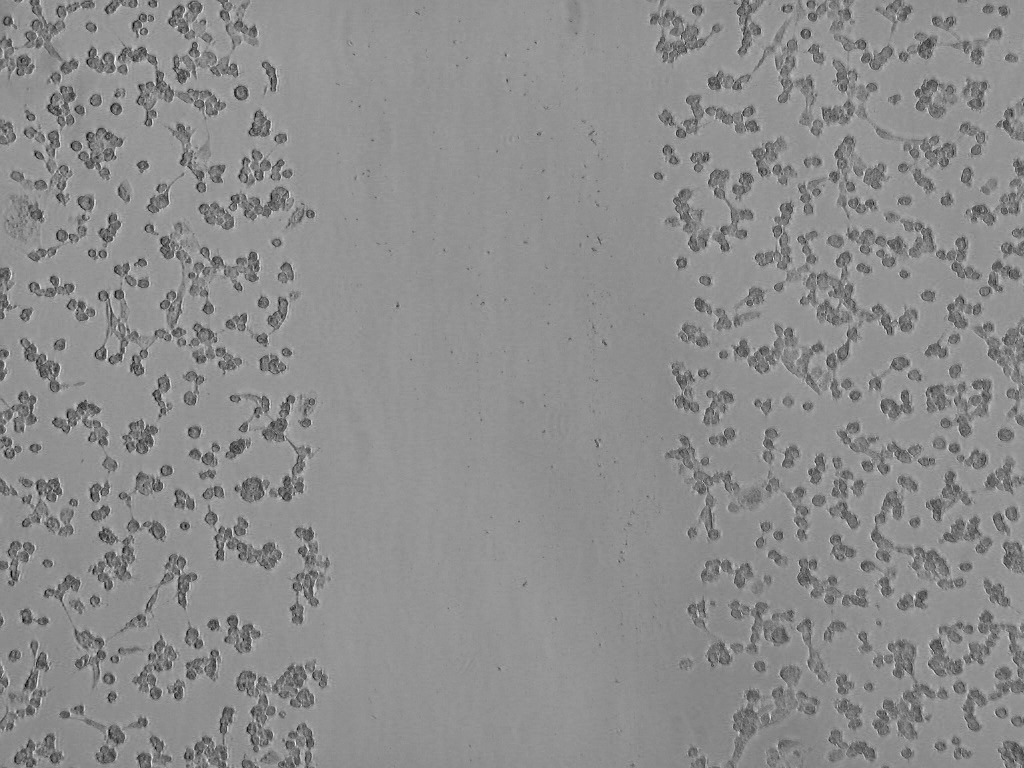

Supplement: Supplemental Information 5 [file peerj-11-16335-s005.zip › Raw data of Figures 9 E-F/peerj-86802-raw_data_of_figure9E-F-5.tiff]

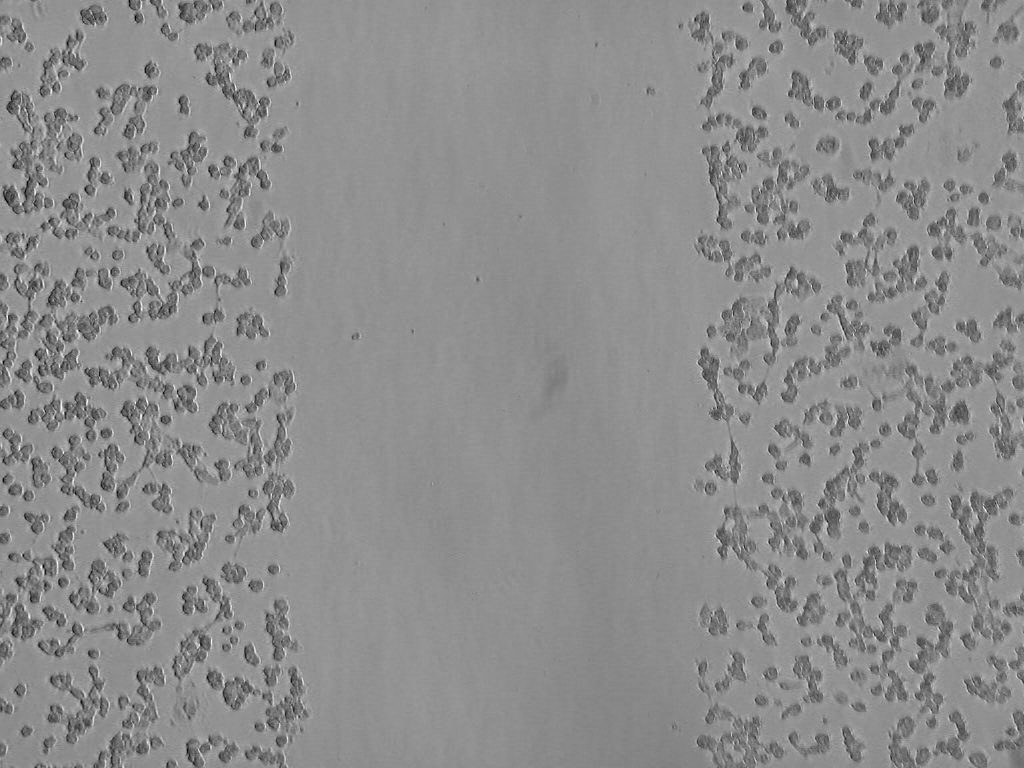

Supplement: Supplemental Information 5 [file peerj-11-16335-s005.zip › Raw data of Figures 9 E-F/peerj-86802-raw_data_of_figure9E-F-6.tiff]

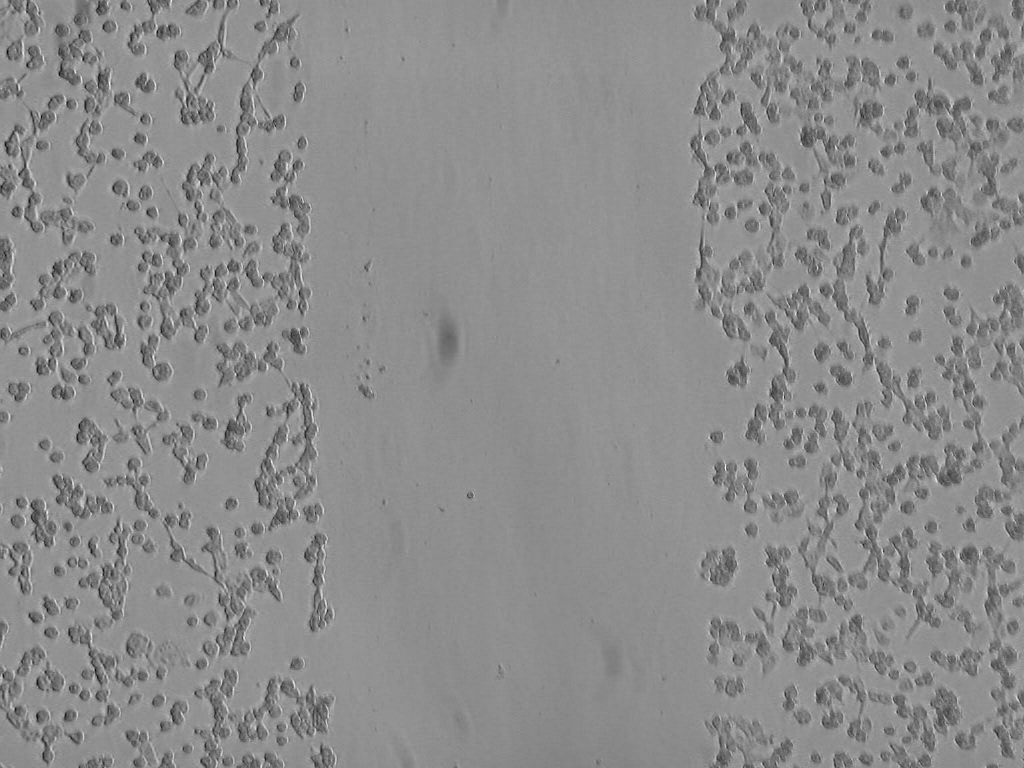

Supplement: Supplemental Information 5 [file peerj-11-16335-s005.zip › Raw data of Figures 9 E-F/peerj-86802-raw_data_of_figure9E-F-7.tiff]

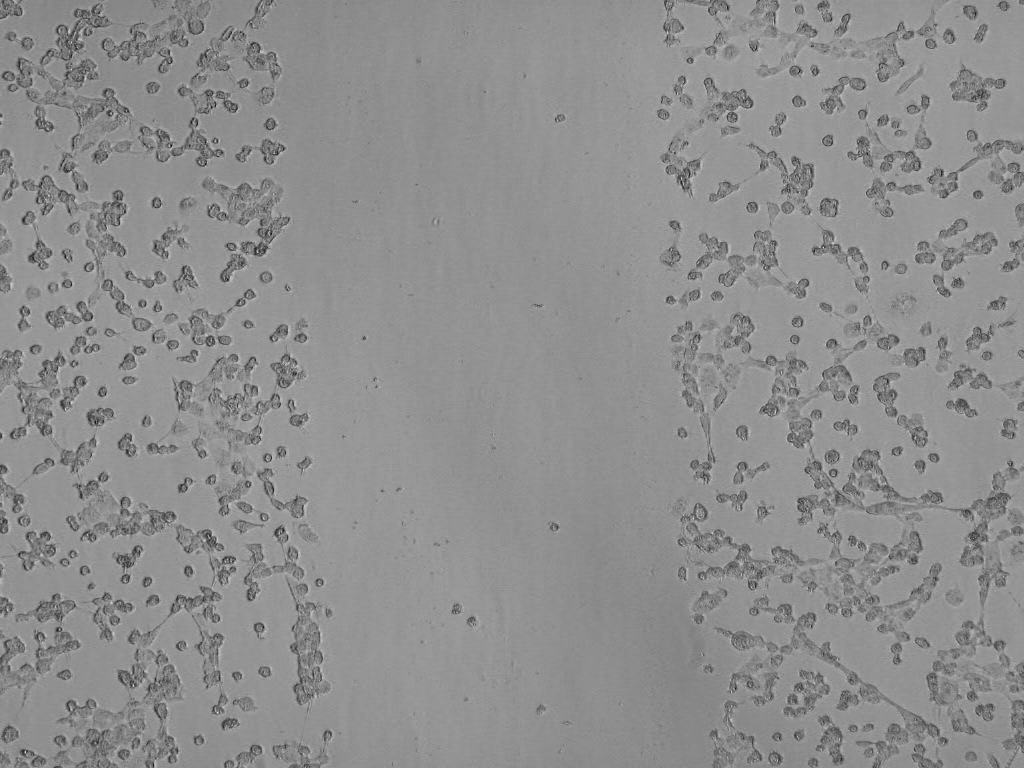

Supplement: Supplemental Information 5 [file peerj-11-16335-s005.zip › Raw data of Figures 9 E-F/peerj-86802-raw_data_of_figure9E-F-8.tiff]

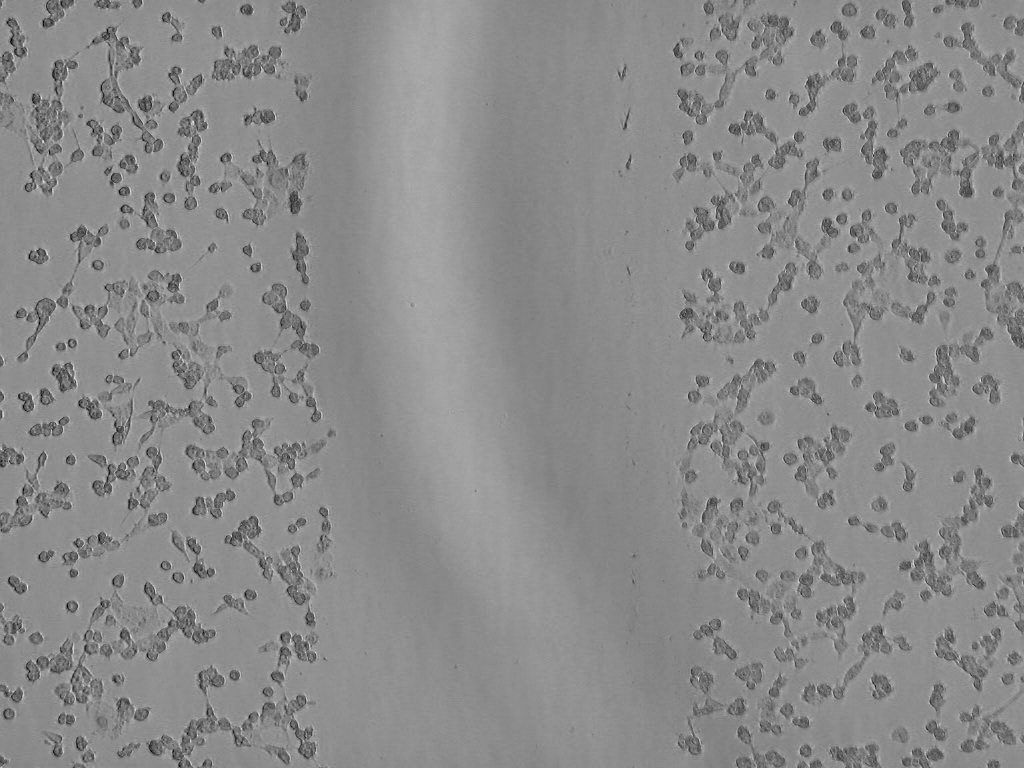

Supplement: Supplemental Information 5 [file peerj-11-16335-s005.zip › Raw data of Figures 9 E-F/peerj-86802-raw_data_of_figure9E-F-9.tiff]
